# Functional shell matrix proteins tentatively identified by asymmetric snail shell morphology

**DOI:** 10.1038/s41598-020-66021-w

**Published:** 2020-06-17

**Authors:** Akito Ishikawa, Keisuke Shimizu, Yukinobu Isowa, Takeshi Takeuchi, Ran Zhao, Keiji Kito, Manabu Fujie, Noriyuki Satoh, Kazuyoshi Endo

**Affiliations:** 1 Department of Earth and Planetary Science, Graduate School of Science, The University of Tokyo, 7-3-1 Hongo, Bunkyo, Tokyo, 113-0033 Japan; 2 Department of Applied Biological Chemistry, Graduate School of Agricultural and Life Sciences, The University of Tokyo, 1-1-1 Yayoi, Bunkyo, Tokyo, 113-8657 Japan; 3 Sugashima Marine Biological Laboratory, Graduate School of Science, Nagoya University, 429-63 Sugashima, Toba, Mie, 517-0004 Japan; 4 Marine Genomics Unit, Okinawa Institute of Science and Technology Graduate University, 1919-1 Tancha, Onna-son, Kunigami-gun, Okinawa, 904-0495 Japan; 5 Department of Life Sciences, School of Agriculture, Meiji University, 1-1-1 Higashimita, Tama, Kawasaki, Kanagawa, 214-8571 Japan; 6 DNA Sequencing Section, Okinawa Institute of Science and Technology Graduate University, 1919-1 Tancha, Onna-son, Kunigami-gun, Okinawa, 904-0495 Japan

**Keywords:** Evolutionary developmental biology, Proteomics, Transcriptomics

## Abstract

Molluscan shell matrix proteins (SMPs) are essential in biomineralization. Here, we identify potentially important SMPs by exploiting the asymmetric shell growth in snail, *Lymnaea stagnalis*. Asymmetric shells require bilaterally asymmetric expression of SMP genes. We examined expression levels of 35,951 transcripts expressed in the left and right sides of mantle tissue of the pond snail, *Lymnaea stagnalis*. This transcriptome dataset was used to identify 207 SMPs by LC-MS/MS. 32 of the 207 SMP genes show asymmetric expression patterns, which were further verified for 4 of the 32 SMPs using quantitative PCR analysis. Among asymmetrically expressed SMPs in dextral snails, those that are more highly expressed on the left side than the right side are 3 times more abundant than those that are more highly expressed on the right than the left, suggesting potentially inhibitory roles of SMPs in shell formation. The 32 SMPs thus identified have distinctive features, such as conserved domains and low complexity regions, which may be essential in biomineralization.

## Introduction

Biomineralization is the process by which organisms incorporate and deposit minerals. The end products, called biominerals, are composed of both minerals and organic matrices, which are considered essential to formation of highly ordered, functional materials^1,2^. In these organic matrices, proteins are the major components and have attracted much interest. SM50, from sea urchin larval spicules, was the first such protein sequenced among calcium carbonate biominerals^3^. Subsequently, studies of molluscan shell proteins identified such matrix proteins as the carbonic anhydrase nacrein^4^ and the probable shell framework proteins, MSI60 and MSI31^5^, from the pearl oyster, *Pinctada fucata*. The following decade saw a significant surge in sequence determination of skeletal matrix proteins^6–8^.

Recent advances of analytical techniques brought even more drastic changes in our understanding of matrix proteins. Proteomic analyses combined with genomic and transcriptomic analyses made it possible to almost comprehensively characterize protein sequences from biominerals. These advances triggered a burst of novel matrix proteins identified from various biominerals, including chicken eggshells^9^, sea urchin larval spicules^10^, shells of molluscs^11–21^, and brachiopods^22–24^. In these studies, literally hundreds of proteins have been identified from biominerals of individual species.

**Figure 1:**
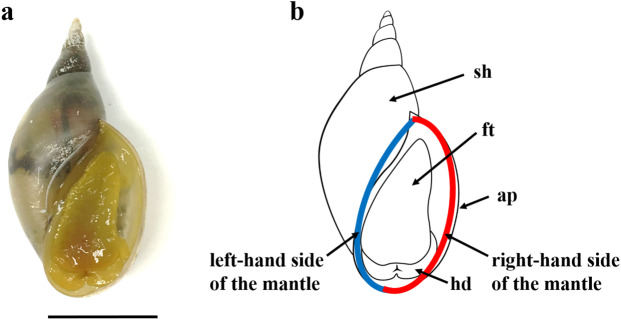
An adult individual of *Lymnaea stagnalis* showing the position of mantle tissues dissected for analysis. (**a**) Ventral view of *L. stagnalis*. (**b**) A schematic diagram of *L. stagnalis*. Red line: right side, blue line: left side of the mantle, ap: shell aperture, ft: foot, hd: head, and sh: shell. Scale bar = 1 cm.

These techniques enabled a new era of proteomic biomineralization studies; however, they also raised a conundrum. Previous studies identified proteins that are not specific to biomineralization, e.g., house-keeping proteins such as EF-1α and ribosomal proteins^11^. Do all these matrix proteins function in biomineralization? Is there a way to identify essential SMPs among the literally hundreds of SMPs identified by omics approaches? To address these questions, we focused our attention on the pond snail, *Lymnaea stagnalis* (Fig. 1a). Because these snails produce coiled-shells, which can only be produced by asymmetric accretion of shell material to the shell aperture, we hypothesized that some genes responsible for shell formation may be differentially expressed between the left and right sides of their mantle tissues. In other words, by comparing gene expression levels between the left and right sides of the mantle, we anticipated being able to identify functionally important proteins. We posited that enhanced and diminished SMP expression would result in more biomineralization on the right side than left side of the mantle in dextral snails (Fig. 1b).

In addition, *L. stagnalis* is an ideal organism for such a study because it has a short life cycle and can be reared easily in the laboratory. For this reason, it has been used for a wide range of studies, including neurophysiology, embryology, and environmental toxicology^25–28^. At least three previous studies have used transcriptomic analyses to understand the snail central nervous system or responses to a pesticide^29–31^. Shell matrix proteins (SMPs) have also been characterized in *L. stagnalis*^6,32^. Herlitze *et al*. (2018) identified 34 candidate shell-forming proteins, showing that their transcripts display a variety of spatial and temporal expression patterns at different developmental stages^32^. Therefore, *L. stagnalis* is an ideal ‘model organism’ for biomineralization.

In this study, we identified shell matrix proteins of *L. stagnalis* using a combination of proteomic and transcriptomic analyses. Gene expression levels of shell matrix proteins have been compared between the right and left sides of the mantle. We identified 32 shell matrix protein genes that are asymmetrically expressed in the mantle transcriptome, suggesting their roles in shell formation in this species. Using quantitative PCR analysis, asymmetric expression patterns were further verified for four of these 32 SMPs. The shell proteomic and transcriptomic data presented here may support additional studies of biomineralization mechanisms, as well as evolutionary processes of shell formation in molluscs.

## Results

### Mantle transcriptomic analysis

Approximately 70 million reads were obtained for each of the 6 paired-end libraries prepared from left and right mantle tissues of three biological replicates. The read length was 200 bp and GC content was ~40% (Table 1). Sequence assemblages using all six pairs of samples from mantle tissues generated 337,195 contigs with a maximum contig length of 37,809 bp, an average length of 1,140 bp, and an N50 value of 2,828 bp. Local BLASTN searches of these contigs against the whole genome shotgun sequence of *L. stagnalis* (GCA_900036025.1, unpublished, Ashworth Laboratories, 2016) returned significantly similar sequences for 309,623 contigs (e-value <10^−10^). After an ORF search by TransDecoder, 162,121 contigs remained in the FASTA file with a maximum contig length of 32,196 bp, an average length of 1,050 bp, and an N50 value of 1,728 bp. After clustering with CD-HIT, 35,951 sequences remained with a maximum sequence length of 32,196 bp, an average length of 1,190 bp, and an N50 value of 1,974 bp. After clustering with CD-HIT, we used those sequences as references for the proteomic analysis. The sequence assembly, gene set, and transcriptome completeness of the FASTA file have been checked using BUSCO statistics^33,34^. The results indicated that our contig sequences are well assembled and comprise a nearly complete gene set that identified 99.1% (969) complete genes and 0.6% (6) fragmented genes among the 978 metazoan BUSCO genes (Supplementary Table S1).

**Table 1:** Details of transcriptomic data obtained in this study.

File name	Source of RNA	Direction of Paired-end reads	Number of reads in total	Sequence length	GC content (%)	DRA Accession number
Sample07_TAGCTT_ALL_R1_001.fastq	Left side of mantle from individual #1	Forward	75,993,110	101	39	SAMD00074112
Sample07_TAGCTT_ALL_R2_001.fastq	Left side of mantle from individual #1	Reverse	75,993,110	101	39	SAMD00074112
Sample08_GGCTAC_ALL_R1_001.fastq	Left side of mantle from individual #2	Forward	68,329,443	101	37	SAMD00074113
Sample08_GGCTAC_ALL_R2_001.fastq	Left side of mantle from individual #2	Reverse	68,329,443	101	37	SAMD00074113
Sample09_GTGGCC_ALL_R1_001.fastq	Left side of mantle from individual #3	Forward	66,846,658	101	39	SAMD00074114
Sample09_GTGGCC_ALL_R2_001.fastq	Left side of mantle from individual #3	Reverse	66,846,658	101	39	SAMD00074114
Sample10_GTTTCG_ALL_R1_001.fastq	Right side of mantle from individual #1	Forward	69,768,907	101	39	SAMD00074115
Sample10_GTTTCG_ALL_R2_001.fastq	Right side of mantle from individual #1	Reverse	69,768,907	101	39	SAMD00074115
Sample11_CGTACG_ALL_R1_001.fastq	Right side of mantle from individual #2	Forward	70,252,413	101	40	SAMD00074116
Sample11_CGTACG_ALL_R2_001.fastq	Right side of mantle from individual #2	Reverse	70,252,413	101	40	SAMD00074116
Sample12_GAGTGG_ALL_R1_001.fastq	Right side of mantle from individual #3	Forward	68,107,519	101	39	SAMD00074117
Sample12_GAGTGG_ALL_R2_001.fastq	Right side of mantle from individual #3	Reverse	68,107,519	101	39	SAMD00074117
Ls8_S3_L001_R1_001.fastq	Foot from individual #4	Forward	7,127,862	35–309	42	SAMD00106507
Ls8_S3_L001_R2_001.fastq	Foot from individual #4	Reverse	7,127,862	35–309	42	SAMD00106507

**Figure 2:**
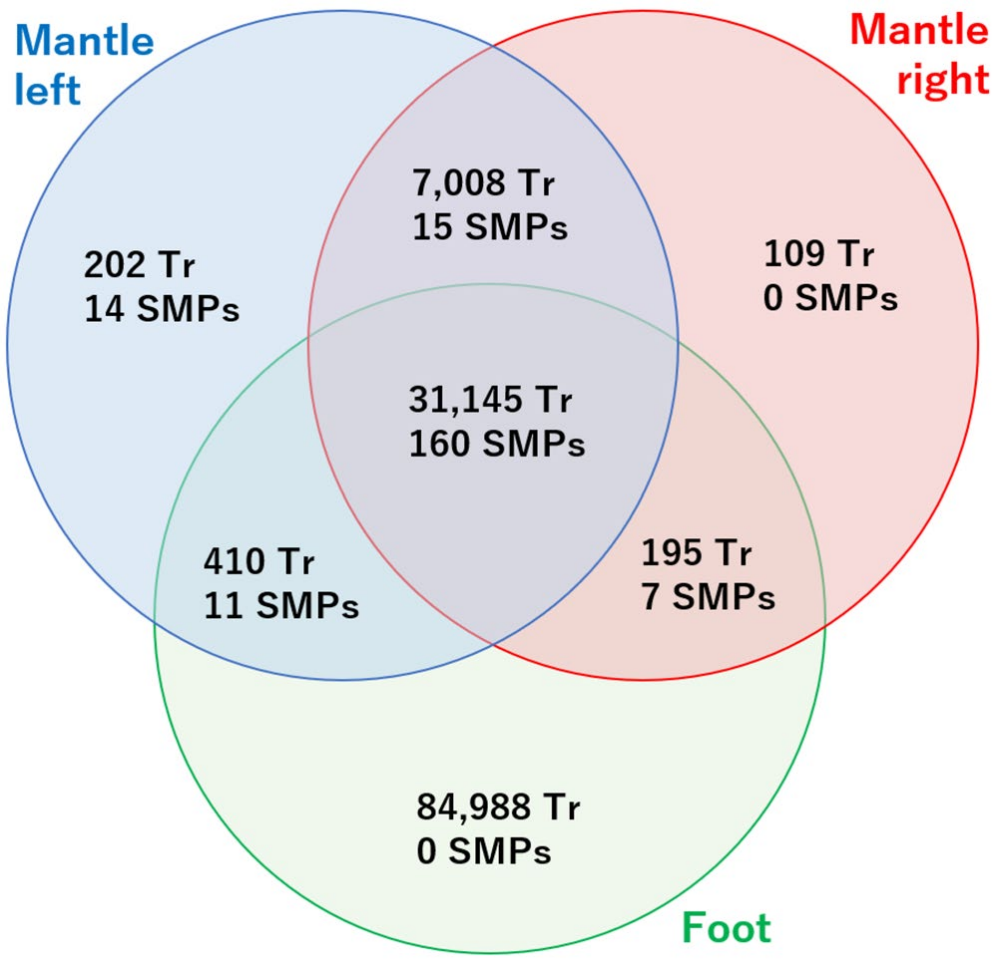
Venn diagram showing numbers of transcripts and SMPs identified in right mantle, left mantle, and foot using transcriptomic and proteomic analyses. Tr: transcripts.

About 7 million reads with read lengths of 35–309 bp and a GC content of 42% were obtained as paired-end sequences for foot tissue (Table 1). After removal of low quality reads, 5,965,429 reads (paired-end pairs) remained in the FASTQ file (Table 1). BUSCO statistics for the FASTA file obtained for the foot transcriptome (116,738 contig sequences; Fig. 2) identified 93.7% (917) complete genes and 5.7% (56) fragmented genes, a value slightly lower than, but comparable to the value for the mantle transcriptome shown above (Supplementary Table S2).

### Proteomic analysis

LC-MS/MS identified 378 unique peptide fragments from the shell matrix of *L. stagnalis*. Of these 378 peptide fragments, 91,233, and 55 peptides were identified from the soluble, insoluble, and both soluble and insoluble fractions, respectively (Supplementary Table S3). Using transcriptomic data obtained from mantle tissues of *L. stagnalis*, protein sequences identified by more than one unique peptide were employed in subsequent analyses. In all, 207 proteins were identified with 21, 116, and 70 having been identified in the soluble, insoluble, and both fractions, respectively (Supplementary Table S4).

### Analysis of SMP-encoding transcripts

147 contigs encoded complete protein sequences, accounting for 71% of the 207 shell matrix proteins of *L. stagnalis*. In this study, a complete sequence refers to a gene model that has both start and stop codons. Of the remaining 60 sequences, 16, 31, and 13 had the 3′ end missing, the 5′ end missing, or internal sequences, respectively (Supplementary Table S4). The distribution of theoretical isoelectric points (pI) estimated for all complete sequences of SMPs identified in this study indicated a bimodal pattern with acidic proteins being more numerous than basic ones (Supplementary Fig. S1). The highest and lowest pIs were 10.90 and 3.65, respectively.

### Similarity searches using BLAST

In order to find homologous sequences in the databases, the 207 SMPs identified in this study were searched against GenBank using BLASTP, and 165 proteins showed similarity to known proteins. Of the 165 proteins, 156 and 9 SMPs indicated high similarity to those molluscs and other invertebrates, and even to vertebrates. The remaining 42 SMPs are novel proteins, which are dissimilar from all known proteins (Supplementary Table S4).

### Conserved domain search

**Figure 3:**
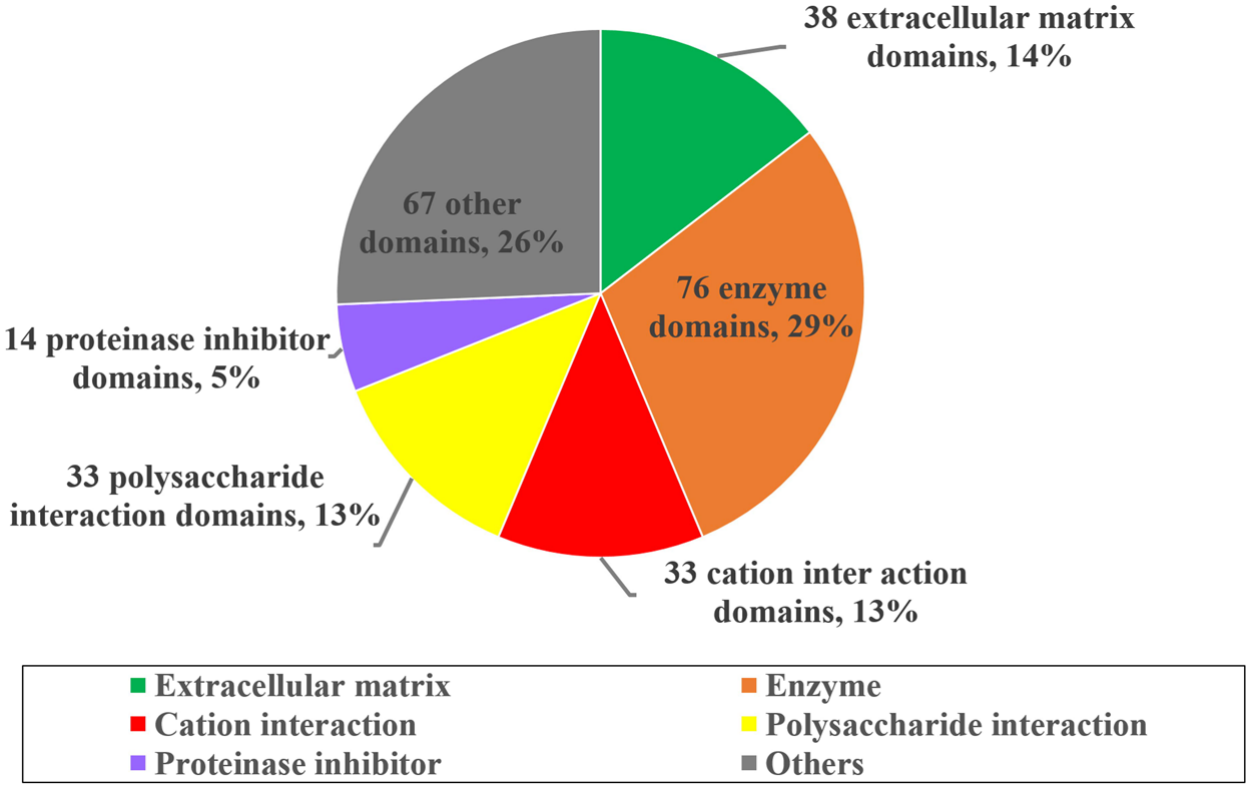
Summary of domains identified from SMPs of *L. stagnalis*. Actual counts and frequencies of different kinds of domains observed among the 207 SMPs are shown.

Searches for conserved domains using SMART identified 261 domains in the 207 SMPs. Those domains were grouped into six categories: extracellular matrix (38 domains), enzyme (76), cation interaction (33), polysaccharide interaction (33), proteinase inhibitor (14), and others (67) (Fig. 3).

115 proteins had signal peptides, including 99 complete amino acid sequences. Among the 115 proteins were 22 house-keeping, 17 room-keeping, 8 known SMPs, 37 uncharacterized proteins, and 31 novel proteins (Supplementary Table S4). The 99 SMPs with signal peptides and complete sequences included 17 house-keeping, 13 room-keeping, 7 SMPs, 36 uncharacterized proteins, and 26 novel proteins. Of the 92 proteins lacking signal peptides there were 48 complete and 44 partial sequences. Among them 48 house-keeping proteins, 16 room-keeping proteins, 5 SMPs, 12 uncharacterized proteins, and 11 novel proteins (Supplementary Table S4). The 48 complete SMP sequences without signal peptides included 29 house-keeping proteins, 8 room-keeping proteins, 3 SMPs, 5 uncharacterized proteins, and 3 novel proteins.

Eighty-one proteins contained one or more low complexity regions (LCRs). Of these, 14, 13, 6, 24, and 24 proteins were identified as house-keeping, room-keeping, SMP, uncharacterized protein, and novel proteins, respectively (Supplementary Table S4).

Comparisons of shared domains among the SMPs of three molluscs, *L. stagnalis*, *Lottia gigantea*, and *Crassostrea gigas*: (the latter two have decoded draft genomes^11,35^) indicated that 11 kinds of domains are shared among these species, including 5 extracellular regions, 3 polysaccharide interaction domains, 2 enzymes, and 1 cation interaction domain (Supplementary Fig. S2).

### Comparison of SMP expression levels between the right and left sides of mantle tissues

**Figure 4:**
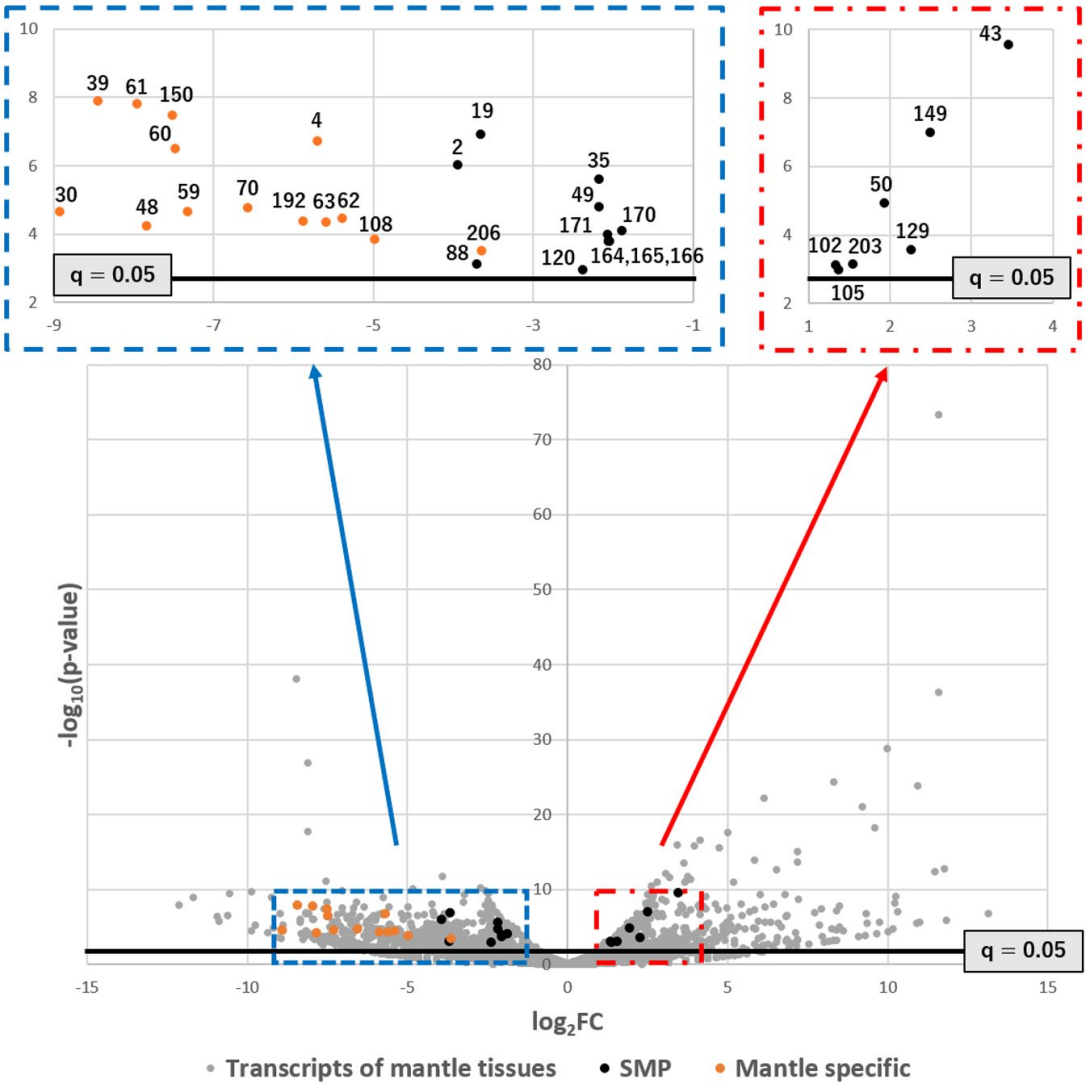
Volcano plot showing differential expression of SMP-coding genes between right and left sides of mantle tissues. The X axis represents the logarithm of the change in expression levels of the right side vs. the left side. The Y axis represents the logarithm of the significance level for each comparison of the gene. The level of significance to reject the null hypothesis (q = 0.05) is shown as a red line. Numbers denote serial numbers of contigs (genes) in Supplementary Table S4. Only genes that were considered significant or expressed specifically in the mantle (shown in orange dots) are numbered.

**Figure 5:**
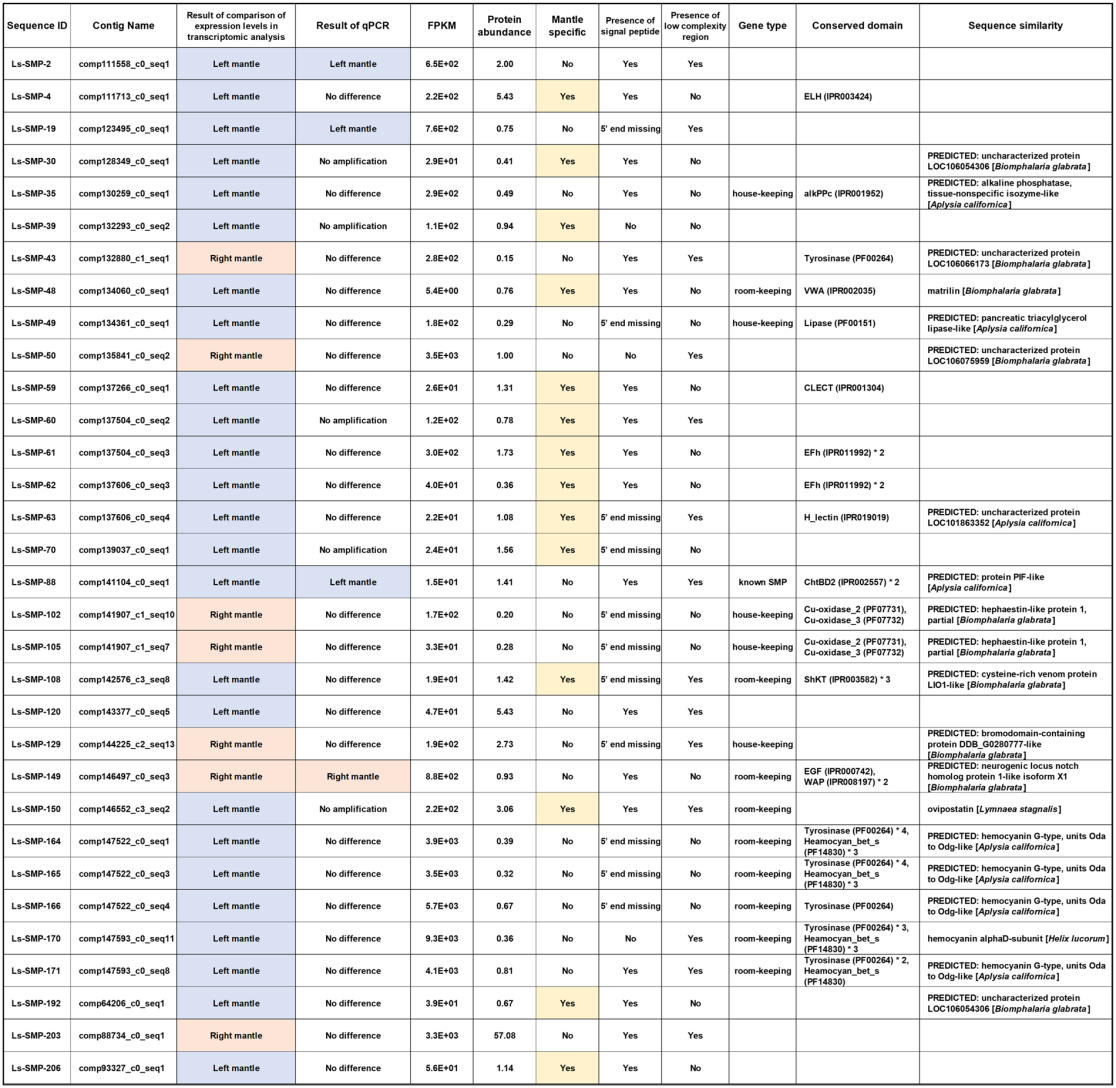
Features of the 32 SMPs displaying a significant difference in gene expression between left and right sides of the mantle. “Room-keeping” genes are those specific to a particular functional unit of the body, such as the nervous system, blood, or the immune system. See Supplementary Table S7 for further details of those 32 SMPs.

The right and left sides of the mantle tissues of *L. stagnalis* should have different rates of shell growth to produce the asymmetric dextral shell (Fig. 1). Levels of shell matrix protein gene expression have been compared between the right and left sides of the mantle in three individuals, to identify functionally important proteins (Supplementary Tables S5 and S6). There are indeed differences in the patterns of gene expression between the left and right sides of the mantle. Of the 35,951 transcript sequences identified from the mantle, 916 transcripts (2.6%) indicated a statistically significant difference between the left and right sides, considering variations in gene expression levels among the three individuals studied (q < 0.05; Fig. 4, Supplementary Table S6). Of those 916 transcripts, 32 transcripts encode SMPs (15.5% of the 207 SMPs identified in this study). Among the 916 transcripts, 612 encoding proteins other than SMPs (64.4%), and 25 encoding SMPs (78.1% of 32) showed higher expression on the left side than on the right (Fig. 5). Among the 32 SMPs, 25 showed higher expression on the left than the right, with Ls-SMP-30 showing the greatest difference in expression level. In contrast, 7 SMPs showed higher expression on the right side than the left, with Ls-SMP-43 being the most distinctively different (Figs. 2, 4 and Supplementary Table S4).

The mantle transcriptome was compared to the foot transcriptome using local BLAST to find genes that are expressed in the mantle, but not in the foot (E value cut off >10^−10^) (genes hereafter termed “mantle specific”). Of the 207 SMP genes, 29 were mantle specific (Fig. 5 and Supplementary Table S7). 14 of the 29 mantle-specific SMP genes were significantly more highly expressed on the left side than the right. Those 14 genes encode ovipostatin (Ls-SMP-150), matrilin-like (Ls-SMP-48), cysteine-rich venom protein LO1-like protein (Ls-SMP-108), three uncharacterized proteins (Ls-SMP-30, Ls-SMP-63, and Ls-SMP-192), and 8 novel proteins (Ls-SMP-4, Ls-SMP-39, Ls-SMP-59, Ls-SMP-60 Ls-SMP-61, Ls-SMP-62, Ls-SMP-70, and Ls-SMP-206). The remaining 15 genes did not show asymmetric expression patterns, and include 5 SMP genes containing conserved domains found in other molluscs (Ls-SMP-23, Ls-SMP-24, Ls-SMP-81, Ls-SMP-82, and Ls-SMP-186) (Supplementary Table S4).

From Fig. 4 it is evident that (1) 14 of 32 SMP transcripts are specifically expressed in the mantle, without detectable expression in the foot, (2) all mantle-specific SMP transcripts are more strongly expressed on the left side than the right, and (3) those 14 transcripts dominate the left side of Fig. 4. The 14 SMPs include those that are homologous to the three room-keeping proteins, ovipostatin (Ls-SMP150), matrilin-like (Ls-SMP-48), and cysteine-rich venom protein LIO1-like protein (Ls-SMP-108), three uncharacterized proteins (Ls-SMP-30, Ls-SMP-192, and Ls-SMP-63), and 8 novel proteins (Ls-SMP-39, Ls-SMP-61, Ls-SMP-4, Ls-SMP-60, Ls-SMP-70, Ls-SMP-59, Ls-SMP-62, and Ls-SMP-206) (Fig. 5). An ELH domain is contained in the novel protein Ls-SMP-4, two EFh domains are present in each of the novel proteins Ls-SMP-61 and Ls-SMP-62, and a CLECT domain and an H_lectin domain are found in the novel protein Ls-SMP-59 and the uncharacterized protein Ls-SMP-63 (Fig. 5). One or more low complexity regions are seen in Ls-SMP-150, Ls-SMP-60, Ls-SMP-63, and Ls-SMP-108 (Fig. 5). Amino acid composition analyses of low complexity regions indicate that the low complexity regions in Ls-SMP-60, Ls-SMP-63, Ls-SMP-108, and Ls-SMP-150 are rich in acidic residues (58.3%, 33.3%, 39.3%, and 28.6% acidic residues, respectively) (Supplementary Table S8). Signal peptides have been identified in most of those SMPs (10/14). Potential N-glycosylation (11/14), O-glycosylation (8/14), or phosphorylation (14/14) sites have also been inferred for those proteins (Supplementary Table S7). Although the actual functions of those SMPs must yet be confirmed by *in vivo* functional analysis, those 14 SMPs appear to be important in shell biomineralization, especially in suppressing shell precipitation, because they are specifically expressed in the mantle and they are more highly expressed on the left than the right side.

The other 18 SMP-encoding transcripts are expressed in both mantle and foot; thus, they are not specific to the mantle. However, since they show a significant difference in expression levels between the left and right sides of the mantle, they are probably important in shell formation. Of the 18 SMP-encoding transcripts, 11 are more highly expressed on the left than the right. They include a chitin-binding domain (ChtBD2)-containing protein (Ls-SMP-88), proteins homologous to two house-keeping proteins, alkaline phosphatase (Ls-SMP-35) and pancreatic triacylglycerol lipase-like protein (Ls-SMP-49), 5 SMPs homologous to the room-keeping protein, hemocyanin (Ls-SMP-170, Ls-SMP-171, Ls-SMP-164, Ls-SMP-165, and Ls-SMP-166), and 3 novel proteins (Ls-SMP19, Ls-SMP-2, and Ls-SMP-120). These three novel proteins all have one or more low-complexity regions, including one rich in acidic residues (42.9% of Asp: Ls-SMP-120) (Supplementary Table S8).

The remaining 7 SMPs, which are expressed more strongly on the right than the left, comprise three house-keeping proteins, the bromodomain-containing protein, DDB_G0280777-like protein (Ls-SMP-129) and two hephaestin-like proteins, which include Cu-oxidase domains (Ls-SMP-102 and Ls-SMP-105), one room-keeping neurogenic locus notch homolog protein (Ls-SMP-149), two uncharacterized proteins (Ls-SMP-43 and Ls-SMP-50), and one novel protein (Ls-SMP-203). These three novel and uncharacterized proteins (Ls-SMP-43, Ls-SMP-50, and Ls-SMP-203) have one or more low-complexity regions, and Ls-SMP-43 has a tyrosinase domain.

As discussed above, the 32 SMPs identified by left-right comparisons include a number of proteins that contain apparently important domains. In addition, the 207 SMPs identified in this study include 65 proteins (31.4%) that are homologous to known house-keeping proteins, but among the 32 left-right asymmetric SMPs, the number of proteins homologous to known house-keeping proteins is only 5 (15.6% of the 32 SMPs). Thus, the exercise of left-right comparisons appears to have successfully narrowed the list of potentially important SMPs.

In order to validate the above observations of asymmetric left-right expression of SMP genes in the mantle, levels of gene expression were also compared using quantitative PCR (qPCR). Overall, qPCR results did not show any trends significantly contradictory to those of transcriptome comparisons (Supplementary Fig. S3 and Supplementary Table S9). However, some individual biological replicates showed contradictory patterns, resulting in detection of only four SMPs (Ls-SMP-2, Ls-SMP-19, Ls-SMP-88, and Ls-SMP-149) that indicated statistically significant differences between left and right, with the former three (Ls-SMP-2, Ls-SMP-19, and Ls-SMP-88) being more highly expressed on the left than the right, and Ls-SMP-149 more highly expressed on the right than the left. Four other SMPs (Ls-SMP-48, Ls-SMP-59, Ls-SMP-129, and Ls-SMP-203) also indicated patterns of gene expression perfectly consonant with those of the transcriptome analysis; however, since they did not show statistically significant differences between left and right, we focused on the former four SMPs as the most important SMPs identified in our study.

## Discussion

Traditionally, prediction of functionally important SMPs identified by proteomic analysis has relied on sequence similarities, including the presence of domains conserved among SMPs and other proteins (e.g.^22,23,36^) and the abundance of SMPs contained in the shells^23^. These two approaches have been effective in identifying dozens of potentially important SMPs (see Supplementary Material for detailed discussion). However, since the *in vivo* functions of those conserved domains or known SMPs in biomineralization have yet to be clarified, identification of sequences homologous to those domains or proteins may lead nowhere. In addition, the mere abundance of SMPs may be an inadequate measure of importance.

Since shell matrix protein are secreted by mantle epithelial cells, genes with high expression levels in the mantle would result in high abundances of gene products contained in the shell, reflecting their involvement with shell formation. Contrary to this assumption, mean gene expression level of SMPs in the whole mantle is not correlated with the number of peptides in the shell (Supplementary Fig. S4a: r_s_ = 0.054, p = 0.78; Spearman’s rank correlation coefficient). The 4 proteins with the highest gene expression levels (FPKM: >2.0 × 10^4^) are rarely detected in the shell (protein abundance: <5.00). Highly expressed SMP genes with FPKM values greater than 2.0 × 10^4^ include those encoding a house-keeping protein homolog that lacks a signal peptide, such as Ls-SMP-173 (60S ribosomal protein P2) and Ls-SMP-175 (ATP-dependent RNA helicase DDX43) (Supplementary Table S4). Notably, these genes are not differentially expressed between left and right sides of the mantle. These results imply that these SMPs are accidentally entombed in the shell, merely because they are abundant in epithelial cells.

Instead of averaged gene expression levels, asymmetric gene expression patterns in the mantle regions may offer a better measure of the functional importance of SMPs. Because snail shells are laterally asymmetric, accretionary shell growth implies asymmetric expression of functional SMP genes. For example, SMPs that suppress shell precipitation should be more highly expressed in the mantle region at the inner side of the shell than in that corresponding to the outer edge of the shell. In dextral shells, the outer and inner sides correspond to the right and left sides of the mantle, respectively. In this study, the dextral pond snail, *L. stagnalis*, has been studied, and expression levels have been compared between the right and left sides of the mantle tissues in three individuals, in order to identify functionally important SMPs.

Both transcriptomic and qPCR analyses of gene expression levels of SMPs between the right and left sides of mantle tissues showed that among asymmetrically expressed SMPs, those that are more highly expressed on the left than the right are three time more abundant than those that are more highly expressed on the right than the left (Fig. 5). This observation was unexpected because we assumed that a dextrally coiled shell is produced by a greater shell precipitation on the right than the left side of the mantle, and that more shell precipitation-promoting SMPs would be expressed on the right than the left. Our results, however, suggest that a dextrally coiled shell is produced by inhibition of shell precipitation on the left. Inhibitory roles of SMPs have long been recognized^37,38^ and could be at work in production of coiled shells.

One of the three SMPs that showed higher expression on the left than the right, in both transcriptomic and qPCR analyses (Ls-SMP-88), showed a significant sequence similarity to Pif (Fig. 5), an SMP originally isolated from the pearl oyster *Pinctada fucata*^39^. It contains two chitin-binding domains (ChtBD2) and an extracellular domain (Laminin_G), as in Pif. However, it has no von Willebrand factor type A domain (VWA), which is involved in protein binding, and is always found in Pif^39–41^. There exists a Pif-like SMP, known as BMSP (Blue Mussel Shell Protein)^42^, originally isolated from the bivalve, *Mytilus galloprovincialis*. It has four VWA domains, a ChtBD2 domain, and a Laminin_G domain. Phylogenetic analysis of ChtBD2 and Laminin_G domain sequences indicated that Ls-SMP-88 is closer to Pif than to BMSP, suggesting that it originated as Pif, but lost the VWA domain subsequently (Supplementary Figs. S5 and S6; see Supplementary Material for detailed discussion). In pearl oysters, Pif binds aragonite crystals and promotes nacre formation^39^. Although functions of Ls-SMP-88 have yet to be clarified, one possibility is that the loss of the VWA domain led to loss of shell formation-promoting roles and acquisition of inhibitory roles instead.

Two other SMPs that indicated higher expression on the left than the right in both transcriptomic and qPCR analyses (Ls-SMP-2 and Ls-SMP-19) do not show any similarity to known proteins or domains. Except for the fact that Ls-SMP-2 and Ls-SMP19 have two and one low complexity region(s) (LCRs), respectively (Supplementary Table S8), they do not to show any apparent features characteristic of SMPs. Further studies are required to confirm their importance in biomineralization.

Ls-SMP-149 was the only SMP more highly expressed on the right than the left in both transcriptomic and qPCR analyses. It showed significant sequence similarity to the neurogenic locus Notch (NlN) homolog protein-like isoform X1 of the pond snail, *Biomphalaria grabrata* (Fig. 5). Although it is annotated as a homolog of NlN in *B. grabrata*, its overall domain composition and domain arrangements are entirely different from those of other typical NlN proteins in *B. grabrata*, *Crassostrea gigas*, *Drosophila melanogaster*, and *Homo sapiens* (Supplementary Fig. S7). Ls-SMP-149 has a single EGF domain (which exists in multiple copies in typical NlNs) and two Whey Acidic Protein (WAP) domains (that do not exist in NlN). Ls-SMP-149, therefore, is unlikely to function as a NlN. Since it has two WAP domains that exhibit an antiproteinase function^43^, it may act as a proteinase inhibitor, although its function in biomineralization needs to be verified. A WAP domain-containing SMP (isotig_7807) has been isolated from the land snail, *Cepaea nemoralis*^14^; however, isotig_7807 has only one WAP domain and no EGF domain. Thus, Ls-SMP-149 and isotig_7807 are not very similar. Phylogenetic analysis of the WAP domain sequences tends to support different origins (Supplementary Fig. S8).

In a previous shell proteome study of *L. stagnalis*, Herlitze *et al*. (2018) identified 46 shell-forming protein candidates and analysed their expression patterns in trochophore larvae and in the outer mantle lip of juveniles^32^. Of those 46 sequences, 30 have homologs (corresponding to 24 SMPs) in our SMP data, but we could not find an SMP in our data homologous to the remaining 16 sequences (Supplementary Table S10). Those 16 sequences comprise 9 that were found in our transcriptome data, but were not found in our proteome data (Case I), and 7 sequences that were not found in our data at all (Case II). Case I may have arisen due to the more stringent conditions, under which our proteome analyses were conducted. We analyzed only protein sequences that were identified by more than one unique peptide, while Herlitze *et al*. (2018) accepted those identified by only one peptide fragment. Case II can be explained by (1) the incompleteness of the transcriptomic data, (2) differences in methods of sequence assembly, and (3) genetic differences of the strains used. Between the homologous sequences of their 30 candidates and our 24 SMPs, only one SMP [Ls-SMP-61: *Lstag-sfc-*16 of Herlitze *et al*. (2018)] displayed an asymmetric gene expression pattern in the mantle in our transcriptome analysis. This SMP has two EFh domains, and it is more highly expressed on the left side of the mantle. However, this SMP gene did not show detectable signals in the expression analysis of Herlitze *et al*. (2018). Of their remaining 29 candidate sequences found among our sequences, 17 sequences are more highly expressed on the right side of the outer lip of juvenile mantle. However, corresponding SMP genes in our data did not show significant differences between left and right (Supplementary Table S10). These differences may be explained by the fact that the “left-right” direction of Herlitze *et al*. (2018) corresponds to the anterior-posterior direction in our study, as evidenced by the illustration for the ventral view of a juvenile in Fig. 3 of Herlitze *et al*. (2018). On the other hand, four sequences [*Lstag-sfc-1*, *Lstag-sfc-2a*, *Lstag-sfc-2b*, and *Lstag-sfc-3* of Herlitze *et al*. (2018)], which were not identified as SMPs in our study, showed a similar expression pattern in that are expressed more strongly on the right side of the mantle in our study, and are expressed on the right side of the asymmetric border of the shell field in trochophore larvae in Herlitze *et al*. (2018) (Supplementary Table S10). This commonality may reflect the fact that the left-right direction in trochophore larvae in Herlitze *et al*. (2018) corresponds to the left-right direction in our study.

## Conclusions

In this study, we preformed a comprehensive study of SMPs by combining transcriptomic analyses using NGS and proteomic analyses using mass spectrometry to identify 207 SMPs from the pond snail, *Lymnaea stagnalis*. We focused on the fact that rates of shell formation differ between the helical axis and the outer edges of snail shells, and we compared levels of gene expression, including those of 207 SMPs between the left and right sides of the mantle tissues. As a result, 32 SMPs that indicated significant differences between left and right have been identified. Quantitative PCR performed on those 32 SMP genes revealed that four SMPs were consistently asymmetric. Although expression levels are low, those four and the 28 other SMPs identified in this study are clearly first candidates for further functional characterizations for the following reasons. In addition to their left-right gene expression asymmetry, they showed (1) sequence similarities to known and likely important SMPs, such as Pif, (2) possession of potentially important functional domains, such as EFh, CLECT, and to chitin binding, and (3) mantle-specific expression of the genes when they showed a large change of gene expression between left and right (see Supplementary Material for detailed discussion).

Snails are well suited to the study of not only biomineralization, but also development of asymmetric morphologies due to the intrinsic nature exploited in this study. *L. stagnalis* will be a good model organism with a relevance to a wide range of research fields, such as neurophysiology, embryology, environmental toxicology, and biomineralization^25–28,32^. In the future, it will be necessary to perform more detailed spatial and temporal gene expression analyses, and *in vivo* functional analysis of SMPs using a genome editing method such as CRISPR/Cas9, for the candidates identified in this study^44^.

## Material and Methods

### Animals and protocol for extracting RNA

Pond snails, *Lymnaea stagnalis* (strain GSS7-1), originally collected in Neustadt, Donau, Germany, were reared in deionized and calcium carbonate-saturated tap water at about 23 ± 1 °C (Fig. 1a). Mantle tissues were excised from three individuals, each having been separated into left and right portions using scissors (Fig. 1b). Foot tissues were excised from another individual using scissors. Total RNA was extracted from each sample using ISOGEN (Nippon Gene, Tokyo, Japan), following the protocol of Isowa *et al*.^22^.

### Transcriptome analyses

Transcriptomic analyses of mantle and foot RNA were carried out using HiSeq. 2500 (Illumina, California, USA) and MiSeq (Illumina, California, USA) sequencers, respectively. Six mantle libraries were prepared from left and right mantle tissues for each of three biological replicates. The library for foot tissues was prepared from one individual. Low-quality regions were removed from the FASTQ files before assembling the nucleotide sequences in FASTA format using Trinity (v2.5.1)^45,46^ and the DDBJ Read Annotation Pipeline (http://p.ddbj.nig.ac.jp/pipeline/; last accessed April 5, 2018)^47,48^ with the default setting of k-mer (= 25). Gene models were predicted using TransDecoder (v4.1.0)^46^, and redundant sequences were removed with CD-HIT (v4.6.1)^49,50^. Sequence assembly, gene set, and transcriptome completeness of the FASTA file were verified with BUSCO (v3.3)^33,34^ using the metazoan data set.

### Protocol for extracting shell proteins

Shell proteins were extracted from 20 g of shells from about 60 individuals. First, shells were treated with sodium hypochlorite to remove any surface contaminants and then washed with ultrapure water to remove sodium hypochlorite. Second, washed shells were dissolved in an aqueous solution of 460 mL of 0.5 M ethylenediaminetetraacetic acid (EDTA), the pH adjusted to 8.0 using NaOH. After 48 h of incubation at 4 °C, the supernatant was decanted, and EDTA was removed with Amicon ultra-15 centrifugal filter units (Merck Millipore, Burlington, USA). This EDTA-soluble protein mixture is denominated the soluble fraction. The precipitate was dissolved in an aqueous solution containing 7 M urea, 2 M thiourea, 3% 3-[(3-cholamidopropyl)dimetilaminio] propanesulfonate (CHAPS), and 1% Triton X-100), and after 24 h of incubation at 4 °C, the preparation was centrifuged at 20,000 g, at 4 °C for 1 h. The supernatant was decanted, and urea and other salts were removed with Amicon ultra-15 centrifugal filter units (Merck Millipore, Burlington, USA). Th protein mixture solubilized by this procedure is referred to as the insoluble fraction. The amounts of soluble and insoluble fractions, in aqueous and CHAPS solutions, respectively, were quantified using a Qubit Fluorometer (Thermo Fisher Scientific, Massachusetts, USA).

### Proteomic analysis

Each of the protein samples extracted using the above protocol was dissolved in 200 μL of 0.1 M Tris-HCl buffer (pH 8.5). After adding 600 μL of methanol and 150 μL of chloroform, the sample was centrifuged (13,000 g, 4 °C, 10 min.). After removing the supernatant, 500 μL of methanol was added to the sample and centrifuged again (20,400 g, 4 °C, 10 min.). The supernatant was then removed, and the sample was dried by Speed Vac (EYELA, Tokyo, Japan). The dried pellet was dissolved in an aqueous solution containing 8 M urea, 0.1 M Tris-HCl (pH 8.5) and 0.1 M DTT, and was incubated for 1 h at 37 °C. A volume of 0.5 μL 208 mM iodoacetamide was added to the sample, which was incubated for 1 h at room temperature in the dark. After adding 0.1 M Tris-HCl and ultrapure water, it was treated with trypsin (Promega, Wisconsin, USA) in a 20-fold excess over sample protein and incubated overnight at 37 °C. Tryptic peptides were analyzed with an LTQ Orbitrap mass spectrometer (Thermo Fisher Scientific, Waltham, MA, USA) coupled with a DiNa nanoLC system (KYA Technologies, Tokyo, Japan). Precursor ions were detected over a range of 400–1,500 m/z, and the top four high-intensity ions were selected for MS/MS analyses in data-dependent mode. Acquired MS/MS spectra were subjected to a database search against the protein sequence database translated from transcriptome data from the mantle tissues of *L. stagnalis* with the SEQUEST program using Proteome Discoverer software version 1.2 (Thermo Fisher Scientific, Waltham, MA, USA). Parameters were set as follows: the charge state of the precursor ions: automatically recognized; the mass range of tryptic peptides: 800–4,500; mass tolerances for precursor ions: 10 ppm; mass tolerances for fragment ions: 1 Da. Up to two missed cleavages and modifications of carbamidomethylation (+57.021) of cysteine and oxidation (+15.995) of methionine were considered for calculation of theoretical masses. False discovery rates (FDRs) were calculated based on a decoy database using Proteome Discoverer software. A list of identified peptides that include a false discovery rate <1% was obtained after filtering low-confidence identification. Protein sequences identified by more than one unique peptide were retained.

### Sequence annotation

We performed BLAST searches against GenBank at NCBI (National Center for Biotechnology Information; http://blast.ncbi.nlm.nih.gov; last accessed April 10, 2019)^51,52^ using the non-redundant database (all organisms) with an e-value cutoff of 10^−10^. Conserved domains were searched using Simple Modular Architecture Research Tool (SMART; v8.0; http://smart.embl-heidelberg.de; last accessed April 10, 2019)^53,54^ provided by EMBL (European Molecular Biology Laboratory), including optional searches for outlier homologs and homologs of known structure, Pfam domains, and signal peptides. Gene Ontology (GO) terms were assigned using Blast2GO software (version 4.1; https://www.blast2go.com)^55^ for the categories cellular component, biological process, and molecular function. For each category, GO terms are assigned at 4 hierarchical levels, and term frequencies at three levels (levels 2–4) are visualized and considered in this study. Theoretical isoelectric points (pI) and theoretical molecular masses of identified proteins were estimated using UniProt (http://www.uniprot.org/; last accessed August 20, 2018)^56^. Potential O-glycosylation, N-glycosylation, and phosphorylation sites were predicted using the servers of DTU Bioinformatics (https://services.healthtech.dtu.dk/; last accessed September 10, 2018).

### Gene expression analysis

Analyses of gene expression levels were performed with Bowtie2 (v2.3.4.3; http://bowtie-bio.sourceforge.net/bowtie2/index.shtml; last accessed November 21, 2018)^57^ and eXpress (v1.5.0; https://pachterlab.github.io/eXpress/; last accessed November 21, 2018)^58^ using the DDBJ Read Annotation Pipeline. Differential gene expression was evaluated using edgeR (v3.18.1; https://bioconductor.org/packages/release/bioc/html/edgeR.html; last accessed November 25, 2018) in the Bioconductor package of the R project (https://www.r-project.org/; last accessed November 25, 2018)^59,60^. Statistical analysis of differential expression between right and left mantle tissues was performed based on the nonparametric Fisher’s exact test, using edgeR. Statistical significance levels were calculated and corrected using the FDR (false discovery rate) to avoid false positives arising from multiple tests. The q value was set at 0.05.

### Quantitative PCR (qPCR)

Total RNA was extracted from left and right mantle tissues of 5 biological replicates of *L. stagnalis* using ISOGEN (Nippon Gene, Tokyo, Japan) following the protocol of Isowa *et al*.^22^. Total RNA was also extracted from the foot tissue of one specimen of *L. stagnalis* using the method above. Total RNA samples were treated with DNase I using RQ1 RNase-Free DNase (Promega, Madison, USA). To confirm the effectiveness of the DNase I treatment, PCR was performed with primer sets for EF1ɑ of *L. stagnalis* with total RNA samples as a template following the protocol of Young *et al*.^61^.

Complementary DNA was prepared from 1 μg of each total RNA sample using iScript RT Supermix for RT-qPCR (Bio-Rad, Hercules, USA), which contained a mixture of oligo(dT) and random primers. Reverse Transcription PCR was performed using a GeneAmp PCR system 9700 (Applied Biosystems, Foster City, USA) following the protocol of Young *et al*.^61^.

Quantitative PCR was performed using a StepOne Real-time PCR system (Applied Biosystems, Foster City, USA). Amplification of SMP genes was detected with SyBR Green dye. Reactions contained 2 μL of cDNA sample (25 ng/μL) with 5 μL of SsoAdvanced Universal SyBR Green Mix (Bio-Rad, Hercules, USA), 250 nM forward and reverse primer concentrations, and were brought to 10 μL with DNase- and RNase-free water. Two technical replicates were performed for each of the 5 biological replicates. The custom qPCR method consisted of 95 °C for 30 s; 40 cycles of 95 °C for 15 s, 60 °C for 30 s. Gene expression was analysed with the comparative C_T_ method. Foot tissues were selected as references and *Lst-EF1ɑ* (EF1ɑ gene of *L. stagnalis*) designed by Young *et al*.^61^ was selected as an endogenous control. Primers for the 32 “asymmetric” SMP genes were designed using Primer3Plus (https://primer3plus.com/cgi-bin/dev/primer3plus.cgi; last accessed November 11, 2019) to have a length of 19–23 nucleotides, a melting temperature between 57–62 °C, a GC content from 40–60%, and a product size range of 70–150 bp (Supplementary Table S11). As a comparison, we also analysed three “symmetric” SMP genes, which indicated the highest FPKM values in the transcriptome data, by qPCR. Primers for those three SMP genes were also designed using the same method.

## Supplementary information

Supplementary Information.

Dataset 1.

Dataset 2.

Dataset 3.

Dataset 4.

Dataset 5.

Dataset 6.

Dataset 7.

Dataset 8.

Dataset 9.

Dataset 10.

Dataset 11.

Dataset 12.

Dataset 13.

## Data Availability

Transcriptome data from this study are available in the DDBJ Sequence Read Archive (DRA) under accession numbers DRA005517 and DRA006373. All other datasets used in this study are available from the corresponding authors on request.
