# Supplementary material for: Functional shell matrix proteins tentatively identified by asymmetric snail shell morphology
Source: Sci Rep. 2020 Jun 17;10:9768. doi: 10.1038/s41598-020-66021-w (PMC7299971; doi:10.1038/s41598-020-66021-w)
Supplement: Supplementary file 1 — Supplementary Information. [file 41598_2020_66021_MOESM1_ESM.pdf]

Supplementary Information of

Functional shell matrix proteins tentatively identified by asymmetric snail shell morphology

Akito Ishikawa[1], Keisuke Shimizu[2], Yukinobu Isowa[3], Takeshi Takeuchi[4], Ran Zhao[1], Keiji Kito[5], Manabu Fujie[6], Noriyuki Satoh[4] and Kazuyoshi Endo[1]

[1]Department of Earth and Planetary Science, Graduate School of Science, The University of Tokyo, 7-3-1 Hongo, Bunkyo, Tokyo 113-0033, Japan. [2]Department of Applied Biological Chemistry, Graduate School of Agricultural and Life Sciences, The University of Tokyo, 1-1-1 Yayoi, Bunkyo, Tokyo, 113-8657, Japan. [3]Sugashima Marine Biological Laboratory, Graduate School of Science, Nagoya University, 429-63 Sugashima, Toba 517-0004, Japan. [4]Marine Genomics Unit, Okinawa Institute of Science and Technology Graduate University, 1919-1 Tancha, Onna-son, Kunigami-gun, Okinawa 904-0495, Japan. [5]Department of Life Sciences, School of Agriculture, Meiji University, 1-1-1 Higashimita, Tama, Kawasaki, Kanagawa 214-8571, Japan. [6]DNA Sequencing Section, Okinawa Institute of Science and Technology Graduate University, 1919-1 Tancha, Onna-son, Kunigami-gun, Okinawa 904-0495, Japan. Correspondance and requests for materials should be addressed to A.I. (email: aishikawa.0218@gmail.com) or K.E. (email: endo@eps.s.u-tokyo.ac.jp).

Supplementary Material

Supplementary Results

Expression levels of SMP genes in mantle tissue

FPKMs for each of the 207 SMPs were calculated from transcriptomic data. Supplementary Fig. S9 indicates the 10% of sequences with the highest FPKMs among the 207 SMPs (Supplementary Fig. S9 and Supplementary Table S4). The sequence with the highest FPKM is Ls-SMP-126, annotated as adductor muscle actin by BLAST. Other sequences with top 10% expression levels are dominated by apparent house-keeping genes, including 60S acidic ribosomal P2 (Ls-SMP-173), actin (Ls-SMP-127), L-amino-acid oxidase (Ls-SMP-33), arginase-1-like isoform X2 (Ls-SMP-114), ATP-dependent RNA helicase DDX43 (Ls-SMP-175), voltage-dependent anion-selective channel protein 2-like isoform X4 (Ls-SMP-12), retrograde protein of 51 kDa (Ls-SMP-77), 14-3-3 protein epsilon (Ls-SMP-17), and YGSC-1 (Ls-SMP-176). Some proteins that had already been identified as SMPs, such as formin-like protein 2 isoform X3 (Ls-SMP-53), matrilin (Ls-SMP-90), extensin-like isoform X1 (Ls-SMP-52), and dermatopontin 1 (Ls-SMP-195) also exhibited high expression levels, and were included in the top 10% of sequences based on FPKMs. The remaining sequences in Supplementary Fig. S9 include hemocyanin alpha D-subunit (Ls-SMP-170), a “room-keeping” gene, and an unidentified protein (Ls-SMP-172) or uncharacterized proteins including LOC106071610 (Ls-SMP-51) and LOC106053304 (Ls-SMP-85) (Supplementary Fig. S9 and Supplementary Table S4).

Abundances of SMPs in the shell

Protein abundance, or the amount of a protein in the shell matrix, was estimated from proteomic data. Supplementary Fig. S10 shows the most abundant 10% of protein sequences among the 207 SMPs (Supplementary Fig. S10 and Supplementary Table S4). The most abundant protein, Ls-SMP-203, could not be identified by BLAST or conserved domain search. Indeed, more than half of the most abundant 10% of shell proteins were either unidentified (Ls-SMP-45, Ls-SMP-4, Ls-SMP-120, Ls-SMP-161, Ls-SMP-96, Ls-SMP-7, Ls-SMP-57, Ls-SMP-5, Ls-SMP-10 and Ls-SMP-144) or uncharacterized [coiled-coil domain-containing 1-like isoform X2 (Ls-SMP-198), LOC106053304 (Ls-SMP-85), LOC106070421 (Ls-SMP-200), LOC106069873 (Ls-SMP-197), and LOC106073248 (Ls-SMP-202)] proteins. The remaining proteins had already been identified as either SMPs [formin-like protein 2 isoform X3 (Ls-SMP-53), extensin-like isoform X1 (Ls-SMP-52), and galaxin (Ls-SMP-97)], or an otolith matrix protein [starmaker-like isoform X1 (Ls-SMP-199)].

Gene ontology analysis

Gene ontologies (GO) of the mantle and SMP-encoding transcriptomes were analyzed and visualized with Blast2GO. Annotated genes were classified into three different categories, "cellular component", "biological process", and "molecular function", with three different levels for each category (Supplementary Figs. S11 – S16). In the categories, "cellular component" and "biological process", no notable differences were observed between the mantle and SMP transcriptomes, except that at level 2 of "cellular component", the term "extracellular region" is enriched in SMPs compared to the mantle (Supplementary Figs. S11, S12, S14, and S15). On the other hand, some differences were seen in the category "molecular function." Especially at level 3, the GO terms "hydrolase activity", "protein binding", "oxidoreductase activity", and "carbohydrate derivative-binding" are enriched in SMPs (Supplementary Figs. S13 and S16). At level 4, the GO terms "cation binding" and "anion binding" are enriched in SMPs compared to the mantle (Supplementary Figs. S13 and S16).

Phylogenetic analyses of some conserved domains

Pif proteins in general and the Pif-like protein (Ls-SMP-88) of *L. stagnalis* have two ChtBD2 domains arranged in tandem. Phylogenetic analyses of those ChtBD2 domains indicated that each forms a cluster (with bootstrap probabilities for the upstream and downstream domains being 64% and 68%, respectively), suggesting that the last common ancestor of bivalves and gastropods already had those two ChtBD2 domains in tandem (Supplementary Fig. S6a). Phylogenetic analyses also indicated that the Laminin_G domain sequence of the Pif-like SMP of *L. stagnalis* forms a cluster with sequences of typical Pif SMPs of other molluscs, rather than with Laminin_G sequences of BMSP (blue mussel shell protein) (Supplementary Fig. S6b).

Phylogenetic analyses of tyrosinase domains clearly discriminated three classes of TDC-SMPs: lophotrochozoan tyrosinase, vertebrate tyrosinase, and hemocyanin-tyrosinase (Supplementary Fig. S17). Thus, TDC-SMPs of *L. stagnalis* may be classified into two groups of different origin, i.e., tyrosinase SMP and hemocyanin-tyrosinase, with the latter having greatly expanded in *L. stagnalis* (Supplementary Fig. S17).

SDS-PAGE analysis

Matrix proteins were extracted from shells of *Lymnaea stagnalis*, which yielded 0.53 μg soluble and 1.39 μg insoluble fractions per g of shell. Extracted proteins separated by SDS-PAGE revealed 3 major (10 kDa, 20 kDa, and 22 kDa) and 2 minor (28 kDa and 220 kDa) bands for the soluble fraction and one major (20 kDa) and 3 minor (28 kDa, 32 kDa, and 220 kDa) bands for the insoluble fraction, when stained with Coomassie Brilliant Blue (Supplementary Fig. S18). As shown later, sizes of those proteins do not match those

of proteins with the highest numbers of hits in the proteomic analysis, suggesting that the major proteins seen in SDS-PAGE are products of post-translational modifications such as cleavage, glycosylation, and phosphorylation.

Supplementary Discussion

Depth and quality of the data

Integrated transcriptomic and proteomic analysis allowed us to obtain more complete sequences of SMPs than most previous studies. Previous proteomic analyses of molluscan shell matrices, including those of bivalves and gastropods, have often been based on Expressed Sequence Tag (EST) libraries. Mass spectrometric analyses coupled with EST libraries have identified 14 SMPs from *Haliotis asinina*[1], 8 from *Crassostrea gigas*[2], 9 from *Mytilus* spp.[3], 41 from *Pinctada margaritifera* and 43 from *Pinctada maxima*[4]. On the other hand, combined mass spectrometric and transcriptomic analysis identified many more SMPs, i.e., 79 SMPs from the bivalve *Mytilus coruscus*[5], 48 from the gastropod *L. stagnalis*[6], 243 from the brachiopod, *Magellania venosa*[7], and and 40 SMPs from another brachiopod, *Laqueus rubells*[8], respectively. In this study, we identified 207 SMPs from the pond snail, *L. stagnalis*, using the latter approach (Supplementary Table S12). These results indicate that mass spectroscopic analysis combined with transcriptomic analysis, rather than EST analysis, yields more comprehensive repertoires of SMPs.

The N50 value of the present transcriptome was 2,828 bp for 337,195 contigs. These scores are higher than those in transcriptomic analyses of other molluscs, such as *Mytilus edulis*[9] and *M. coruscus*[5], and brachiopods *Magellania venosa*[7] and *Laqueus rubellus*[8], indicating that our transcriptomic data have sufficient read depth and reasonable assembly (Supplementary Table S12).

On the other hand, the percentage of MS/MS spectra that matched the peptides in the protein sequences translated from transcriptomic data was only ~5% of the total MS/MS spectra acquired. This score is lower than those in other mass spectrometric proteomic analyses of model organisms whose genomes are completely sequenced (e.g. *Saccharomyces cerevisiae*, 40-50%). Thus there could be many more proteins in the shell matrix than were identified in this study. Only tryptic digests were analyzed in this study; therefore, proteins that are depauperate in lysine and arginine, such as highly acidic proteins, will not be readily identified with this approach[10].

Inferring important SMPs based on traditional similarity-based approaches

The 15 common domains identified in this study can be classified into different categories according to their known functions. Eight domains (EGF, FN3, VWA, WAP, SCP, DERM, Ependymin and CCP) are categorized as extracellular matrix domains. EGF (Epidermal

Growth Factor) (IPR000742) domain is a short peptide with a distinctive six-cysteine motif. The main structure involves a two-stranded beta-sheet followed by a loop and a C-terminal short, two-stranded sheet. Both calcium-binding EGF repeats and non-calcium-binding EGF repeats are known[11]. FN3 (Fibronectin type 3) (IPR003961) domain is one of the three types of internal repeat in the plasma protein, fibronectin. Fibronectin is involved in cell adhesion, cell morphology, thrombosis, cell migration, and embryonic differentiation[12]. VWA (von Willebrand factor type A) (IPR002035) domains in extracellular eukaryotic proteins mediate adhesion via metal ion-dependent adhesion sites, and von Willebrand factor is a large multimeric glycoprotein found in blood plasma[13]. WAP (Whey Acidic Protein) (IPR008197) domain constitutes the four-disulfide core of WAP. A number of proteins that have WAP domains have been shown to exhibit antiproteinase activity[14]. SCP (sperm-coating glycoprotein) (IPR014044) is also known as CAP (cysteine-rich secretory proteins, antigen 5, and pathogenesis-related 1 protein) domain, and is found in a wide range of organisms, including prokaryotes and eukaryotes, where it may function as an endopeptidase[15]. Thus, SCP domain can also be categorized as an enzyme domain. DERM (dermatopontin) (PF14704) is known as a low-molecular-weight protein in the extracellular matrix that has been reported to mediate cell adhesion by cell surface integrin binding[16,17]. Ependymin (IPR001299) is known as a secretory and calcium-binding meningeal glycoprotein found in the cerebrospinal fluid of teleost fish and has a bound form of glycoprotein associated with the extracellular matrix, including collagen fibrils[18]. CCP (IPR000436) domain is also known as Sushi domain. It has a beta-sandwich structure, and is found in selectin, which can bind to C-type lectins (CLECT)[19].

Two domains (CLECT, and ChtBD2) are categorized as polysaccharide interaction domains. CLECT (C-type lectin) (IPR001304) is a Ca^{2+} dependent carbohydrate-binding domain, and has functions in cell adhesion, immune response to pathogens, and apoptosis[19,20]. ChtBD2 (chitin-binding 2) (IPR002557), containing six conserved cysteine residues, is a chitin-binding domain, and is also known as an extracellular domain[21].

Two domains (EFh and Cu-oxidase) are categorized as a cation-interaction domain. EFh (EF-hand) (IPR002048) has a calcium-binding motif that is shared by many calcium-binding proteins[22]. Cu-oxidase (multicopper oxidase) (IPR001117) oxidizes substrate molecules by accepting electrons at a mononuclear copper center and transferring them to a trinuclear center[23]. Cu-oxidase is also categorized as an enzyme domain.

Three domains (Tyrosinase, An_peroxidase and Glyco_hydro_20) are categorized as enzyme domains. Tyrosinase (IPR002227) is an oxidase that controls production of melanin and other pigments. A tyrosinase domain has three histidine residues for interaction with copper atoms that are shared by some hemocyanins, which are copper-containing oxygen carriers from the hemolymph of many molluscs and arthropods[24].

An_peroxidase (IPR002007) is known as animal heme-dependent peroxidase, the heme-containing enzyme that uses hydrogen peroxide as an electron acceptor to catalyze various oxidative reactions[25–27]. Glyco_hydro_20 (glycoside hydrolase family 20) (IPR015883) is a widespread group of enzymes that hydrolyze the glycosidic bond between two or more carbohydrate moieties[28]. In *L. stagnalis*, SMPs containing DERM, An_peroxidase, CLECT, CCP, or VWA domains have been identified in previous studies[6,29].

Phylogenetic analyses of some conserved domains

Ls-SMP-88 has two chitin-binding-domains (ChtBD2) in tandem followed by a Laminin_G domain, as in typical Pif SMP (Supplementary Fig. S5)[30–32]. But unlike Pif, it lacks the VWA domain. Instead it contains 4 low-complexity regions (Supplementary Table S8). Thus, it is not a typical Pif, and is annotated here as Pif-like. Phylogenetic analyses of ChtBD2 indicated that the second ChtBD2 domain sequences of *Crassostrea gigas*, *Pinctada fucata*, *Lottia gigantea*, and *Lymnaea stagnalis* form a cluster (with bootstrap support of 68%) (Supplementary Fig. S6a). Phylogenetic analyses of Laminin_G domain sequences indicate that Ls-SMP-88 is closer to Pif of *C. gigas*, *P. fucata*, and *L. gigantea* than to BMSP of *L. gigantea* and *Mytilus galloprovincialis* (Supplementary Fig. S6b). Those observations suggest that the Pif-like protein of *L. stagnalis* originated from a Pif protein by substituting the VWA domain with low-complexity regions.

Tyrosinase domain-containing SMPs dominate the SMPs with an asymmetric gene expression pattern in the mantle, representing 6 of the 32 asymmetric SMPs (Ls-SMP-43, Ls-SMP-164, Ls-SMP-165, Ls-SMP-166, Ls-SMP-170, and Ls-SMP-171). Phylogenetic analyses demonstrated that those proteins comprise two distinct classes, tyrosinase (Ls-SMP-43) and hemocyanin (Ls-SMP-164, Ls-SMP-165, Ls-SMP-166, Ls-SMP-170, and Ls-SMP-171) (Supplementary Fig. S17).

Inferring important SMPs based on traditional abundance-based approaches

Other traditional approaches for prediction of important SMPs involve quantification of abundances of SMPs in the shells. When we look at amounts of SMPs, quantified for each protein by the number of times constituent peptides appeared in the spectrograms standardized by the length of the protein, the highest abundance was observed for Ls-SMP-203, which accounted for 14.7% of all 207 SMPs, and was annotated as a novel protein, followed by Ls-SMP-53 and Ls-SMP-52, which accounted for 6.0% and 4.5% of the total, respectively, and which were annotated as a formin-like protein and an extensin-like protein. All were represented by complete sequences in both the soluble and insoluble fractions. A common feature of these three proteins is that they contain at

least one low-complexity region. Ls-SMP-203 [theoretical molecular mass (Mm) = 20094.3; theoretical isoelectric point (pI) = 8.04] has a signal peptide, a potential O-glycosylation site, and 16 potential phosphorylation sites. Ls-SMP-53 (Mm = 19811.7; pI = 7.01) has no signal peptide with 11 potential phosphorylation sites. Ls-SMP-52 (Mm = 24230.1; pI = 8.89) has no signal peptide, a potential O-glycosylation site, and 13 potential phosphorylation sites (Supplementary Table S4).

The results of SDS-PAGE analysis revealed three major bands (10 kDa, 20 kDa, and 22 kDa) for the soluble fraction and one major band (20 kDa) for the insoluble fraction (Supplementary Fig. S18). Since all three of the highest abundance SMPs (Ls-SMP-203, Ls-SMP-53, and Ls-SMP-52) have molecular masses of about 20 kDa and are represented in both the soluble and insoluble fractions, it appears likely that they correspond to the 20 kDa bands seen in SDS-PAGE gels in the soluble and insoluble fractions. Those proteins may be important in shell formation by virtue of their high abundance, but details should be experimentally studied.

Inference of important SMPs based on traditional sequence-based approaches

In order to identify functionally important SMPs based on sequence similarities, the 207 SMPs identified in this study were first searched against GenBank using BLAST, and a total of 165 proteins showed similarity to known proteins. Of the 165 proteins, 70 were categorized as house-keeping proteins, 40 as room-keeping, 13 as SMPs, and 49 as uncharacterized proteins (Supplementary Table S4). Seven proteins were categorized as both room-keeping proteins and SMPs. This indicates that many SMPs identified in this study are house-keeping proteins that are probably irrelevant to shell formation. Some of the SMPs categorized as room-keeping proteins are also known as SMPs in other taxa or as an otolith matrix protein (Ls-SMP-199; starmaker-like), suggesting that those proteins could well be important in shell formation.

In order to further identify possibly important SMPs based on sequence similarities, searches for domains conserved among molluscan SMPs have been carried out. Out of the 261 domains identified in the 207 SMPs of *L. stagnalis* using SMART and InterProScan 5, fifteen domains are shared with at least one of the two other molluscs compared, the limpet, *Lottia gigantea*[33] and the oyster, *Crassostrea gigas*[34,35]. They include 8 extracellular matrix, two polysaccharide interaction, two cation interaction, and three enzyme domains (Supplementary Fig. S2). Of these 15 domains, 10 domains [5 extracellular matrix (CCP, EGF, FN3, VWA, and WAP), two polysaccharide interaction (CLECT and Cht_BD2), two enzyme (Tyrosinase and An_Peroxidase), and one cation interaction (EFh) domains] are shared among all three species. Two domains [one enzyme (Glyco_hydro_20) and one cation interaction (Cu-oxidase) domains] are shared only by *L. stagnalis* and *C. gigas*, and three domains [three extracellular matrix

(Ependymin, DERM, and SCP) domains] by only *L. stagnalis* and *L. gigantea* (Supplementary Fig. S2). SMPs containing one of those domains may be functionally important[7,36].

Of SMPs containing at least one of those 15 domains, 9 SMPs are specific to the mantle transcriptome, showing no detectable expression in the foot (Supplementary Table S4). They include Ls-SMP-23 (DERM), Ls-SMP-24 (DERM), Ls-SMP-48 (VWA), Ls-SMP-59 (CLECT), Ls-SMP-61 (EFh), Ls-SMP-62 (EFh), Ls-SMP-81 (CLECT), Ls-SMP-82 (CLECT), and Ls-SMP-186 (EFh) (shared domain names are indicated in parentheses). Ls-SMP-23 and Ls-SMP-24 are dermatopontin homologs that are inferred to be splicing variants (Supplementary Fig. S19). Sarashina et al. (2006) reported three dermatopontin genes from *L. stagnalis*, but considered only one as an SMP[29]. In this study, all of these dermatopontin homologs were identified as SMPs, but their transcripts were also expressed in the foot. Ls-SMP-23 and Ls-SMP-24 are newly identified in this study as SMPs, of which transcripts are specific to the mantle. Ls-SMP-81 was identified as a perlucin-like protein, and Ls-SMP-82 is a likely splicing variant of Ls-SMP-81. Perlucin is an SMP that may promote nucleation and/or growth of calcium carbonate crystals with an ability to bind D-galactose and D-mannose/D-glucose. It has been identified in many other molluscs[20,37,38]; thus, it appears likely to be actively involved in shell formation. Ls-SMP-61, Ls-SMP-62, and Ls-SMP-186 have EFh domains, which could mediate calcium ion binding during biomineralization. Ls-SMP-48 has a VWA domain, and is been identified as matrilin-like by BLAST. Matrilin is known as an extracellular protein.

Our signal peptide search indicated that of the 147 SMPs with complete sequences, only 99 are predicted to have signal peptides. In total, 115 SMPs were predicted to have signal peptides. That those SMPs have signal peptides is consistent with the fact that SMPs are secretory proteins. On the other hand, for the remaining 92 of the 207 SMPs, or 48 of the 147 SMPs with a complete sequence, signal peptides were not predicted. SMPs without signal peptides may have originated from either extracellular regions of proteins with transmembrane regions or from contaminating cells[10]. However, since no transmembrane regions have been identified in SMPs with complete sequences, contaminating cells, such as hemocytes, are the more likely source. The fact that SMPs without signal peptides are dominated by house-keeping proteins, such as 60S ribosomal proteins and histone (Supplementary Table S4), tends to support this interpretation. Thus, those SMPs may have accidently leaked from cells such as dying or randomly trapped cell remnants and may not be important in shell formation.

Our domain searches revealed that SMPs categorized as novel or uncharacterized proteins by BLAST, often contain one or more low-complexity regions (LCRs), with 14, 13, 6, 24, and 24 LCR-containing SMPs having been identified among 70 house-keeping, 33 room-keeping, 13 known SMPs, 49 uncharacterized, and 42 novel proteins

(Supplementary Table S4). LCRs are often present in SMPs and are considered important in shell precipitation[10,39]. Therefore, novel or uncharacterized SMPs that contain LCRs may also be important in shell formation.

In order to further characterize SMP-encoding genes, based on sequence comparisons, we compared gene ontology (GO) between SMP-encoding genes and mantle transcripts. The results indicated that in the category "molecular function", notable differences were observed in hydrolase activity, protein binding, oxidoreductase activity, and carbohydrate derivative-binding at level 3, and in cation binding and anion binding at level 4. Those terms are enriched in SMP-encoding genes (Supplementary Figs. S13 and S16). For the other two categories "cellular component" and "biological process", notable differences were not observed between the mantle and SMP transcriptomes, except for the term "extracellular region" at level 2 of "cellular component", a term enriched in SMP transcripts (Supplementary Figs. S11, S12, S14, and S15). Proteins that indicated enrichment in GO terms typically seen in SMP-encoding genes could be important in shell formation. Enrichment of the term "extracellular region" is concordant with the fact that SMPs are secretory proteins.

Marin et al. (2016) classified the sources of organic matrices in animal calcium carbonate skeletons into three categories: (1) secretome, (2) cleaved extracellular domains of transmembrane proteins, and (3) cellular contaminants. They distinguished SMPs originating from (1) and (2) (skeletome) from those in (3) (entrapped contaminants)[10]. A secretome, by definition, is comprised of proteins with a signal peptide. Of the 32 left-right asymmetric SMPs, 18 SMPs have a signal peptide, and are thought to have originated from the secretome of this species. On the other hand, 14 SMPs did not show potential signal peptides, although 11 of those 14 SMPs are represented by 5' partial or internal sequences, and could have signal peptides. Thus, three SMPs (Ls-SMP-39, Ls-SMP-50, and Ls-SMP-170) remain as complete sequences without signal peptides. They cannot be identified as belonging to the above category (2) because they do not have transmembrane regions in domain searches using InterProScan5, SMART, and Blast2GO. Ls-SMP-39 is a novel protein, showing a high value of logFC and the lowest p value in the test of the difference in gene expression levels between the left and right (Fig. 5). Ls-SMP-50 is homologous to an uncharacterized protein identified from *Biomphalaria glabrata*, and has two low complexity regions, but has no known functional domains. It is more strongly expressed in the right mantle than in the left (Fig. 5). Ls-SMP-170 has been identified as a hemocyanin homolog, and has a low-complexity region, although its function in shell formation is unknown (Fig. 5). Incidentally, Marin et al. (2016) considered only coral skeletal proteins as included in this category having a transmembrane protein extracellular domain. Isowa et al. (2015) and Jackson et al. (2015) reported SMPs containing one or more transmembrane domains from the

brachiopods *Laqueus rubellus* and *Magellania venosa*, respectively, and they may represent other SMPs belonging to this category[7,8]. According to the classification of Marin et al. (2016), therefore, the three proteins without signal peptides may be regarded as cellular contaminants. Although we cannot completely exclude this possibility, since they showed asymmetric gene expression between left and right, they may not be just cellular contaminants. One possibility is that they function to maintain the asymmetric nature of the mantle in regard to growth and metabolism, indirectly contributing to asymmetric shell formation.

Supplementary Methods

Sodium dodecyl sulfate polyacrylamide gel electrophoresis (SDS-PAGE)

Soluble and insoluble fractions were separated by SDS-PAGE, in a 10% polyacrylamide slab gel. After electrophoresis, the gel was stained with Coomassie Brilliant Blue (CBB) to visualize proteins.

Phylogenetic analyses of some conserved domains

Molecular phylogenetic analyses have been conducted on conserved domains in Pif-like and tyrosinase-domain-containing SMPs (TDC-SMPs) identified in this study. The Pif-like SMP was analyzed because it contains ChtBD2 and Laminin_G domains, as in typical Pifs[30], but it lacks the VWA domain, unlike typical Pifs. Thus, the ChtBD2 and Laminin_G domain sequences of *L. stagnalis* have been compared with those of typical Pifs in other species, so as to infer how the Pif-like SMP of *L. stagnalis* originated. The TDC-SMPs were analyzed because two types of TDC-SMPs, namely those also containing Hemocyanin_bet_s domains and those without, were identified, and their relationships remain uncertain.

Conserved domains were identified by searches with the Simple Modular Architecture Research Tool (SMART; v8.0; http://smart.embl-heidelberg.de; last accessed April 10, 2019)[40,41] provided by EMBL (European Molecular Biology Laboratory), including optional searches for outlier homologs and homologs of known structure, Pfam domains, and signal peptides. Conserved domain sequences were aligned using ClustalW[42] embedded in the phylogenetic analysis tool MEGA X[43] (v10.1.7) with default settings. Resulting alignments were submitted to the trimAl[44] (v1.2) to remove poorly aligned regions and divergent regions of protein alignment while allowing smaller final blocks, gap positions within the final blocks, and less strict flanking positions. The best-fit amino acid substitution model was inferred using the “find best protein models” function fitted in MEGA X, and phylogenetic analysis was performed using the maximum-likelihood (ML) method with bootstrap iterations of 1,000 replicates on MEGA X. Polychotomies were generated by collapsing nodes with a bootstrap value <50%.

References

1. Marie, B. *et al.* Proteomic analysis of the organic matrix of the abalone Haliotis asinina calcified shell. *Proteome Sci.* **8**, 54 (2010).
2. Marie, B., Zanella-Cléon, I., Guichard, N., Becchi, M. & Marin, F. Novel Proteins from the Calcifying Shell Matrix of the Pacific Oyster Crassostrea gigas. *Mar. Biotechnol.* **13**, 1159–1168 (2011).
3. Marie, B., Le Roy, N., Zanella-Cléon, I., Becchi, M. & Marin, F. Molecular evolution of mollusc shell proteins: Insights from proteomic analysis of the edible mussel mytilus. *J. Mol. Evol.* **72**, 531–546 (2011).
4. Marie, B. *et al.* Different secretory repertoires control the biomineralization processes of prism and nacre deposition of the pearl oyster shell. *Proc. Natl. Acad. Sci. U. S. A.* **109**, 20986–20991 (2012).
5. Liao, Z. *et al.* In-depth proteomic analysis of nacre, prism, and myostracum of Mytilus shell. *J. Proteomics* **122**, 26–40 (2015).
6. Herlitze, I., Marie, B., Marin, F., Jackson, D. J. & Jackson, D. J. Molecular modularity and asymmetry of the molluscan mantle revealed by a gene expression atlas. *Gigascience* **7**, 1–15 (2018).
7. Jackson, D. J. *et al.* The Magellania venosa biomineralizing proteome: A window into brachiopod shell evolution. *Genome Biol. Evol.* **7**, 1349–1362 (2015).
8. Isowa, Y. *et al.* Proteome analysis of shell matrix proteins in the brachiopod Laqueus rubellus. *Proteome Sci.* **13**, 1–10 (2015).
9. Freer, A., Bridgett, S., Jiang, J. & Cusack, M. Biomineral Proteins from Mytilus edulis Mantle Tissue Transcriptome. *Mar. Biotechnol.* **16**, 34–45 (2014).
10. Marin, F., Bundeleva, I., Takeuchi, T., Immel, F. & Medakovic, D. Organic matrices in metazoan calcium carbonate skeletons: Composition, functions, evolution. *J. Struct. Biol.* **196**, 98–106 (2016).
11. G Davis, C. *The many faces of epidermal growth factor repeats*. *The New biologist* vol. 2 (1990).
12. Sottrup-Jensen, L., Folkersen, J., Kristensen, T. & Tack, B. F. Partial primary structure of human pregnancy zone protein: Extensive sequence homology with human α2-macroglobulin. *Proc. Natl. Acad. Sci. U. S. A.* **81**, 7353–7357 (1984).
13. Ruggeri, Z. M. Von Willebrand factor and fibrinogen. *Curr. Opin. Cell Biol.* **5**, 898–906 (1993).
14. Bingle, L., Singleton, V. & Bingle, C. D. The putative ovarian tumour marker gene HE4 (WFDC2), is expressed in normal tissues and undergoes complex alternative splicing to yield multiple protein isoforms. *Oncogene* **21**, 2768–2773 (2002).
15. Gibbs, G. M., Roelants, K. & O'Bryan, M. K. The CAP Superfamily: Cysteine-Rich

Secretory Proteins, Antigen 5, and Pathogenesis-Related 1 Proteins—Roles in Reproduction, Cancer, and Immune Defense. *Endocr. Rev.* **29**, 865–897 (2008).
16. Okamoto, O. & Fujiwara, S. Dermatopontin, a novel player in the biology of the extracellular matrix. *Connect. Tissue Res.* **47**, 177–189 (2006).
17. Okamoto, O. *et al.* Dermatopontin promotes epidermal keratinocyte adhesion via α3β1 integrin and a proteoglycan receptor. *Biochemistry* **49**, 147–155 (2010).
18. Shashoua, V. E. Ependymin, a Brain Extracellular Glycoprotein, and CNS Plasticity. *Ann. N. Y. Acad. Sci.* **627**, 94–114 (1991).
19. Sharon, N. & Lis, H. The structural basis for carbohydrate recognition by lectins. *Adv. Exp. Med. Biol.* **491**, 1–16 (2001).
20. Mann, K., Weiss, I. M., André, S., Gabius, H. J. & Fritz, M. The amino-acid sequence of the abalone (Haliotis laevigata) nacre protein perlucin: Detection of a functional C-type lectin domain with galactose/mannose specificity. *Eur. J. Biochem.* **267**, 5257–5264 (2000).
21. Shen, Z. & Jacobs-Lorenat, M. A type I peritrophic matrix protein from the malaria vector Anopheles gambiae binds to chitin. Cloning, expression, and characterization. *J. Biol. Chem.* **273**, 17665–17670 (1998).
22. Ban, C., Ramakrishnan, B., Ling, K.-Y., Kung, C. & Sundaralingam, M. Structure of the recombinant Paramecium tetraurelia calmodulin at 1.68 Å resolution. *Acta Crystallogr. Sect. D Biol. Crystallogr.* **50**, 50–63 (1994).
23. Bento, I., Martins, L. O., Gato Lopes, G., Arménia Carrondo, M. & Lindley, P. F. Dioxygen reduction by multi-copper oxidases; a structural perspective. *Dalt. Trans.* **4**, 3507 (2005).
24. Kato, S., Matsui, T., Gatsogiannis, C. & Tanaka, Y. Molluscan hemocyanin: structure, evolution, and physiology. *Biophys. Rev.* **10**, 191–202 (2018).
25. Nelson, R. E. *et al.* Peroxidasin: a novel enzyme-matrix protein of Drosophila development. *EMBO J.* **13**, 3438–3447 (1994).
26. Li, H. & Poulos, T. L. Structural variation in heme enzymes: a comparative analysis of peroxidase and P450 crystal structures. *Structure* **2**, 461–464 (1994).
27. Kimura, S. & Ikeda - Saito, M. Human myeloperoxidase and thyroid peroxidase, two enzymes with separate and distinct physiological functions, are evolutionarily related members of the same gene family. *Proteins Struct. Funct. Bioinforma.* **3**, 113–120 (1988).
28. Vinet, L. & Zhedanov, A. A 'missing' family of classical orthogonal polynomials. *J. Phys. A Math. Theor.* **44**, 085201 (2011).
29. Sarashina, I. & Endo, K. Skeletal matrix proteins of invertebrate animals: Comparative analysis of their amino acid sequences. *Paleontol. Res.* **10**, 311–336 (2006).

30. Suzuki, M. *et al.* An acidic matrix protein, Pif, is a key macromolecule for nacre formation. *Science (80-.).* **325**, 1388–1390 (2009).
31. Suzuki, M., Iwashima, A., Kimura, M., Kogure, T. & Nagasawa, H. The Molecular Evolution of the Pif Family Proteins in Various Species of Mollusks. *Mar. Biotechnol.* **15**, 145–158 (2013).
32. Zhao, R. *et al.* Dual gene repertoires for larval and adult shells reveal molecules essential for molluscan shell formation. *Mol. Biol. Evol.* **35**, 2751–2761 (2018).
33. Mann, K., Edsinger-Gonzales, E. & Mann, M. In-depth proteomic analysis of a mollusc shell: Acid-soluble and acid-insoluble matrix of the limpet Lottia gigantea. *Proteome Sci.* **10**, 1–18 (2012).
34. Zhang, G. *et al.* The oyster genome reveals stress adaptation and complexity of shell formation. *Nature* **490**, 49–54 (2012).
35. Feng, D., Li, Q., Yu, H., Kong, L. & Du, S. Identification of conserved proteins from diverse shell matrix proteome in Crassostrea gigas: characterization of genetic bases regulating shell formation. *Sci. Rep.* **7**, 45754 (2017).
36. Takeuchi, T. *et al.* Bivalve-specific gene expansion in the pearl oyster genome: implications of adaptation to a sessile lifestyle. *Zool. Lett.* **2**, 3 (2016).
37. Weiss, I. M., Kaufmann, S., Mann, K. & Fritz, M. Purification and characterization of perlucin and perlustrin, two new proteins from the shell of the mollusc Haliotis laevigata. *Biochem. Biophys. Res. Commun.* **267**, 17–21 (2000).
38. Wang, N., Lee, Y. H. & Lee, J. Recombinant perlucin nucleates the growth of calcium carbonate crystals: Molecular cloning and characterization of perlucin from disk abalone, Haliotis discus discus. *Comp. Biochem. Physiol. - B Biochem. Mol. Biol.* **149**, 354–361 (2008).
39. Jackson, D. J. *et al.* Parallel Evolution of Nacre Building Gene Sets in Molluscs. *Mol. Biol. Evol.* **27**, 591–608 (2010).
40. Schultz, J., Milpetz, F., Bork, P. & Ponting, C. P. SMART, a simple modular architecture research tool: Identification of signaling domains. *Proc. Natl. Acad. Sci. U. S. A.* **95**, 5857–5864 (1998).
41. Letunic, I. & Bork, P. 20 years of the SMART protein domain annotation resource. *Nucleic Acids Res.* **46**, D493–D496 (2018).
42. Thompson, J. D., Higgins, D. G. & Gibson, T. J. CLUSTAL W: Improving the sensitivity of progressive multiple sequence alignment through sequence weighting, position-specific gap penalties and weight matrix choice. *Nucleic Acids Res.* **22**, 4673–4680 (1994).
43. Kumar, S., Stecher, G., Li, M., Knyaz, C. & Tamura, K. MEGA X: Molecular evolutionary genetics analysis across computing platforms. *Mol. Biol. Evol.* **35**, 1547–1549 (2018).

44. Capella-Gutiérrez, S., Silla-Martínez, J. M. & Gabaldón, T. trimAl: a tool for automated alignment trimming in large-scale phylogenetic analyses. *Bioinforma. Appl. NOTE* **25**, 1972–1973 (2009).
45. Mann, K. & Jackson, D. J. Characterization of the pigmented shell-forming proteome of the common grove snail Cepaea nemoralis. *BMC Genomics* **15**, 249 (2014).
46. Shimizu, K. *et al.* Insights into the Evolution of Shells and Love Darts of Land Snails Revealed from Their Matrix Proteins. *Genome Biol. Evol.* **11**, 380–397 (2019).

Supplementary Figure S1.

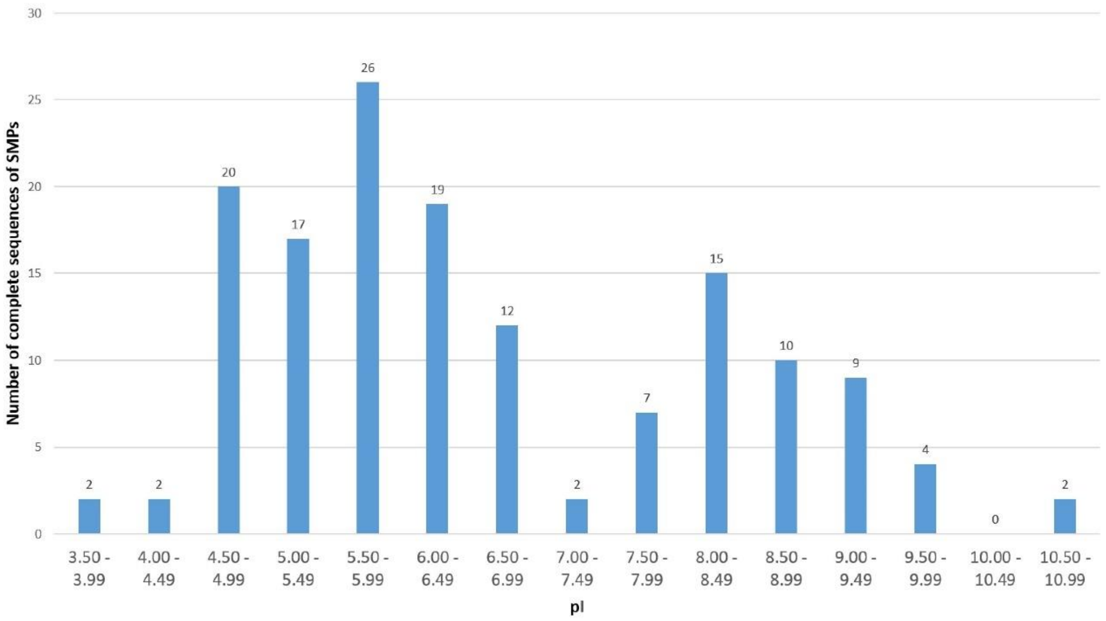

Supplementary Figure S1.

Histogram showing the frequencies of theoretical pIs of SMPs identified in this study. Theoretical pI was estimated from the amino acid sequence translated using UniProtKB. The values of the highest and the lowest pIs were 10.90 and 3.65, respectively.

Supplementary Figure S2.

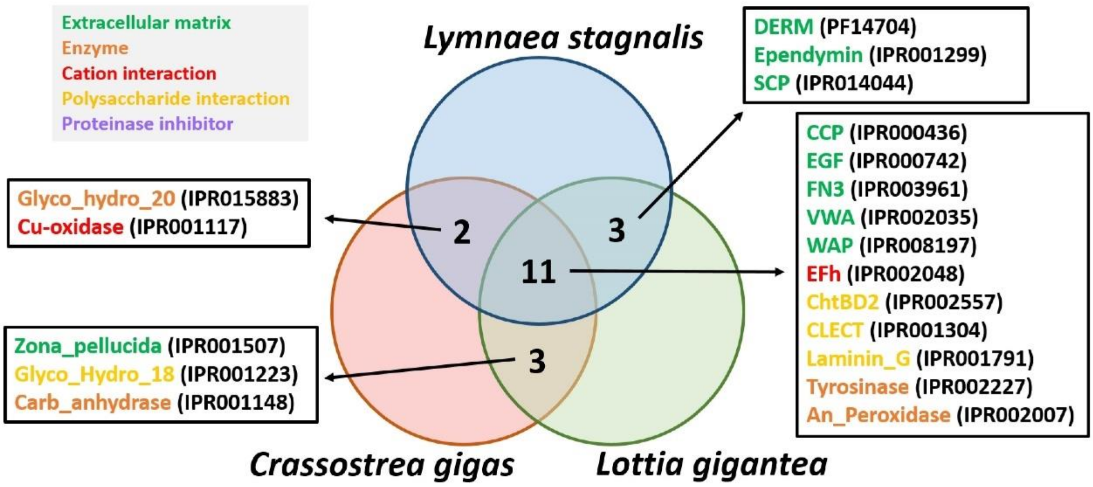

Supplementary Figure S2.

Venn diagram showing shared conserved domains among SMPs of three mollusc species (*Lymnaea stagnalis*, *Crassostrea gigas*, and *Lottia gigantea*). Conserved domains are grouped into 5 categories. 11 domains (CCP, EGF, FN3, VWA, WAP, EFh, ChtBD2, CLECT, Laminin_G, Tyrosinase, and An_Peroxidase) are shared among all three species. 2 domains (Glyco_hydro_20, and Cu-oxidase) are shared between *L. stagnalis* and *C. gigas*, and 3 domains (Ependymin, DERM, and SCP) between *L. stagnalis* and *L. gigantea*.

Supplementary Figure S3. (continued)

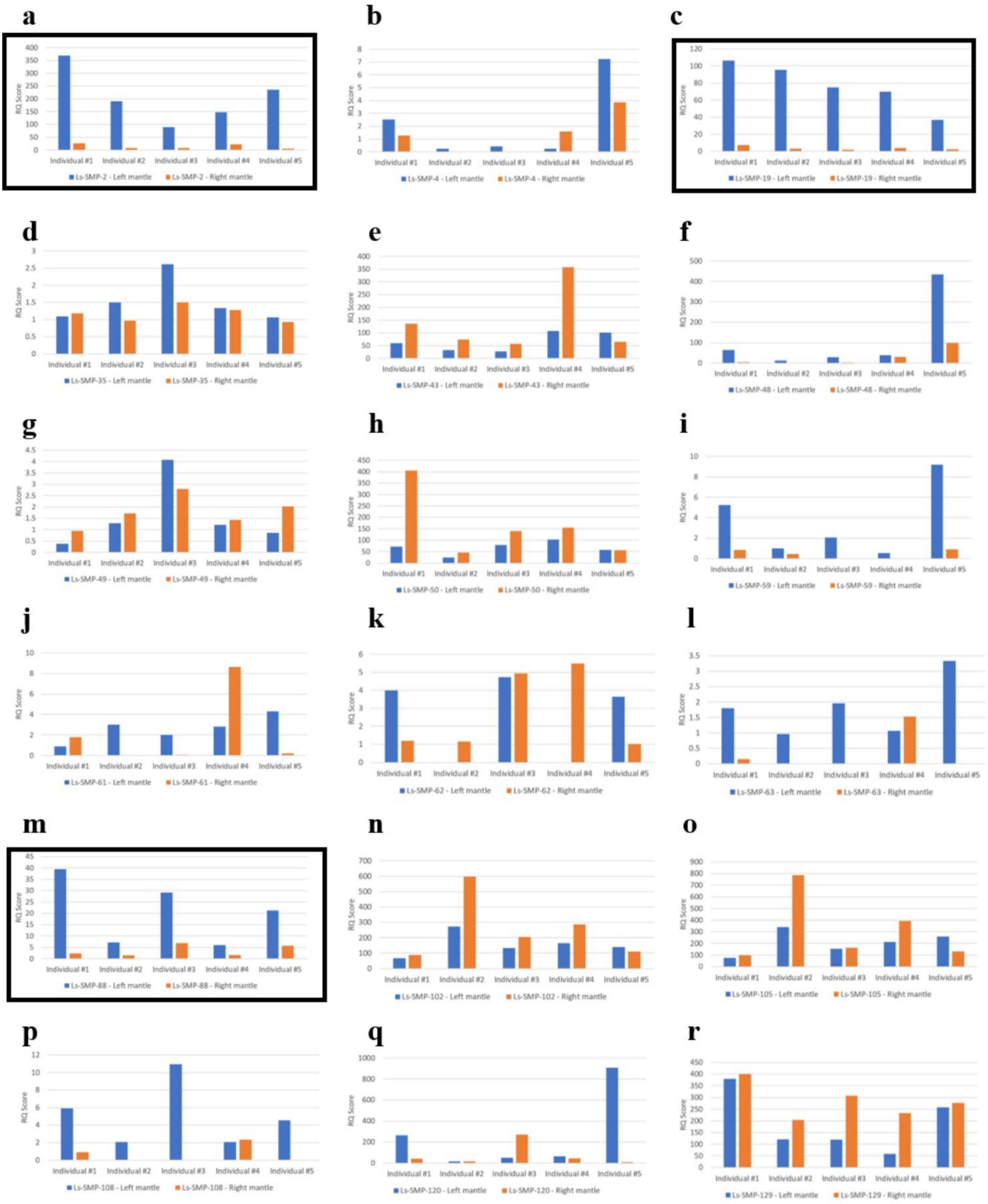

Supplementary Figure S3. (continued)

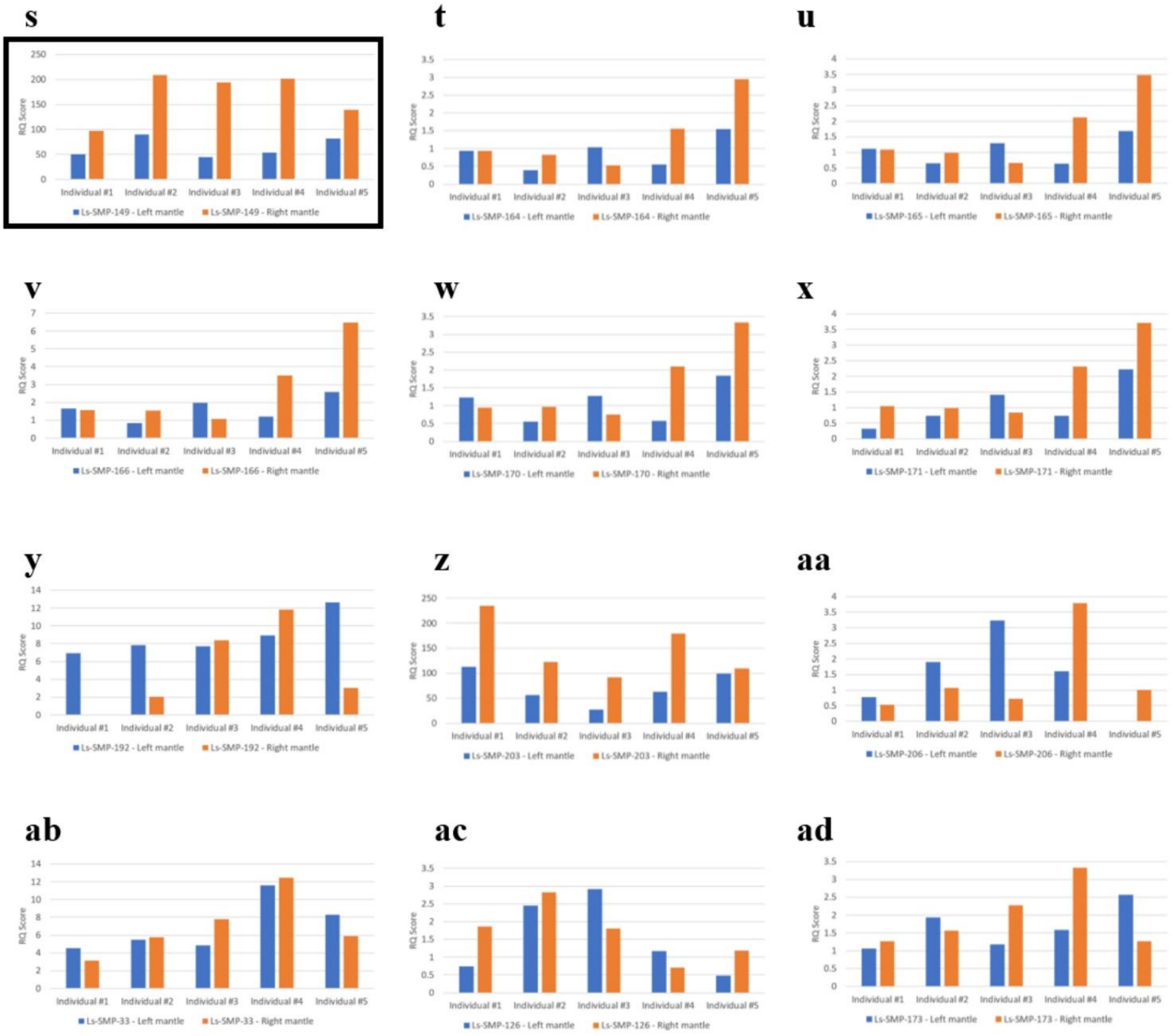

Supplementary Figure S3.

Results of qPCR analysis. RQ (Relative Quantitation) scores are shown for 27 successfully amplified asymmetrically expressed SMP genes in the transcriptomic analysis (a to aa) as well as the three symmetrically expressed SMP genes (as control; ab to ad). Quantitative PCR was performed on each left and right mantle sample for 5 biological replicates. Relative expression levels (RQ Scores) of two technical replicates for each individual are shown. The four SMP genes (a: Ls-SMP-2, c: Ls-SMP-19, m: Ls-SMP-88, and s: Ls-SMP-149) that indicated exactly the same asymmetric trends as in transcriptomic analysis are shown by a black rectangle. Five SMP genes (Ls-SMP-30, Ls-SMP-39, Ls-SMP-60, Ls-SMP-70, and Ls-SMP-150) out of the 32 asymmetrically expressed SMP genes in the transcriptomic analysis did not show detectable signals (Fig. 5).

Supplementary Figure S4.

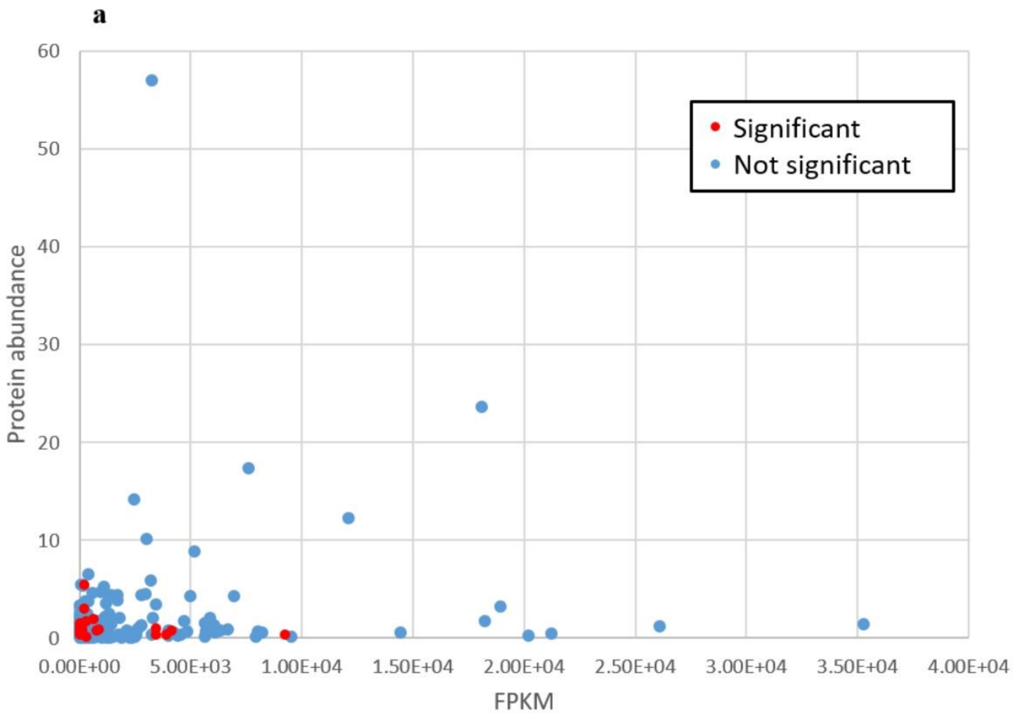

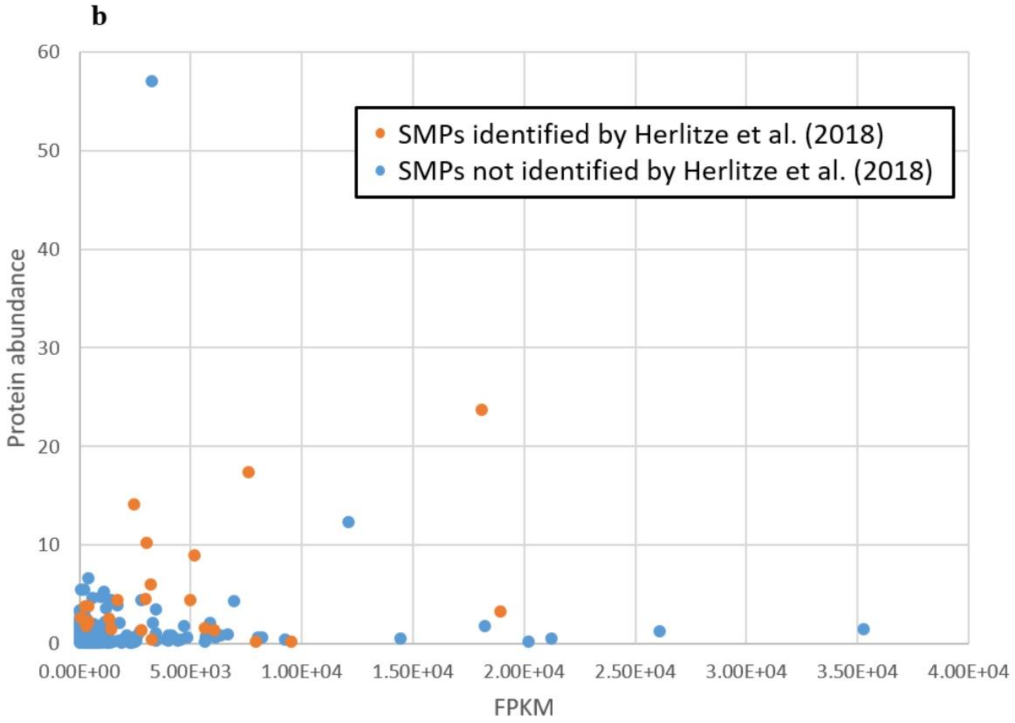

Supplementary Figure S4.

Graphs showing comparisons of FPKM and protein abundance for identified SMPs. (a) SMPs displaying significant differences between left and right sides of the mantle are shown in red. (b) SMPs that were also identified by Herlitze et al. (2018)[6] are shown in orange.

Supplementary Figure S5.

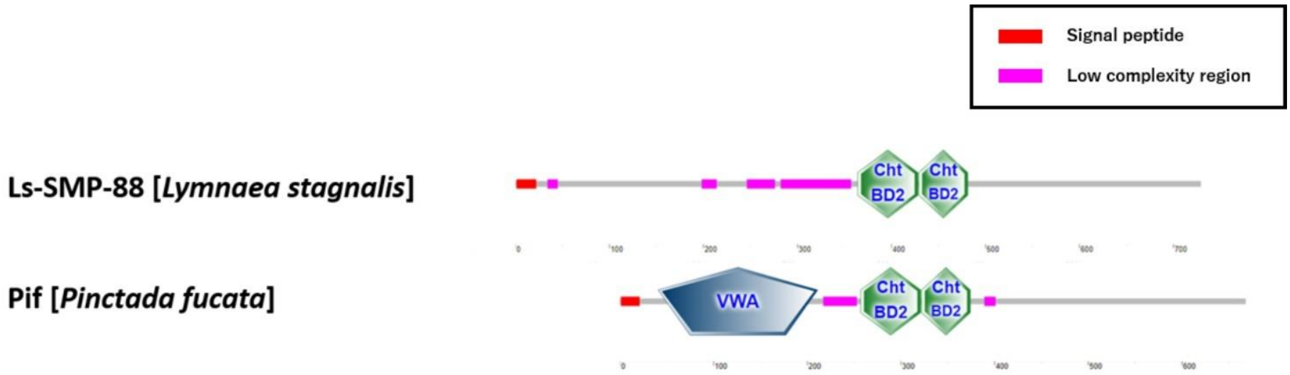

Supplementary Figure S5.

Schematic representations of the conserved domain configuration of Ls-SMP-88 of *Lymnaea stagnalis* and Pif of *Pinctada fucata* (results from SMART domain searches). A Laminin_G domain was not identified by SMART domain searches in those proteins, but was identified by Pfam domain searches in the region just downstream of the ChtBD2 domains in both Ls-SMP-88 and Pif.

Supplementary Figure S6.

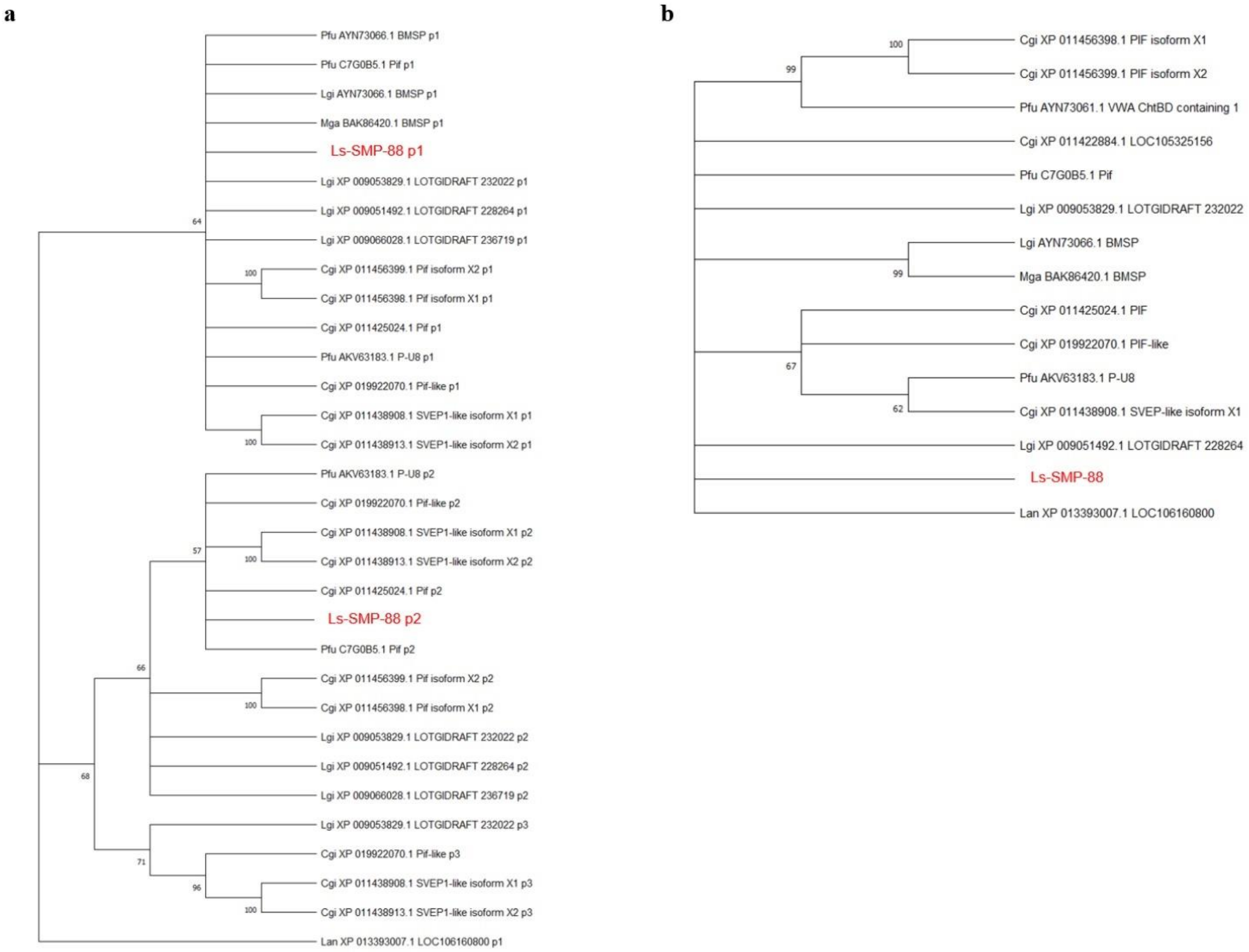

Supplementary Figure S6.

(a) Maximum likelihood tree of ChtBD2 domains in molluscan SMPs, including the one from *Lymnaea stagnalis*. Molluscan ChtBD2 domain sequences were retrieved from NCBI GenBank after BLAST searches using the two ChtBD2 domain sequences of Ls-SMP-88 of *L. stagnalis* as query. The ML tree was inferred from two domains of Ls-SMP-88 and 29 domains of 14 proteins, using the WAG + G + I model based on 47 amino acids. Polychotomy results if the bootstrap value of the node is lower than 50%. Bootstrap values are indicated for nodes with a value greater >50%. A domain sequence of the chitin-binding domain containing protein of the brachiopod, *Lingula anatina*, was included as an outgroup. Sequence names in red indicate sequences from *L. stagnalis*. Cgi: *Crassostrea gigas*, Lan: *Lingula anatina*, Lgi: *Lottia gigantea*, Mga: *Mytilus galloprovincialis*, Pfu: *Pinctada fucata*. When more than one ChtBD2 domain exists in a protein, they are discriminated in order from the N-terminus, and named p1, p2, etc. (b) Maximum likelihood tree of Laminin_G domains in molluscan SMPs, including the one identified from *Lymnaea stagnalis*. Molluscan Laminin_G domain sequences were retrieved from NCBI GenBank after BLAST searches using the Laminin_G domain sequence of Ls-SMP-88 of *L. stagnalis* as a query. The ML tree was inferred from a domain of Ls-SMP-88 and 13 domains of 13 proteins, using the LG + G model, based on 128 amino acids. Polychotomy results if the bootstrap value of the node is lower than 50%. Bootstrap values are indicated for nodes with a value greater >50%. A domain sequence of the chitin-binding domain-containing protein of the brachiopod, *Lingula anatina*, was included as outgroup. The sequence name in red indicates the sequence from *L. stagnalis*. Cgi: *Crassostrea gigas*, Lan: *Lingula anatina*, Lgi: *Lottia gigantea*, Mga: *Mytilus galloprovincialis*, Pfu: *Pinctada fucata*.

Supplementary Figure S7.

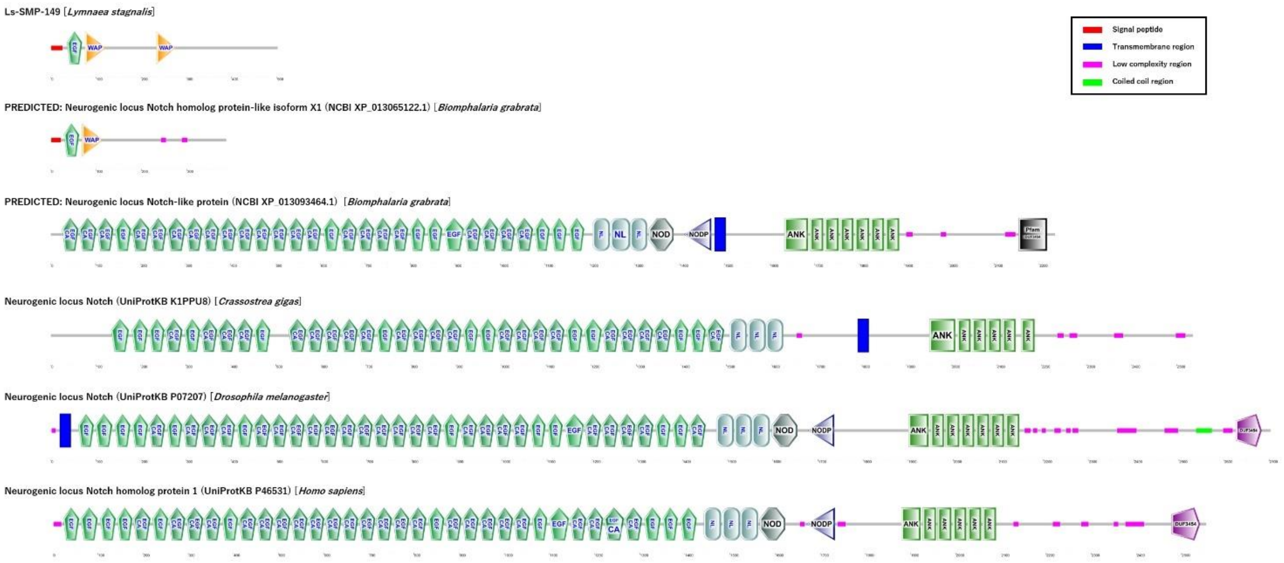

Supplementary Figure S7.

Schematic representations of the conserved domain configuration of Ls-SMP-149 and neurogenic locus Notch proteins and a related protein of other species (*Biomphalaria grabrata*, *Crassostrea gigas*, *Drosophila melanogaster*, and *Homo sapiens*) (results from SMART domain searches).

Supplementary Figure S8.

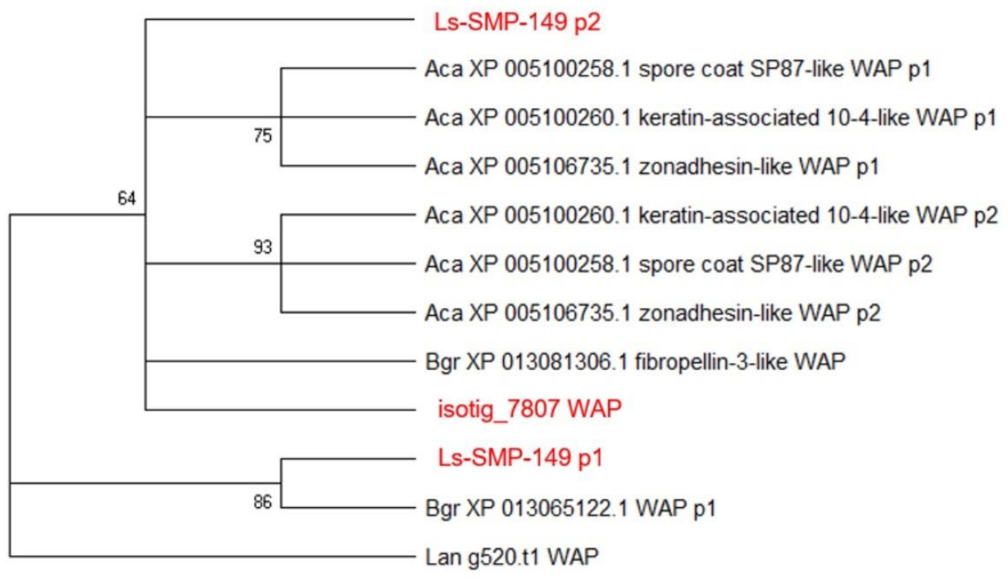

Supplementary Figure S8.

Maximum likelihood tree of the WAP domains in molluscan proteins, including SMPs identified from *Lymnaea stagnalis* and *Cepaea nemoralis* in this study or by Mann and Jackson (2014)[45]. The ML tree was inferred from 12 domain sequences of 7 proteins, including one domain sequence from one protein of the brachiopod, *Lingula anatina* as an outgroup, using the JTT + G + I model based on 35 amino acids. Polychotomy results if the bootstrap value of the node is lower than 50%. Bootstrap values are indicated for nodes with a value greater >50%. Molluscan WAP domain sequences were retrieved from NCBI GenBank after BLAST searches using the WAP domain sequence of Ls-SMP-149 of *L. stagnalis* as a query. Bootstrap values are indicated for each node. Sequence names in red indicate SMP sequences from *L. stagnalis* (Ls-SMP-149), and the land snail, *C. nemoralis* (isotig_7807). Ana: Aplysia californica, Bgr: *Biomphalaria grabrata*, Cne: *Cepaea nemoralis*, Lan: *Lingula anatina*. When more than one WAP domain exists in a protein, they are given in order from the N-terminus, and named p1, p2, etc.

Supplementary Figure S9.

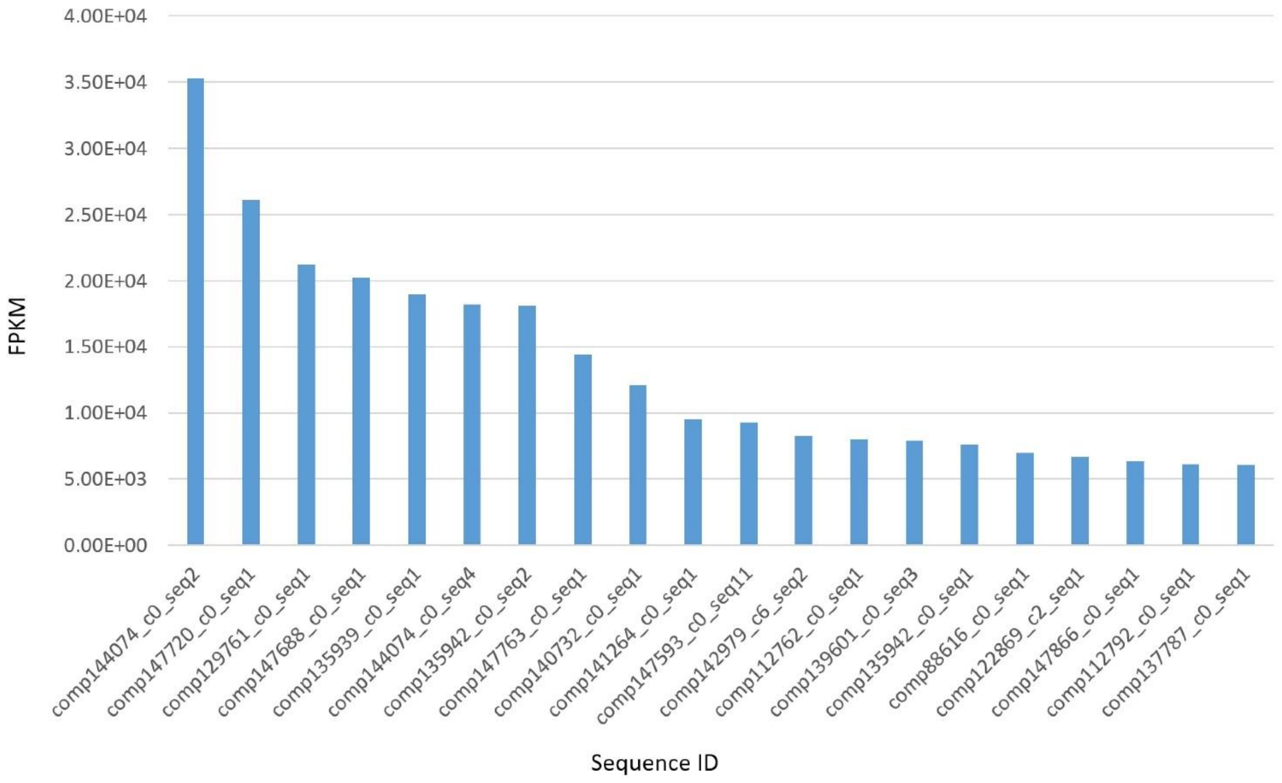

Supplementary Figure S9.

Top 10% of contigs for SMP-coding genes of *L. stagnalis* that yielded the highest FPKM values.

Supplementary Figure S10.

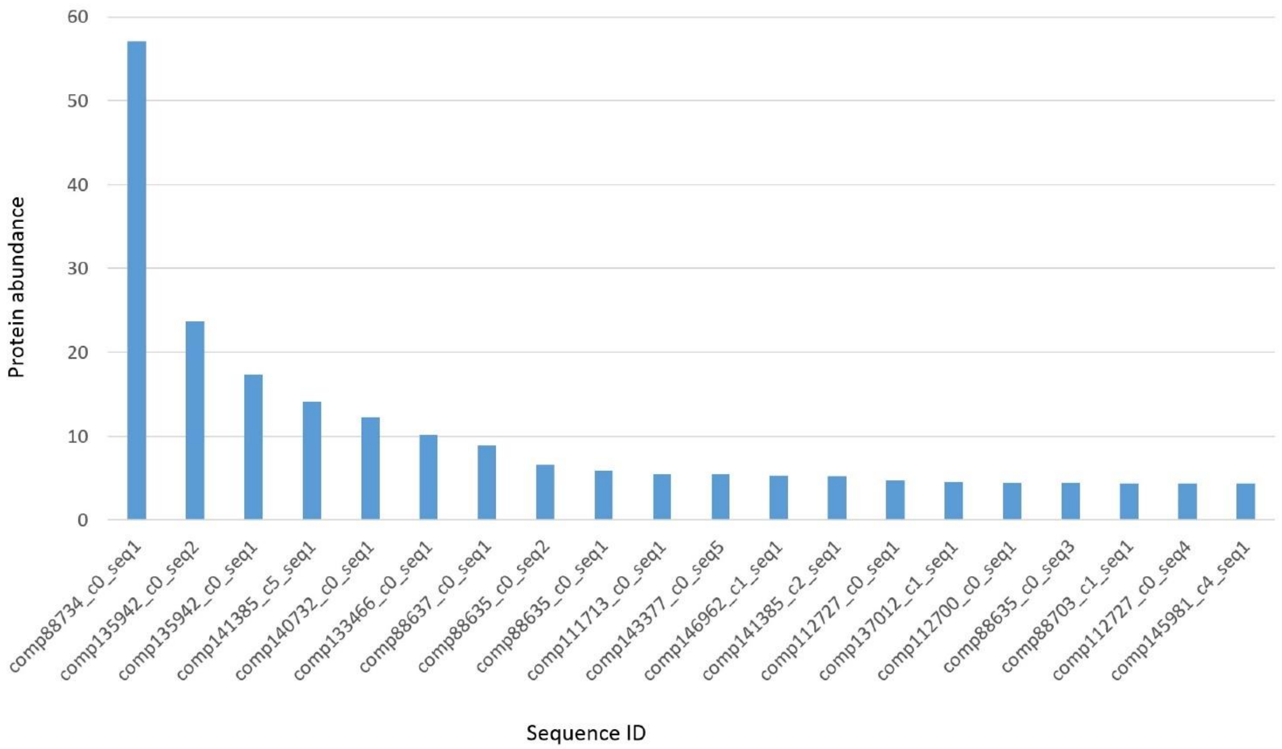

Supplementary Figure S10.

Top 10% of SMPs (shown in contig names) yielding the highest protein abundance values.

Supplementary Figure S11.

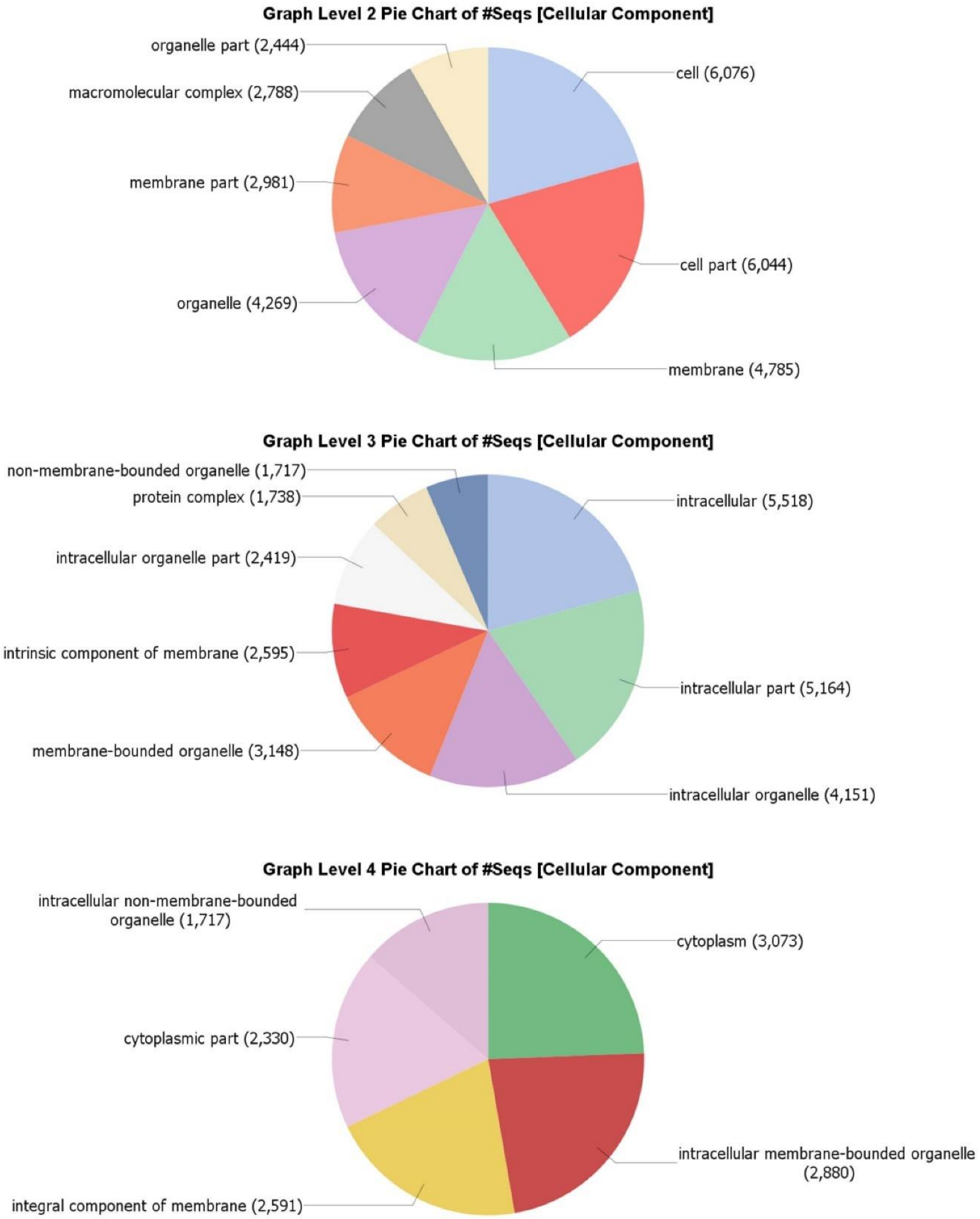

Supplementary Figure S11.

Combined graphs for GO of mantle tissues (Cellular Component) produced by Blast2GO.

Supplementary Figure S12.

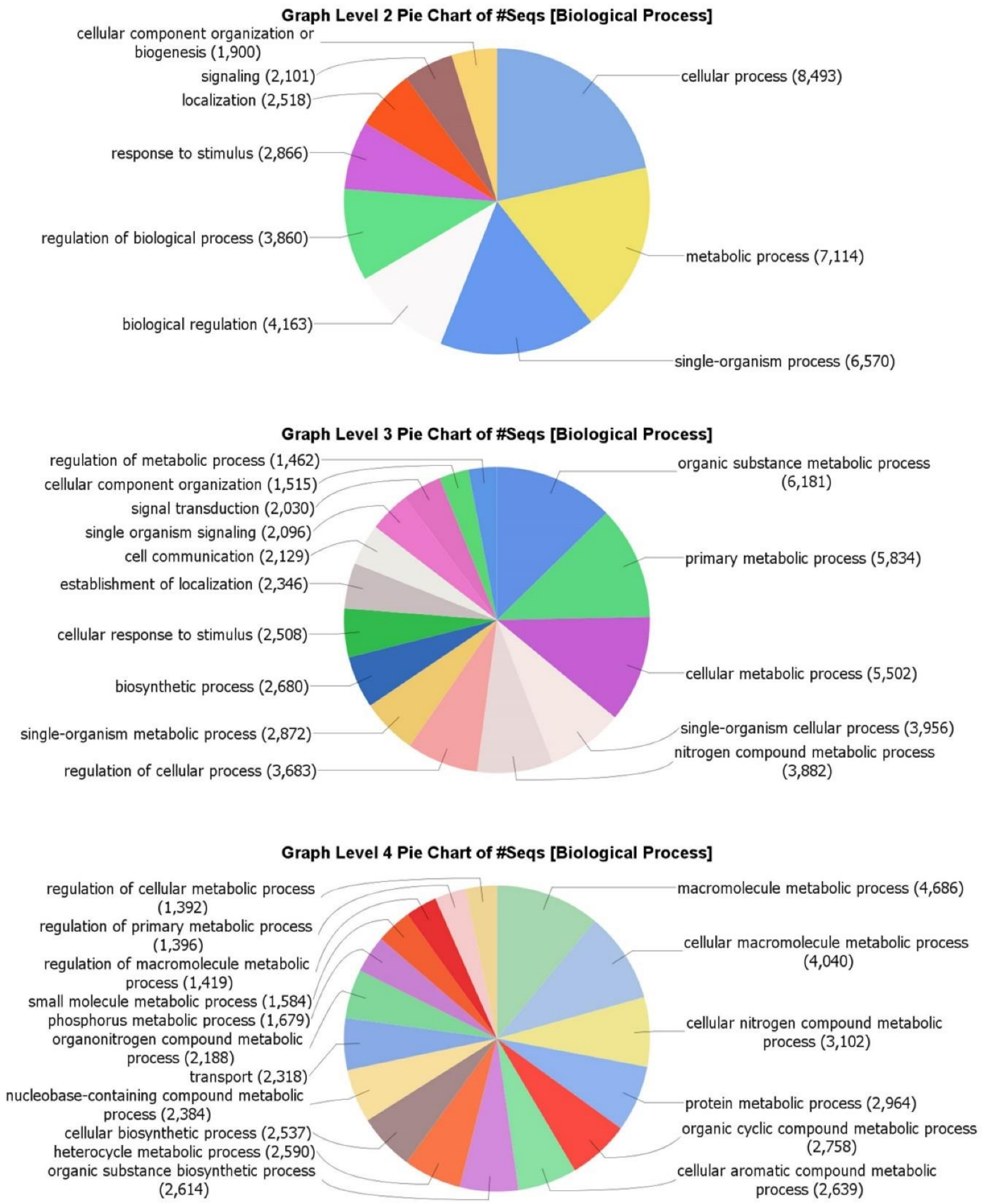

Supplementary Figure S12.
Combined graphs for GO of mantle tissues (Biological Process) produced by Blast2GO.

Supplementary Figure S13.

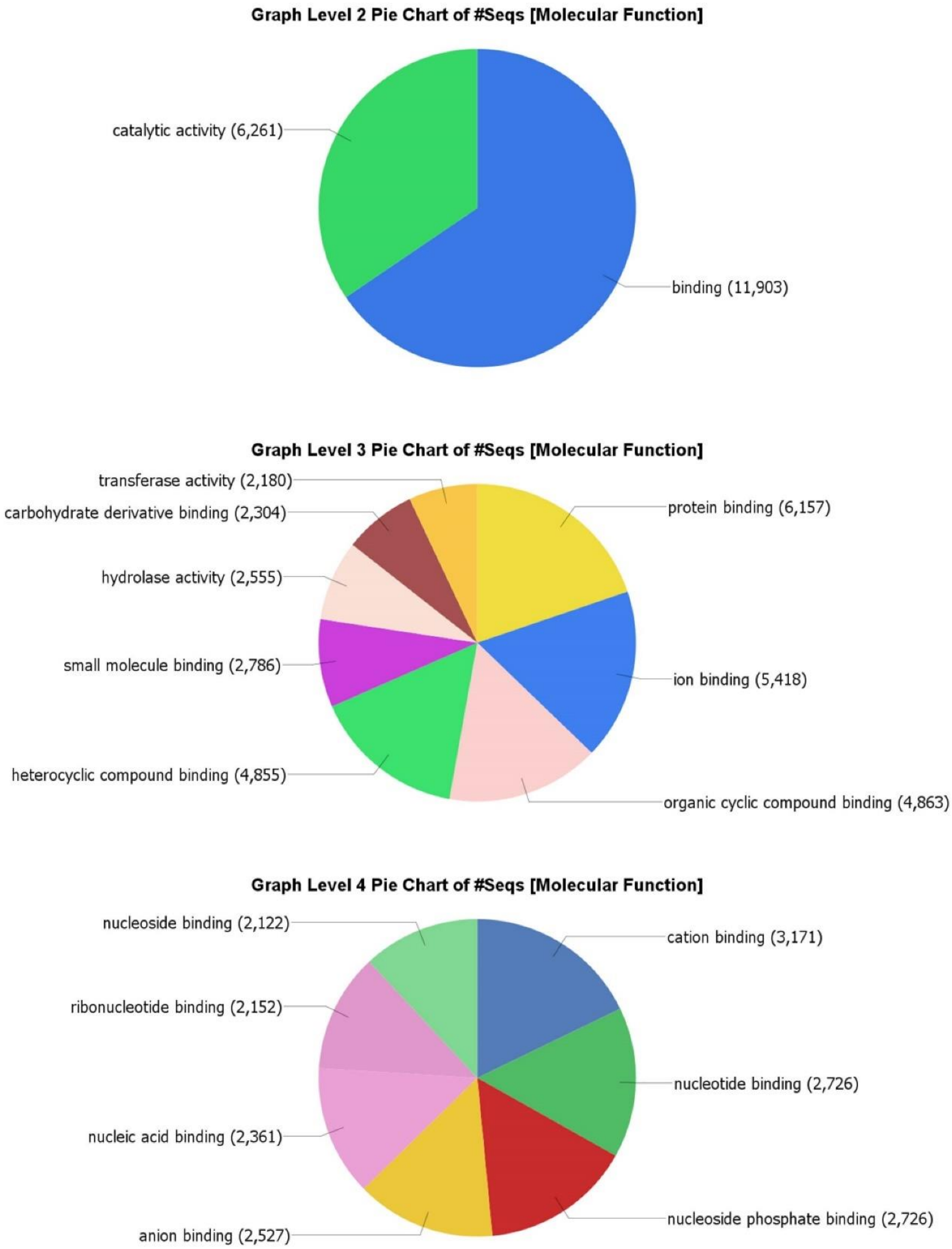

Supplementary Figure S13.

Combined graphs for GO of mantle tissues (Molecular Function) produced by Blast2GO.

Supplementary Figure S14.

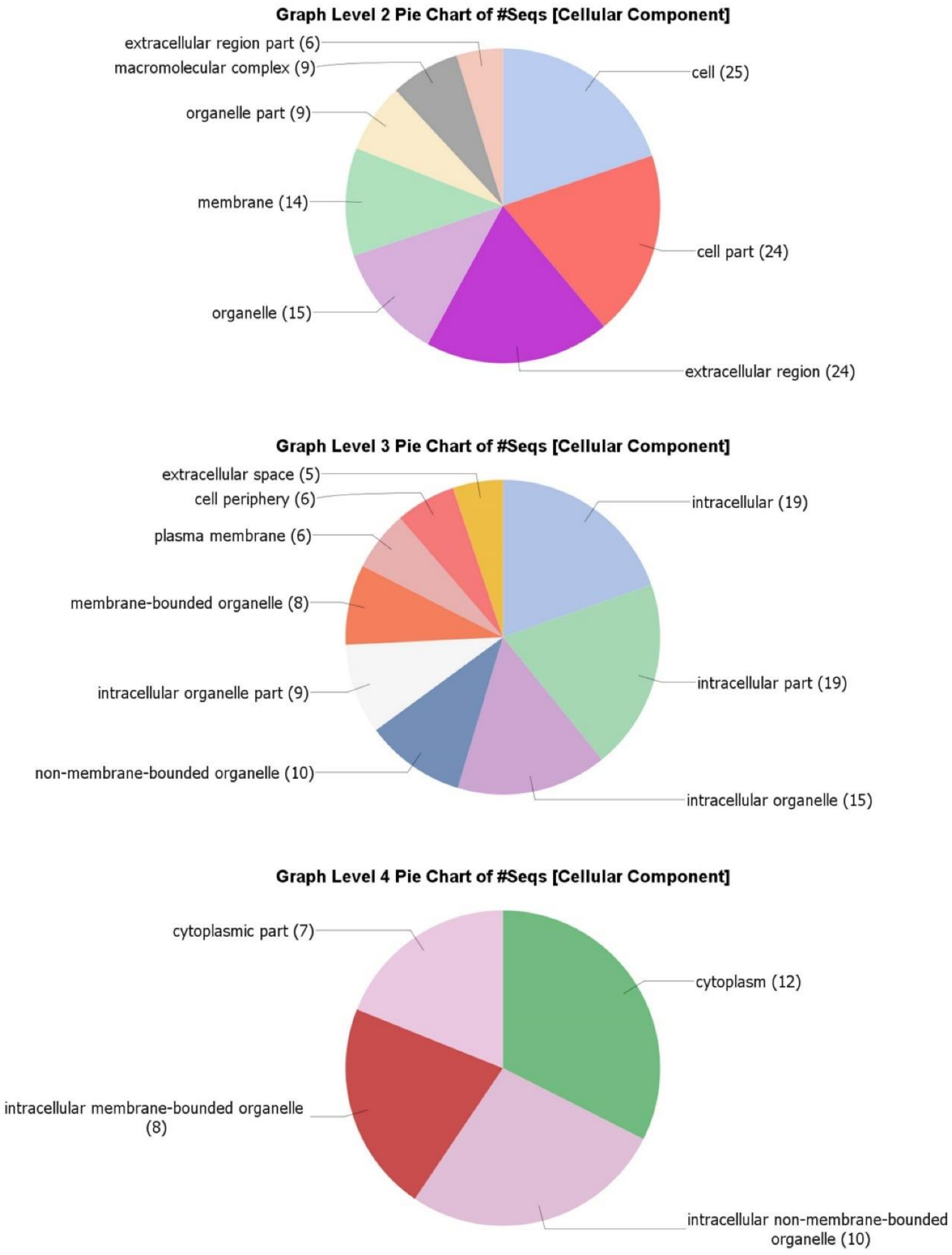

Supplementary Figure S14.
Combined graphs for GO of SMPs (Cellular Component) produced by Blast2GO.

Supplementary Figure S15.

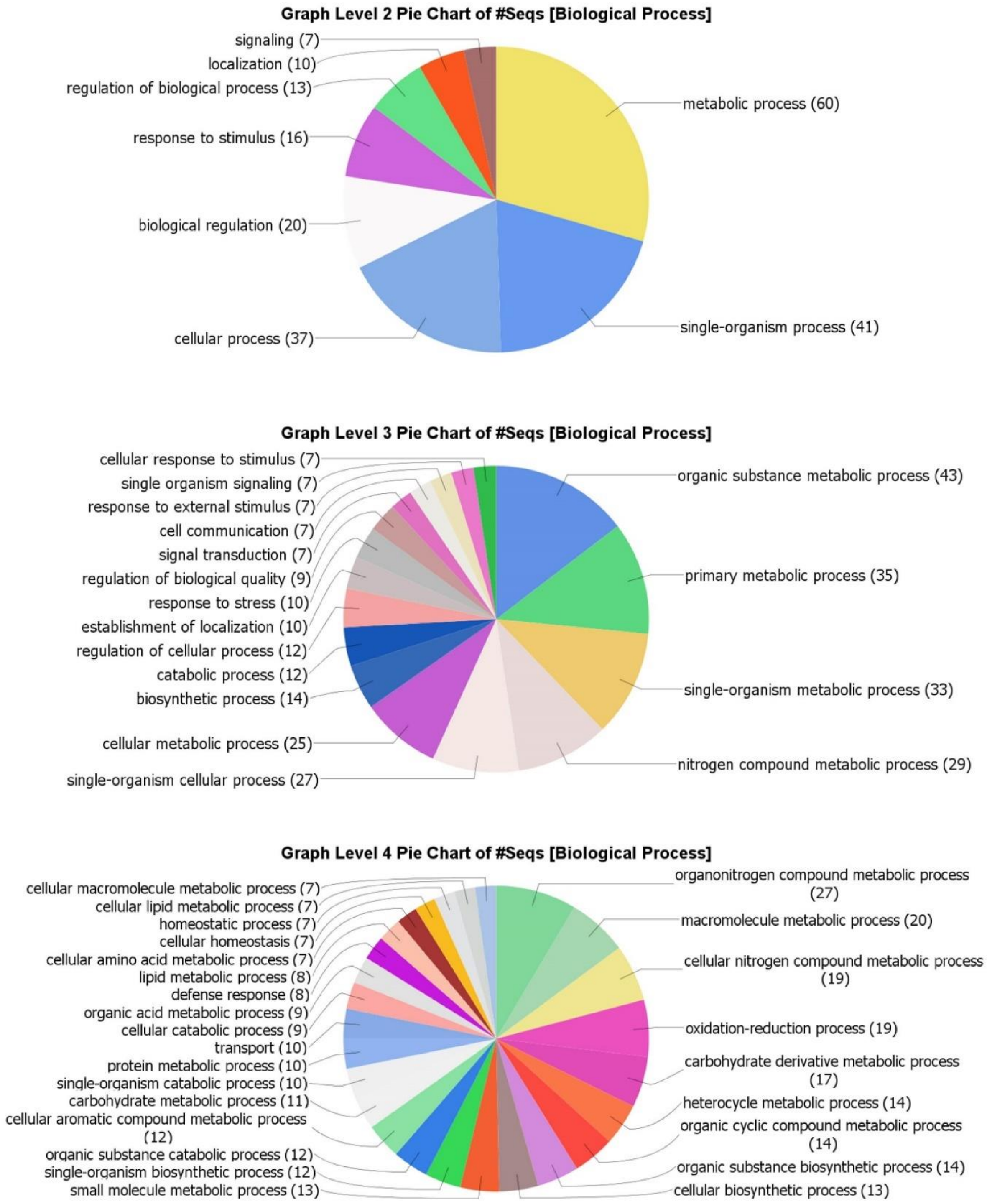

Supplementary Figure S15.

Combined graphs for GO of SMPs (Biological Process) produced by Blast2GO.

Supplementary Figure S16.

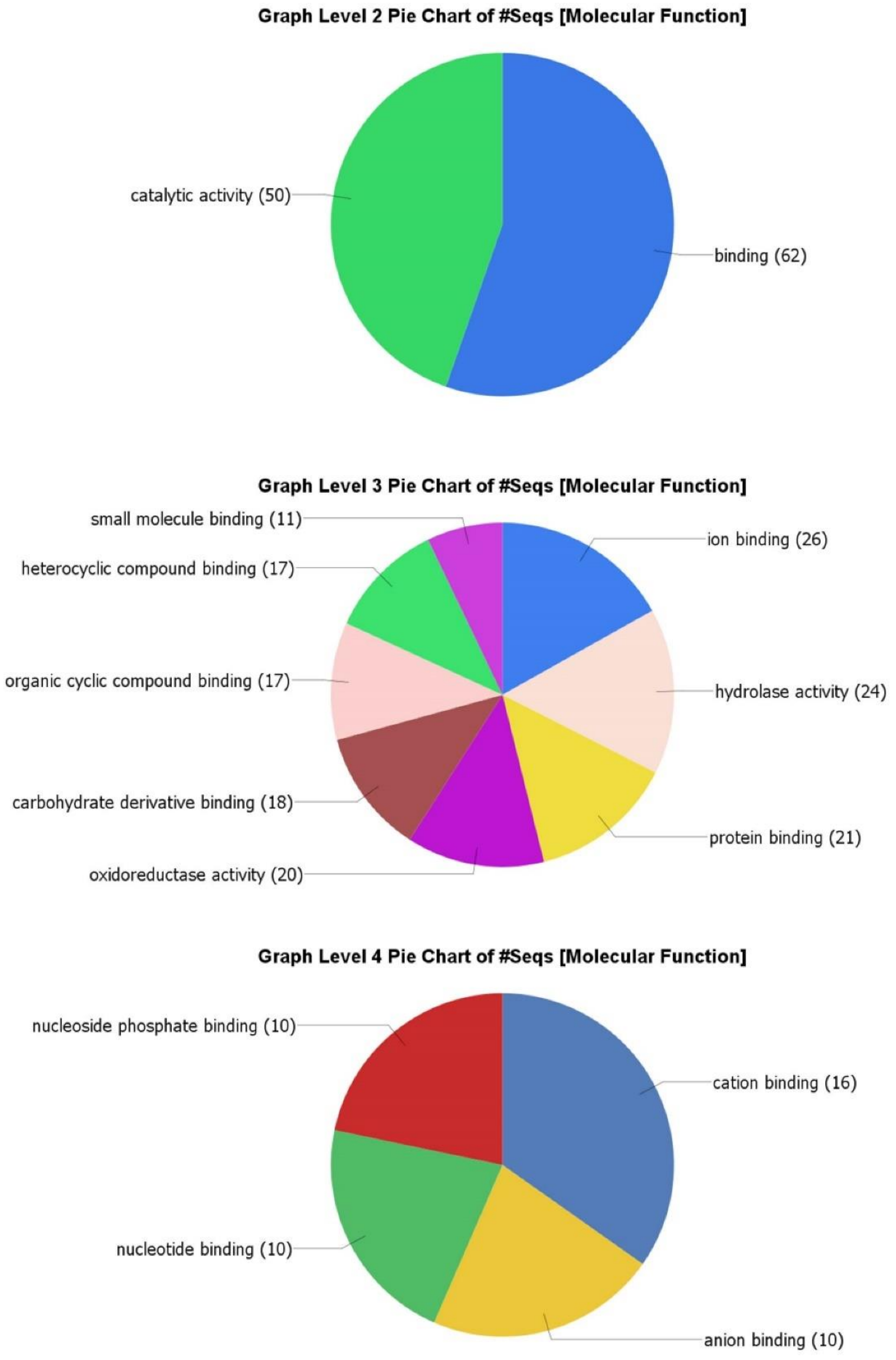

Supplementary Figure S16.
Combined graphs for GO of SMPs (Molecular Function) produced by Blast2GO.

Supplementary Figure S17.

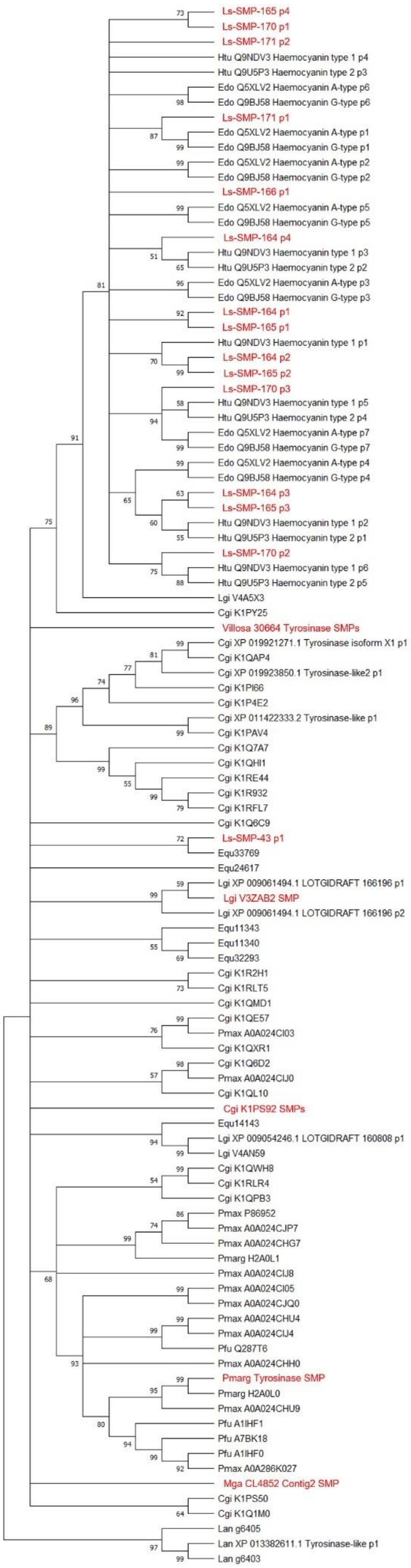

Supplementary Figure S17.

Maximum likelihood tree of the tyrosinase domains contained in some animal tyrosinase proteins and molluscan SMPs, including the one from *Lymnaea stagnalis*. The ML tree was inferred from 15 domains in 6 SMPs of *L. stagnalis*, 25 domains in two molluscan haemocyanin proteins from NCBI GenBank and 63 domains in tyrosinase proteins identified by Shimizu et al. (2019)[46], using the LG + G model based on 82 amino acids. Polychotomy results if the bootstrap value of the node is lower than 50%. Bootstrap values are indicated for nodes with a value greater >50%. Sequences of tyrosinase domains of the brachiopod, *Lingula anatina*, were included as an outgroup. Sequence names in red indicate the sequences from *L. stagnalis*, and red circles indicate proteins that have been identified as SMPs in this study or by Shimizu et al. (2019)[46]. Cgi: *Crassostrea gigas*, Equ: *Euhadra quaesita*, Lan: *Lingula anatina*, Lgi: *Lottia gigantea*, Mga: *Mytilus galloprovincialis*, Pmarg: *Pinctada margaritifera*, Pmax: *Pinctada. maxima*, Pfu: *Pinctada fucata*. When more than one tyrosinase domain exists in a protein, they are listed in order from the N-terminus, and named p1, p2, etc.

Supplementary Figure S18.

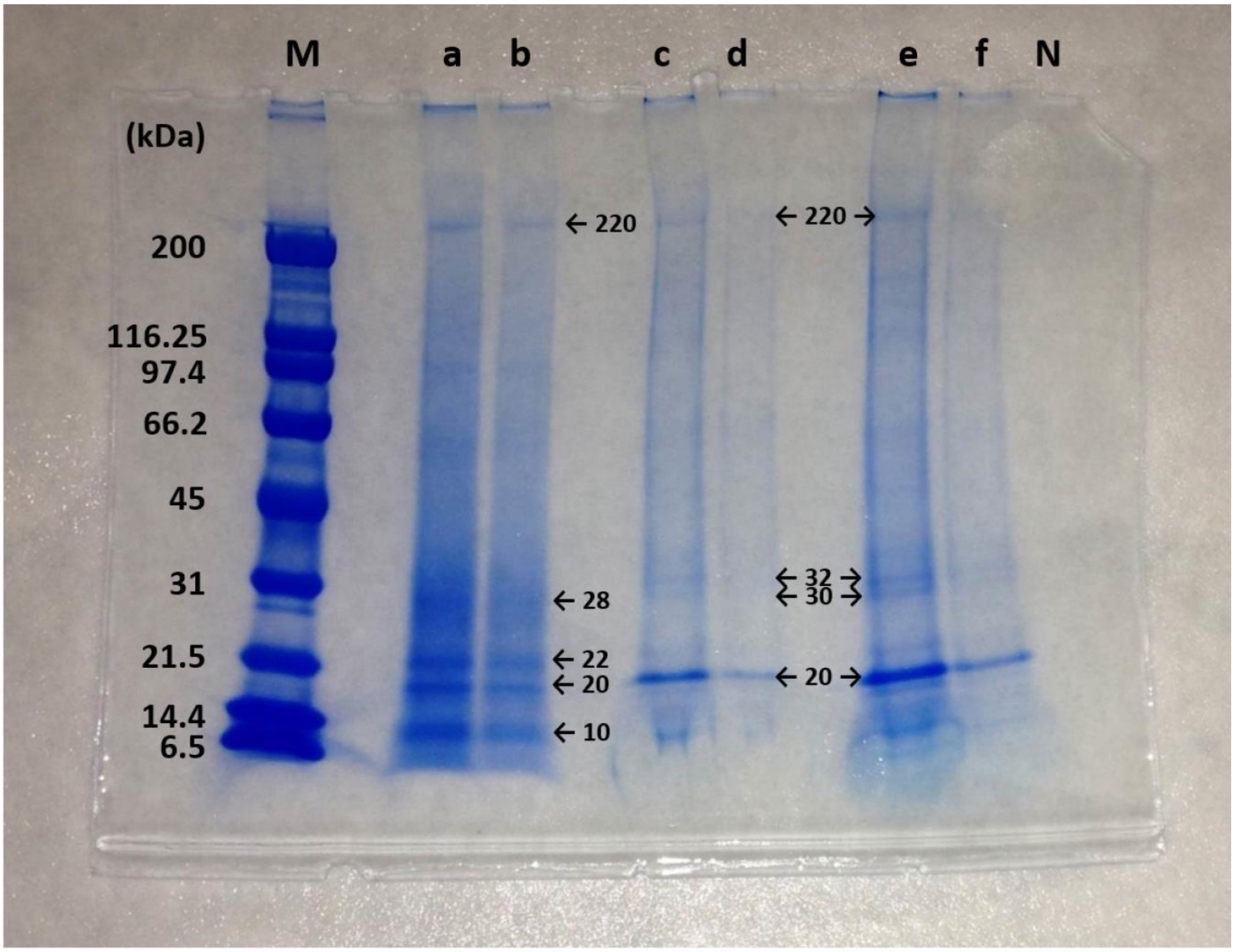

Supplementary Figure S18.

SDS-PAGE analysis of soluble and insoluble fractions extracted from shells of *L. stagnalis*. M: Marker, a: soluble fraction (904 μg/mL), b: soluble fraction (452 μg/mL), c: insoluble fraction (40 μg/mL), d: insoluble fraction (10 μg/mL).

Supplementary Figure S19.

```
Ls-SMP-195 comp88616_c0_seq1 complete   -MSILASVVLFVAVAAAAEAAYVNQLGQPFDFKCPPGQIISHIGSDYDLL  49
BAD97850.1 Dermatopontin1 partial       --------------------------------------------------
Ls-SMP-147 comp146160_c2_seq8 complete  -MLSVAQLLCLAAIASLLVEGYVNEMDKPFDFKCPSGQIMFFASSVHSNY  49
BAD97851.1 Dermatopontin2 partial       ----------------------------------------FFASSVHSNY  10
Ls-SMP-184 comp150744_c0_seq1 complete  MAGLISGFFCIAFLIAGSTAGFVNDWDQPFNFNCPSGQIINFVSSIHNNR  50
BAD97852.1 Dermatopontin3 partial       ----------------------------------------DFVSSIHNNR  10
Ls-SMP-23 comp125384_c0_seq1 complete   ---MKISVVIACVFLCFVDVQMSKQANKYPDFECYGKRITEIIE------  41
Ls-SMP-24 comp125384_c0_seq2 3'partial  ---MKISVVIACVFVCFVEVQMSNQANKYPDFECYGKRITEIIE------  41

Ls-SMP-195 comp88616_c0_seq1  complete  LEDRQWEFRCRAAN--VSEVCSSSGYVNGFGLPLAFTCPGNQVLAGVQSY  97
BAD97850.1 Dermatopontin1 partial       ----------RAAN--VSEVCSSSGYVNGFGLPLAFTCPGNQVLAGVQSY  38
Ls-SMP-147 comp146160_c2_seq8 complete  YEDRQWEFLCRTAG--TTLACEDSGYVNSFDNPLDFKCPGDKFVTGVSGY  97
BAD97851.1 Dermatopontin2 partial       YEDRQWEFLCRTAG--TTLACEDSGYVNSFDNPLDFKCPGDKFVTGVSGY  58
Ls-SMP-184 comp150744_c0_seq1 complete  KEDRRWEFLCKGVG--NTHSCSDSGYVNTFDNPLVFKCPGDQVITGVNSY  98
BAD97852.1 Dermatopontin3 partial       KEDRRWEFLCKGVG--NTHSCSDSGYVNTFDNPLVFKCPGDQVITGVNSY  58
Ls-SMP-23 comp125384_c0_seq1 complete   KSENKLKLVCDEDDPTELEECEEQWTGMEIWSTITFKCPDGLFLSGMRLP  91
Ls-SMP-24 comp125384_c0_seq2 3'partial  KQGEKLGLICEEEDSSALKGCTEEWTGMEIFGESDFSCPAGKILTGIKQG  91
                                                     .      * ..     :     *.** . .::*:

Ls-SMP-195 comp88616_c0_seq1  complete  HDNQVEDRRFNFRCCDLRGKAPRGCLHGSDVNTWGGKLLLEVPRGKAIKG 147
BAD97850.1 Dermatopontin1 partial       HDNQVEDRRFNFRCCDLRSKAPRGCLHGSDVNTWGGKLLLEVPRGKAIKG  88
Ls-SMP-147 comp146160_c2_seq8 complete  HDNHYEDRRYGFQCCSILGRSPRDCYLTGEVNTWDGKLTLVVDEGKAIKG 147
BAD97851.1 Dermatopontin2 partial       HDNHYEDRRYGFQCCSILGRSPRDCYLTGEVNTWDGKLTLVVDEGKAIKG 108
Ls-SMP-184 comp150744_c0_seq1 complete  HSNKHEDRRFGFQCCNVQGRQPRDCYITGNVNDWDGKLTLAVPEGKAIKG 148
BAD97852.1 Dermatopontin3 partial       HSNKHEDRRFGFQCCNVQGRQPRDCYITGNVNDWDGKLTLAVPEGKAIKG 108
Ls-SMP-23 comp125384_c0_seq1 complete   YYKEHKDLVVKPLCCKIKNEVVRRCR----YLEKTAKKSRIIPTGRVMNG 137
Ls-SMP-24 comp125384_c0_seq2 3'partial  YDAKYKDIIVYPLCCKIKDKVLDMCFPS--GIDVLDTESYHVPPGEVING 139
                                        :  . :*      **.: ..    *           .    :  *..::*

Ls-SMP-195 comp88616_c0_seq1  complete  AVSSHDVTFEDRVWKFQICDI------------------ 168
BAD97850.1 Dermatopontin1 partial       AVSSHDVTFEDRVWKFQICDI------------------ 109
Ls-SMP-147 comp146160_c2_seq8 complete  AHSVHNNYYEDRIWKFEICSI------------------ 168
BAD97851.1 Dermatopontin2 partial       AHSVHNYYYEDRIWKFEICSI------------------ 129
Ls-SMP-184 comp150744_c0_seq1 complete  AYSHHNNRREDRLWQFEICTL------------------ 169
BAD97852.1 Dermatopontin3 partial       AYSHHNNRREDRLWQFEICTL------------------ 129
Ls-SMP-23 comp125384_c0_seq1 complete   FQTEYNHKTKKRSWKWSTCARDKK--------------- 161
Ls-SMP-24 comp125384_c0_seq2 3'partial  FKTEYSRRFERRIWKWLTCYLRKKKSKRFDLINNNRTLK 178
                                          : :.   : * *::  *
```

Supplementary Figure S19.

Alignment of amino acid sequences of dermatopontin of *L. stagnalis*. Bold letters denote sequences detected by LC-MS/MS. Asterisks (*) indicate amino acid residues conserved among the 8 sequences. Colons (:) indicate synonymous substitutions. Periods (.) indicate nonsynonymous substitutions.

Supplementary Table and Dataset legends

Supplementary Table S1 (Dataset 1).

Results of BUSCO evaluation of the mantle transcriptomic data obtained in this study.

Supplementary Table S2 (Dataset 2).

Results of BUSCO evaluation of the foot transcriptomic data obtained in this study.

Supplementary Table S3 (Dataset 3).

Raw datasets of LC-MS/MS results. (a) Data analyzed using soluble fractions extracted from shells of L. stagnalis. (b) Data analyzed using insoluble fractions extracted from shells of L. stagnalis.

Supplementary Table S4 (Dataset 4).

Details of L. stagnalis SMPs identified in this study. This table is based on the output table from Blast2GO. Sequence ID, Contig Name, gene expression levels (FPKM values), protein abundance, logarithms of changes of expression levels between right and left sides of mantle (logFC), significance levels (p-value and q-value), the distinction between soluble and insoluble fractions, theoretical pIs, regions of the longest ORFs (open reading frames), gene model completeness, specificity to mantle tissue, presence or absence of low-complexity regions, and gene type have been added manually. Gene types have been categorized into the following four types: house-keeping (expressed in all tissues), room-keeping (expressed in specific tissues), SMP (genes encoding a known SMP in other taxa), and uncharacterized (genes encoding an uncharacterized protein).

Supplementary Table S5 (Dataset 5).

Raw gene expression data for 35,951 genes in the right and left sides of mantle for each of the three individuals, using Bowtie2 and eXpress.

Supplementary Table S6 (Dataset 6).

Results of differential expression analysis calculated from the data shown in Supplementary Table S4 using edgeR.

Supplementary Table S7 (Dataset 7).

Details of 32 SMPs that indicated a significant difference in gene expression between left and right sides of the mantle.

Supplementary Table S8 (Dataset 8).

Sequences and theoretical pIs of low-complexity regions of 14 SMPs that indicated a significant difference between left and right sides of the mantle in our transcriptomic data.

Supplementary Table S9 (Dataset 9).

Results of RQ scores generated by qPCR and p-values of the exact wilcoxson rank sum test among left and right mantle samples.

Supplementary Table S10 (Dataset 10).

Comparisons of data from Herlitze et al. (2018) and the present study[6].

Supplementary Table S11 (Dataset 11).

Primers used for qPCR.

Supplementary Table S12 (Dataset 12).

Transcriptomic data compared with other shell matrix protein studies[5,7–9].

Dataset 13.

FASTA files of ChtBD2, Laminin_G, WAP, and Tyrosinase domains for phylogenetic analysis.
