# Supplementary material for: Functional shell matrix proteins tentatively identified by asymmetric snail shell morphology
Source: Sci Rep. 2020 Jun 17;10:9768. doi: 10.1038/s41598-020-66021-w (PMC7299971; doi:10.1038/s41598-020-66021-w)
Supplement: Supplementary file 14 — Dataset 13. [file 41598_2020_66021_MOESM14_ESM.docx]

**Alignments of ChtBD2 domains for phylogenetic analysis as a FASTA format.**

>Ls-SMP-88_p1

ECEMNSYRSHWADCTKFIQCSFTDGANVFIKSCRHGMWDTLACDACP

>Ls-SMP-88_p2

DCKYRAPST--TNCRGYWICE--AG-KSVGHCCPPQHYDAVGCVDCN

>Pfu_AYN73066.1_BMSP_p1

RCFFDGYNSDPKSCNEFIQCFY-NGKRAENARCPFRMWDRLLCRSCW

>Pfu_C7G0B5.1_Pif_p1

ECDWDGYGSVPTRCEDFVMCQN-SGSRKTLKSCPFGQWSRTSCVTCS

>Pfu_C7G0B5.1_Pif_p2

DCKLSREYD--VSCRAYWKCE--KG-KSVARCCPSGMYEPKGCVDCD

>Pfu_AKV63183.1_P-U8_p1

--DINGFNPHPNDCAKFTHCVRGDNKVATFRSCPFSQWDTLTCRSCI

>Pfu_AKV63183.1_P-U8_p2

ECRMTHPMTNTDNCRAYWQCK--KG-ISIGTCCKEGQYVPKGCIDCS

>Lgi_XP_009051492.1_LOTGIDRAFT_228264_p1

DCAMNGFNPHPTDCDKYFQCEFSEGLNSVLRQCGQGLWDDLTCNPCR

>Lgi_XP_009051492.1_LOTGIDRAFT_228264_p2

DCQISYKKA--GNCREYYSCS--NG-TSMPECCKKGFYVSQ-CVSCN

>Lgi_XP_009053829.1_LOTGIDRAFT_232022_p1

GCSMNGYNSH-EECHKFVQCFFRGGLGFFVQTCGTGTWNDLTCDMCP

>Lgi_XP_009053829.1_LOTGIDRAFT_232022_p2

DCAEK-YYKLGGNCAEYIECV--NG-RSQPNKCRVGYFVGEICLDCT

>Lgi_XP_009053829.1_LOTGIDRAFT_232022_p3

NCNVG------TKCYYYWT----LGNQGWTMPCPRGTFARCGCVACR

>Lgi_AYN73066.1_BMSP_p1

GCDMKGYNAYPEDCTKYVQCFQ-GNTRSVIRPCPFGTWDNVTCSACR

>Mga_BAK86420.1_BMSP_p1

ECSIKGFNPYPGDCTKYVQCWR-NNRMGAIKSCPFGQWDVMACQSCP

>Lan_XP_013393007.1_LOC106160800_p1

GCVLEGYVLNPYSCTSYWVCIRRRGPDVIERNCSIGTWDDQTCACCK

>Lgi_XP_009066028.1_LOTGIDRAFT_236719_p1

-CDYNYLKDHPTDCNKFLQCNQKDGSDVIVKDCPQGLWDDLLCNPC-

>Lgi_XP_009066028.1_LOTGIDRAFT_236719_p2

DCYDSTYSDIENNCRQYFKCV--NG-ISYLECCESGYFDVRMCSSCT

>Cgi_XP_011456399.1_Pif_isoform_X2_p1

-CDMNGFTRHPTDCSKFIQCYFGNGLKMSYQECPWGNWDSLTCQACP

>Cgi_XP_011456399.1_Pif_isoform_X2_p2

DCLVTYDLP--GSCRSFWACD--GG-ESIPMCCPYGTYHSIGCLDCK

>Cgi_XP_011456398.1_Pif_isoform_X1_p1

-CDMNGFTRHPTDCSKFIQCYFGNGLKMSYQECPWGNWDSLTCQACP

>Cgi_XP_011456398.1_Pif_isoform_X1_p2

DCLVTYDLP--GSCRSFWACD--GG-ESIPMCCPYGTYHSIGCLDCK

>Cgi_XP_011438908.1_SVEP1-like_isoform_X1_p1

DCDINGFNPHPENCYKFVQCYFGKGERSAIRSCPFGQWDDLTCKACP

>Cgi_XP_011438908.1_SVEP1-like_isoform_X1_p2

ECQLSYAMDHVQNCRAFWKCI--LG-KSYGMCCPEGKYKPVGCIDCT

>Cgi_XP_011438908.1_SVEP1-like_isoform_X1_p3

GCDIG----VPEKFEQYV-----PG-GWIPMPCAPGTYNSCGCTNCR

>Cgi_XP_011438913.1_SVEP1-like_isoform_X2_p1

DCDINGFNPHPENCYKFVQCYFGKGERSAIRSCPFGQWDDLTCKACP

>Cgi_XP_011438913.1_SVEP1-like_isoform_X2_p2

ECQLSYAMDHVQNCRAFWKCI--LG-KSYGMCCPEGKYKPVGCIDCT

>Cgi_XP_011438913.1_SVEP1-like_isoform_X2_p3

GCDIG----VPEKFEQYV-----PG-GWIPMPCAPGTYNSCGCTNCR

>Cgi_XP_019922070.1_Pif-like_p1

-CDMNGFNPHPDDCDKFVQCYFGKGQRVAYRQCPFGQWDSLTCRSC-

>Cgi_XP_019922070.1_Pif-like_p2

ECRISYAYSNTDNCRVHWACN--LG-KATATCCAEGSYVPRGCVDCK

>Cgi_XP_019922070.1_Pif-like_p3

GCDIN----KPNKFEQYV-----TG-GWVEMPCAPGTFDNCQCSHCK

>Cgi_XP_011425024.1_Pif_p1

RCNMKGFMKHPEDCDKYVECYFGDGKEAEYRQCPFGMWDEIKCRSCP

>Cgi_XP_011425024.1_Pif_p2

DCSISYPFGNMENCKAHWVCS--HG-KSVPMCCPEGQYVPKGCVQCK

**Alignments of Laminin_G domains for phylogenetic analysis as a FASTA format.**

>Ls-SMP-88

NGDIDESGNLQVGVGGFDGKSIVPRFSNMDGSTLIIKLRYEEAGGQQALVANGDCNRPSSLYMVTGQLRTQGVSVSIPSAGWKEAVLIADDSLQGVTGSQTGSIETSRCAMQIGGTEKHFTGMDVTVY

>Cgi_XP_011422884.1_LOC105325156

KNGVDSSGKVNNGVGAFNGDSLIPRFTNVEGKTFVIRLRYEEKFNEQALVNNGDCGDFGSIQIFTKRVKTKEVSLQIHKGEWKDVEYVVSDGFEGLNGQTKGSVESRQCAVQIGGHGNNFRGLSLEIY

>Pfu_C7G0B5.1_Pif

CDDLDYSGKVENGVGAFNGRALIPRFSGVPGKSVFIKMKYED--------------------------------------------------------------------------------------

>Pfu_AKV63183.1_P-U8

DTDLDKSGNVENGVGAFNGQALIPRFSSFQR-DLVITMKFEN-TGNVPLMSNSNFCKSNISLALIKSFAKDTTTFGLPMGKWNQVYYVHDGKLEGSNGKEKGKLQSTHTPIHIGGKGSKFKGMDIAIY

>Lgi_XP_009051492.1_LOTGIDRAFT_228264

DDDVDKSGNVENGVGGFNGTSLIPRFSNIEGSKVVITMRYAE-IYGQGLISNGDCGKPGSLLVAIDNLQTSGGIVTIPSNGWNEIIYQVEGDLTGVNGSHKGAVKRSQCALQVGATHSNFRGVDLTVY

>Lgi_XP_009053829.1_LOTGIDRAFT_232022

DIDFAEPGEYDNGAGAFDGNSIILRLSNVETTKLSILIRYPF--NNEAVLSNGDCGEEATINLINNGVINENRAALHDIAEWREIEYTFENGIKSMNGSLKILPRRSQCAFQLGSEGKDFRGIDIVVS

>Lgi_AYN73066.1_BMSP

--------------GLYNLRAKRSKRSLSRQ----------------AIISNCNNGEGPSIMVSGGPIKTNTAQLTIPAPGYNDVSMIYDGQLKAVNDASIGKIEMRQAGLLFGCNGANFRGVDVSI-

>Mga_BAK86420.1_BMSP

--------------GGWDMDGFRKRRSAHRKRAIVDKMAH------VALVGNCGNNVKPSISITANELLTN-AELEIPLQGWNEVTMVYDGKLHGVQKHKTGNIVQRK-GLTFGCDKPRFAGLDILLY

>Lan_XP_013393007.1_LOC106160800

----------------LDGRGVMPSFKNSPQTPFTIHFFYLDTAGRIALLNNGKCDIPPSIDIFYSNMTTNGPVGVNKGGRWVCVVMTYNKHMILLDGEERKEMKRTESAWVLGDTFSNFKGFDVSVY

>Pfu_AYN73061.1_VWA_ChtBD_containing_1

TEGFERDKRVVNNVGAFDGTSLVPQLSNVDGEAVVLKIKFETTSNKQAIISNGDCGNNASILVAKDAAETGGTQVEIPKTEWKTVKYAYNDGLQGVNGSSASRIQARPCALQIGGEGGDFQGIEIEIF

>Cgi_XP_011456398.1_PIF_isoform_X1

SDGFERDKRIVNNVGAFSGKSLVPQLSNVDGDSVMLRIRFDSSSGRQALISNADCGNNASILIAKDKAHTNGNQIELPKTEWKNVKYSYNLGLQGVNTKTRGKIQSRPCALQIGGENDDFEGIDLEIF

>Cgi_XP_011456399.1_PIF_isoform_X2

SDGFERDKRIVNNVGAFSGKSLVPQLSNVDGDSVMLRIRFDSSSGRQALISNADCGNNASILIAKDKAHTNGNQIELPKTEWKNVKYSYNLGLQGVNTKTRGKIQSRPCALQIGGENDDFEGIDLEIF

>Cgi_XP_011438908.1_SVEP-like_isoform_X1

DTDTDRSGSVENNSGAFNGEALIPRFANFEN-EIVVAFNYET-SQGTALVSNSDCCTANPTLAVVKSLAKNGTTFQLPLGNWNRVFYIHDTQLEGVNGESKGPVQKTHTAIHIGGKGRNFKGMDFTIF

>Cgi_XP_019922070.1_PIF-like

SSDTDYSGNVENNSGGFNGESLINRFANIDYTDIVVKMRFED-ATGRAMFSNGDCCDNPATMMMVSSMAKEVTTFHIPVGNWNEAYYMHNTMLEGVNGGEKGPIKRSQAALHIGGRGQNFKGMDVAVF

>Cgi_XP_011425024.1_PIF

DNNCDKSGNVENNQGGFDGNALIPRFPEMHASTFVIKMRYDA-TKHQGLLSNGDCEREQPTMFMVKGMAEAKTTFHLPASCWNEAYFIHDFNMEGINGSKRGPIKMAQGGLNIGVRGQNFQGMDVTIY

**Alignments of WAP domains for phylogenetic analysis as a FASTA format.**

>Ls-SMP-149_WAP_p1

GLCPRCIGV---LCRSDRDCRGSQKCCRNCGDNCP

>Ls-SMP-149_WAP_p2

GSCPVCGGI---FCENDYDCPDDRKCCAGCGRLCP

>isotig_7807_WAP

GKCPMFGGDN--ECSTSNLCPRGKVCCNDCKNICT

>Aca_XP_005100260.1_keratin-associated_10-4-like_WAP_p1

GLCPMCAGQ---ECTSDAECAGDEKCCKGCGLRCP

>Aca_XP_005100260.1_keratin-associated_10-4-like_WAP_p2

-TCPACKRERRSPCATDSQCRKKQKCCMSCGRKCA

>Bgr_XP_013065122.1_WAP_p1

GQCPSCRDP---VCRSDYDCGGLQKCCRNCGYNCP

>Aca_XP_005100258.1_spore_coat_SP87-like_WAP_p1

GSCPACNGQ---ECSRDADCPGEEKCCEGCGKRCP

>Aca_XP_005100258.1_spore_coat_SP87-like_WAP_p2

GTCPICRRERRYDCVSDHQCRTNHKCCTSCDRKCE

>Aca_XP_005106735.1_zonadhesin-like_WAP_p1

GSCPACAGR---SCDSDGECPGQEKCCDGCGRRCP

>Aca_XP_005106735.1_zonadhesin-like_WAP_p2

GVCPTCKREERSLCVADTDCKKRQKCCTSCERQCA

>Bgr_XP_013081306.1_fibropellin-3-like_WAP

-SCPVCGGV---FCVLDNECKPEQKCCHSCGGKCV

>Lan_g520.t1_WAP

GACPSECQQ---MCNVDLDCDGDHKCCFNCGTQCP

**Alignments of Tyrosinase domains for phylogenetic analysis as a FASTA format.**

>Ls-SMP-43_p1

-----------------------------------------------------------------------------NGGGELFRSSAIEILSRHEDIITSRYDLEFHAAVHVFVGGMSLDAAFDPIFFMHHAVDIWELFRT

>Ls-SMP-164_p1

----------------------------------------------------------------------------------------------------------VHNLIHALVGGLALSSAFDPIFYLHHSVDIWAIWTA

>Ls-SMP-164_p2

GGYQDLGRFHSCCLHGMPTFPHWHRLWTVQAENALRRGAIPYWDWTQPKSLPDLVTSEKYREKNPFYSITDADKTVRSPRGDLFEQPKFGAIAKLLALEQDEVQFEIHNFIHALVGGMALLTAFDPLFYLHHATDLWAIWQA

>Ls-SMP-164_p3

GTYEKIASFH-CCVHGMPTFPAWHRLYVEQVEEALLGVAVPYWDWTTATKLPDLINQATYFFPNPFFSIAGEGATTRDP-PELFNN----DLYELYALEQDEIQFEIHNALHSLLGGLSLDSAFDPVFFLHHATDIWAIWQE

>Ls-SMP-164_p4

DGWQSIASFHACCLHGMATFPQWHRLYTVQVEDALKRVGIPYWDWSRVDSLPHFIDDENFVQANPWKKIEFENSTEREVVDRLFKRGPHGWLFN------------------------------------------------

>Ls-SMP-165_p1

----------------------------------------------------------------------------------------------------------VHNLIHALVGGLALSSAFDPIFYLHHSVDIWAIWTA

>Ls-SMP-165_p2

GGYQDLGRFHSCCLHGMPTFPHWHRLWTVQAENALRRGAIPYWDWTQPKSLPDLVTSEKYREKNPFYSITDADKTVRSPRGDLFEQPKFGAIAKLLALEQDEVQFEIHNFIHALVGGMALLTAFDPLFYLHHATDLWAIWQA

>Ls-SMP-165_p3

---------------------------------------------------------------------AGEGATTRDP-PELFNN----DLYELYALEQDEIQFEIHNAIHSWVGGLGLHASYDPAFFVHHSTDLWAVWQA

>Ls-SMP-165_p4

DGWASLASFHACCIHGMPTFPHWHRLFTVQVER-------------------------------------------------------------------------------------------------------------

>Ls-SMP-166_p1

----------ACCIHGMPTFPHWHRLLVVQVEDALRRIGVPYWDWTKPTSVPKLAADETYTVRNPFHDIAFLGDTTRDVSSSLSDSPKWGELFDLLALEQDEVQF-------------------------------------

>Ls-SMP-170_p1

----------------MPTFPHWHRLFTVQVERALQRVAIPYWDWTKPTALPEIFTKEDFYVDNPFARIPTENTTVRDIRPELFQTSKDGDLFELSALEQTEVQFEVHNAIHYLVGGLSLESAYDPIFFIHHSVDIWVVWQE

>Ls-SMP-170_p2

NGFQNIASFHACCLHGMANFPQWHRLYVKQWEDALTSVGIPYWDWTTATALPTLVTEET---NNPFHHI-YNGETTRAPRDQLFNDPEFGFFYRLFTFEQTEVQFEIHNAIHSWTGGMSLETAYDPLFLLHHSVDQFAIWQA

>Ls-SMP-170_p3

NGFEAIAGFHACCVHGMPVFPHWHRLFTVQIEQALKSIGVPYWDWTTPKSLPSFFGDDA---HNPFASITAANETTRQVQSELYSERKVHYLFYLTTLEEDEVQFEVHNEIHADIGGMALDSAFDPFFMIHHSIDIWVIWQE

>Ls-SMP-171_p1

KGYAAIAAYHACCVHGMPVFPQWHRLYVVQLEQALKEIGIPYWDWTRPTKLPALVSQQVFIAKNVWYQIEVVENTARALDDRLFQKVEPGDLFELNALMYPEVQFEIHNTIHYLVGGMSLETSYDPIFFLHHSVDIYSIYEA

>Ls-SMP-171_p2

DGYQAIAEFHACCIHGMPTFPHWHRLFTVQVER-------------------------------------------------------------------------------------------------------------

>Cgi_XP_019921271.1_Tyrosinase_isoform_X1_p1

-----MAVFH---AHGGPAFAPWHRIYLLLLETACR-APIPYWDSGLDDPTMSILWSNQFFVNGPFRDTILGT-VIRNYGSALFTKEGLRVLSQYADIAEPMNSLEGHNGPHVWVGGISLNAPWDPVFYMHHAVDVWERFRE

>Cgi_XP_011422333.2_Tyrosinase-like_p1

NEYQAIASLH---AHDGAAFLPWHRIYLLLLETACRGVPVPYWDSTVDDPTRSIVWSEQFFVTGPFRNTTVPGDITREIGNALFTKEGLAVLSRYSEIVEPVYSLEGHNGPHNWVGGLPPWAAFDPVFFMHHAVDVWEVFRA

>Cgi_XP_019923850.1_Tyrosinase-like2_p1

NTYQTLAAFH---AHNGPAFLPWHRIYLLLLETECD-AAIPYWDSGLDDPTTSILWSDQYFVSGPFQDTLLGT-IIRNYGSSLFTKEGLRVLSRFQDISEPIYSLEGHNGPHVWVGGISLNAPWDPVFYMHHAVDVWARFRE

>Lgi_XP_009061494.1_LOTGIDRAFT_166196_p1

NMYDAVAMFH---AHGGPGFLGWHRMYLVMYERALQSVCIPYIDNTIEGDDGSYLWSDEFLVSGPFANTPIG--LTRNVGAFPMDKDILNIMSRIEDIV-------------------------------------------

>Lgi_XP_009061494.1_LOTGIDRAFT_166196_p2

--------------------------------------------------------------------------------------------SRIEDIVSPEHDIEYHGSYHIHVGGMEIDASFDPVFFMHHAIDVWEQFRQ

>Lgi_XP_009054246.1_LOTGIDRAFT_160808_p1

NVLDAFSFLH---AHGGVGFLPFHRVFLYLYEKLLRQVSLCFWDPTLEDYEQSEIWGDRLFVEGFAANTPVG--LIREVGGRTLNEKDIEVLSKLGEISFPSENVEEHNHVHLYIGGMAIEAAYDPIFWFYHVVDLFEEFRE

>Lan_XP_013382611.1_Tyrosinase-like_p1

SEYDIFTAYH---AHFGPAFLPWHREFIKRFETAMQRVTLPYWDPTMDRPQDTIMWTPEYMVSGPAANTPFPVALYRNLTRVPLLPNDLEYAGGLRDLSF-DPSLEFHGRVHDWIGGMGLEAPADPVFYMYHALDWWDDYRQ

>Htu_Q9NDV3_Haemocyanin_type_1_p1

------------------------RLLALQAENALRKGALPYWDWTRPSQLPDLVSHEQYTVHNPWFNIDTVNQTTRSVREDLYQQPEFGDIAQLLALEQDEVQYEIHNFIHALVGGMALRTAYDPIFFLHHSTDIWAIWQS

>Htu_Q9NDV3_Haemocyanin_type_1_p2

GTYESIAQYH-CCVHGMPTFPQWHRLYVVQVENALLNVAVPYWEWTAPDHLPHFIDDATYFYPNPFFRVTFEKRHNKGPTPGSSTQ----YMYELLALEQEEIQFELHNALHSMLGGMSLDSAFDPVFFLHHATDLWAIWQE

>Htu_Q9NDV3_Haemocyanin_type_1_p3

DGYQAIASFHACCVHGMATFPQWHRLYTVQVQDALRRVGIPYWDWTKPNELPELLSSATFYNSNPFLGIEFEGPTERHINERLFHSGDHDWVFELFALEQEEIQFEIHNGIHTWIGGMGLYASYDPIFYIHHSTDIWAIWQE

>Htu_Q9NDV3_Haemocyanin_type_1_p4

DGYQAIAAFHACCIHGMATFPHWHRLYTLQLEQALRRVAVPYWDWTKPTELPHILTDGEYYVANPFARVKIKDATVRNVQESLFKMSSFGLLFDLLALEQTEVQFEVHNTIHYLVGRFSLESSYDPIFFIHHSVDIWAVWQE

>Htu_Q9NDV3_Haemocyanin_type_1_p5

NGFQAIAAFHSCCTHGMATFPHWHRLYTKQMEDAMRAVGLPYWDWTAATHLPTLVTDTD---NNPFQHIDYLNVTTRSPRDMLFNDPEHGVFYRLLALEQTEVQFEIHNAIHSWTGGMSLDTAYDPLFWLHHSTDIWAVWQA

>Htu_Q9NDV3_Haemocyanin_type_1_p6

GGFEAIAGYHPCCVHGMPVFPHWHRLHTIQMERALKNLGIPYWDWTKKSSLPSFFGDSS---NNPFYKIRGVQHTTRDVNQRLFNQTKFGYLYYLQVLEENEVQYEIHNAVHSWLGGMSLESAFDPVFMIHHSLDIWILWQK

>Htu_Q9U5P3_Haemocyanin_type_2_p1

GVYENIAKFH-CCVHGMPTFPQWHRLYVLQVENALLEVSVPYWDWTETTELPSLIAEATYFFPNPFFRISFENATTRDP-PELYVN----YYYQMLVFEQDEIQFEMHNVLHAWLGGISLDSAFDPVFFLHHATDLWAIWQE

>Htu_Q9U5P3_Haemocyanin_type_2_p2

DGFQAIASFHACCVHGMATFPQWHRLYTVQFQDALRRVGVPYWDWLRPSHLPELVTMETYHDPNPFYQIEFEGETEREVIDKLFVKGGHVWFFKILALEQEEIQFEIHNGVHTWVGGIGLHAFYDPLFYLHHSTDIWAIWQE

>Htu_Q9U5P3_Haemocyanin_type_2_p3

NGYQALAAFHACCIHGMPTFPQWHRLYTLQLEMALRRVAIPYWDWTKPSELPSLFTSPEYYVNNPFSKVKFANTTVRDPQEMLFQLCEHGILYELLALEQTEVQFEVHNVIHYLVGGLSLHASYDPFFFIHHSVDMWVVWQA

>Htu_Q9U5P3_Haemocyanin_type_2_p4

NGYQAIASFHACCAHGMASFPHWHRLYVKQMEDALADIGIPYWDWTTATELPALVTDSE---NNPFHEIDHLGVTSRSPRDMLFNDPEQGFFYRLLALEQTEVQFELHNAIHSWTGGMSLETAYDPLFWLHHSTDIWAVWQA

>Htu_Q9U5P3_Haemocyanin_type_2_p5

TGYEAISGYHPCCVHGMGIFPYWHRLLTIQLERALEHLGVPYWDWNKDSSLPAFFSDSS---NNPYFKIAGVGHTVREPTSLIYNQPQIHYLYYLTTLEENEVQYEIHNAVHSWLGGMSLESAFDPVFMILHSLDLWIIWQE

>Edo_Q5XLV2_Haemocyanin_A-type_p1

TGYQAIAAYHVCCLHGMPTFPFWHRLYTVQFEQTMVGLGVPYWDWTQPNHLPELVSHPLFMARNVFYSIAFEKKTARAVDTRLFQASKGGFLLELSALEQDEVQFEVHNPIHYLVGGMSLEMAYDPFFFLHHSVELFTIWQA

>Edo_Q5XLV2_Haemocyanin_A-type_p2

DGYQALAEFHACCVHGMSTFPHWHRLVVMQFEDALLAIGVPYWDWTTPSSLPHLVAVETYEVPNPFYHIEFLHNTARNVDSRLFEKPTKGYLHDLLALEQEEVQLEVHNAIHAWVGGMSLHTSFDPLFWLHHSVDLWAVWQA

>Edo_Q5XLV2_Haemocyanin_A-type_p3

SGYQQVAAFHACCHHGMATFPHWHRLLTINFENGLRHNGIPYWDWTRPSELPTLVKDETYA-PNPFFSIDEIGETTRFPNPTLFLKPPFGPLGNMYALEQEEVQFEIHNHIHALVGGMSLETAFDPIFILHHSVDIWAIWQA

>Edo_Q5XLV2_Haemocyanin_A-type_p4

GRYEEIASFHTCCIHGMPTFPHWHRLYVALVENELLAVAVPYWDWVQPDHLPALVNRATYYVPNPFFKISFLNSTNRDP-EKLFGN----YLYELFVLEQTEVHFEVHNTIHSWLGGMSLDAAYDPIFFLHHSIDIWAIWQE

>Edo_Q5XLV2_Haemocyanin_A-type_p5

DGFEAIASFHACCLHGMATFPHWHRLYVVQFEQALHRVGVPYWDWTRPSKIPDFIASEKYSEYNPFNHISFISETKREVSEYLFEHPVLGWLFDALALEQTEIQLEIHNAIHSWIGGLNLHAAYDPIFYLHHSVDLWVIWQE

>Edo_Q5XLV2_Haemocyanin_A-type_p6

HGFQTIASYHACCLHGMPVFPHWHRVYLLHFEDSMRRVATPYWDWTQPTKLPRLLADSDYYVENPFLRIKSEDTTVRDVKPELFEI-GGGTLYRLLMLEQEEVQFEVHNSIHYLVGGMSLVSSFDPIFYVHHSVDLWAIWQA

>Edo_Q5XLV2_Haemocyanin_A-type_p7

NGYQKIASYHACCQHGMVTFPNWHRLLTKQMEDALVAVGIPYWDWTTTANLPVLVTEEK---DNSFHHIDVANTTTRSPRAQLFDDPEKGFFYRALALEQTEIQFEIHNAIHSWVGGMSLHTSYDPLFYLHHSTDIWSVWQA

>Edo_Q9BJ58_Haemocyanin_G-type_p1

TGYQAIAAYHVCCLHGMPTFPLWHRLYTVQFEQTMVGLGVPYWDWTQPNHLPELVSHPLFMAKNVFYSIAFEKKTARAVDTRLFQASKGGFLLELSALEQDEVQFEVHNPIHYLVGGMSLETSYDPLFFLHHSVELFTIWQA

>Edo_Q9BJ58_Haemocyanin_G-type_p2

DGYQALAEFHACCVHGMSTFPHWHRLVVMQFEDALLAIGVPYWDWTTPSSLPHLVAVETYEVPNPFYHIEFLHNTARNVDSRLFEKPTKGYLHDLLAFEQEEVQFEVHNAIHAWVGGMSLHTSFDPLFWLHHSVDLWAVWQA

>Edo_Q9BJ58_Haemocyanin_G-type_p3

SGYQQVAAFHACCHHGMATFPHWHRLLTVNFENGLRHNGIPYWDWTRPSELPTLVKDETYA-PNPFFSIDEIGETTRSPNPTLFLKPPFGPLGDMYALEQEEVQFEIHNHIHALVGGMSLETTFDPIFILHHSVDIWAIWQA

>Edo_Q9BJ58_Haemocyanin_G-type_p4

GRYEEIASFHTCCIHGMPTFPHWHRLYVALVENELLAVAVPYWDWVQPDHLPALVNRATYYVPNPFFKISFLNSTNRDP-EELFGN----YLYELFVLEQTEVHFEVHNTIHSWLGGMSLDAAYDPIFFLHHSIDIWAIWQE

>Edo_Q9BJ58_Haemocyanin_G-type_p5

DGFEAIASFHACCLHGMATFPHWHRLYVVQFEQALHRVGVPYWDWTRPSKIPDFIASKRYSEYNPFNQISFISETKREVSEYLFEHPVLGWLFDALALEQTEIQLEIHNAIHSWIGGLNLHAAYDPIFYLHHSVDLWVIWQE

>Edo_Q9BJ58_Haemocyanin_G-type_p6

HGFQTIASYHACCLHGMPVFPHWHRVYLLHFEDSMRRVATPYWDWTQPTKLPRLLADSDYYVENPFLRIKSEDTTVRDVKPELFEI-GGGTLYQLLMLEQEEVQFEVHNSIHYLVGGMSLVSSFDPIFYVHHSVDLWAIWQA

>Edo_Q9BJ58_Haemocyanin_G-type_p7

NGYQKIASYHACCQHGMVTFPNWHRLLTKQMEDALVAVGIPYWDWTTTANLPVLVTEEK---DNSFHHIDVANTTTRSPRAQLFDDPEKGFFYRALALEQTEIQFEIHNAIHSWVGGMSLHTSYDPLFYLHHSTDIWSVWQA

>Equ11340

NKYDAIALLH---AHLGDGFLGWHRLYMNMFEAAMQEVCLPYWDTSLEDPALSNLWTPEFMVDGPFANTPGG--LIRNVDVDLVTATNIRILTRYEQITF------------------------------------------

>Equ11343

NRYDAIALFH---AHGGAGFFGWHRLYLNMYETALQEVCLPYWDNSLDDITRSNIWTPAFMVDGPFANTPRA--LIRNFGGEPMTVTTVQILTRYEDITS-RYDLEFHGAVHMLVGGMTLNASFDPIFFLLHAIDIGSA---

>Equ14143

NVHDAYSYLH---AHGGPAFLPYHRVFIFLYEKLLRIISLCYWDTTLENILWSSSWTPELF-------STAS-----ETG-RAPRKDDQ----------------------------MTTKSCWTP-----STTGSWGRWRR

>Equ24617

--------------------------------------------------------------------------LIRNVGGELMTVTTFQILTRYQDIIFRRYNLEIHGGIHVFVGGMSLDAAFDPVFFQVHAIDKFEQFRX

>Equ32293

NQFRS--------SRV----LGWHRLYITLFETALQEVCLPYWDVSLDDPTLSNLWTPAFMV--------------------------------------------------------------------------------

>Equ33769

--------------------------HILSASATIR-------------------------VP-----LP-----------------------------------------HVW--GMSLDASFDPIFFLLHAIDLWELFRT

>Lgi_V3ZAB2_SMP

NMYDAVAMFH---AHGGPGFLGWHRMYLVMYERALQSVCIPYIDNTIEGDDGSYLWSDEFLVSGPFANTPIG--LTRNVGAFPMDKDILNIMSRIEDIVS-EHDIEYHGSYHIHVGGMEIDASFDPVFFMHHAIDVWEQFRQ

>Cgi_K1PS92_SMPs

NVYDALAGVH----RFGCGFLGFNRIFLLRLEMAMRQITIPYWASTLEDPTVSVVWSPEFFVTGPFGNDGTG--SRNLNH--VLTESDIEVLERHHDIFP-TKDFANNYNVHGYVGGMNLDAAYDPVFIFHYAIDLFEKARE

>Pmarg-Tyrosinase1_SMP

SRYDTIAGLH---AHMGANFLGWHRLYLDMFEMALQEVVLCYWDSTLDSQVNTVSFSAELFVNGPFRFLPGG--LQRFIAGSSLTRPVVDIATDINTNSQRGQGFPDHNNTHVWVGGMQVSSPQDPVFWFHHTVDVWELFRQ

>MgaCL4852Contig2_SMP

NKYDSFAIMH---AHDGPNFVSWHRYFLVLFENALREVTLPYWDSRADNKEDSILFTEIFLVSGPFAFTPTKP-LRRELGGSPVHPERLKVFTKHREILR-YANLESHDNVHRWVGGMSILSPMDPVFWLLHCVDLWEKFRE

>Villosa_30664_Tyrosinase_SMPs

GEYDTFARIH---FHDGPNFLGWHRIYLAYFEEAVRRLSLPYWDYTLDDPTQSVLWSATFLVSGPFSGVNGS--LIRNTGGALMSKQDVDVLTRTSEITFTIYNLEIHNRVHNWVGGMELDAAFDPAFFLHHAVDVWELFRL

>Lgi_V4A5X3

NSFWSIASYH---QHSNVLFPTWHRFYCLRMEQALQTVALHYWDTTSELVQPTVTIDGPSIFVKPEGYMIVNP-KIREVQ-ELLQENIY-FINNYPGQDDY--NFSNHNDIHLSIGGEMNEAAFDPIFFSHHSIDVFWIWQR

>Lgi_V4AN59

NVLDAFSFLH---AHGGVGFLPFHRVFLYLYEKLLRQVSLCFWDPTL-DYEQSEIWGDRLFVEGFAANTPVG--LIREVG-RTLNEKDIEVLSKLGEISF-SENVEEHNHVHLYIGGMAIEAAYDPIFWFYHVVDLFEEFRE

>Cgi_K1PY25

SNYETIANYH---AHGGPNFPGWHRVFLLIYEEALIESGIPYIDWRLDSPGSSILWTNEYLVNGPFENINRT--LVRNVG----QRDP----TNVAELFATARSFADHNIGHIYVGGMNLRATYDPVFFMHHCIDLWWRYQC

>Cgi_K1QHI1

STYDLIGSMH---MHNGPGFFPRHMLMMLIMETACH-SPMHYWDMTMDVPTDSIMFSEEFYVTGPFAHTPIG--IIRNIGGESLAGKGIRMLSRLAEISE-AYSVEVHNGVHNWIDGMALNAWFDPIFYGIHSFTIWIAFKG

>Cgi_K1Q7A7

STYDLIASLH---MHNGPAFLPRHMVYLLVMETACR-VPMPYWDMTTDDPTTSIVWSDLFFVTGPFGRTPTG--IIRNIGGASLARKGIRLLSRTFEISE-IFSIEVHNGVHNYIDGMSLNASWDPVFWFIHSFQLWVAFRN

>Cgi_K1QPB3

GRYDALANMH---GHEGPGFLAWHREYLTAFETALRRVSLPYWDSSKDNPALTSFFSSALVVNGPFAGAPDG--LSRDIGGSLFTPEGLDFLNDVNLTRQMANTLEGHNNVHNWVGGMSLNAAHDPVFFMYHAVDVWERFRE

>Cgi_K1PS50

NLYDILCNFH---AHFGPAFLSWHRVSLYIFEQALRSVSLPFWDSRLENPSATSLFTDELMVTGHFANHPAG--LVRNVGGEPIQRRDIELLKATKEFMF-HMNLELHGKVHMWVSGMNLNSPADPIFWMHHCIDVWEKIRQ

>Cgi_K1R2H1

SKYDVLASFH---AHGGCNFLGFHRLYTLMYEEALTQVTLPYLDSTLDNSANSMIWHDDFLVSGPFANTTVG--LFRNVGGRPMDDEEIKVTSRMSEICG-WHDLEFHGPFHLFVDGMGIEASEEPVFWMHHAIDVWELQRE

>Cgi_K1RLT5

SRYDTISSFH---AHFGCHFAGFHREYLKVYEMALQEVTLPYWDSLLDDSASSLMFSPQFMVSGPAANTETG--LRRNVGGELYLYEEIQITTRFVEICG-NHGLEFHGDIHLWVGGMALNAADDPLFWMHHAVDIWELQRQ

>Cgi_K1QL10

NKYDLLANIH---AHGGPGFLGWHRVFLLLFENALRQATLPYWDCTLDHPSESVIWSDLFLVTGPFRGTQFG--LHRQVSRHLMSVHDLRILEELGNISY-SKNLEQHNNVHVWVGGMRIEGAFDPVFYVLHTIDVWEDFRV

>Cgi_K1P4E2

NTYQTLAVLH---AHGGP-----------ALETACG-SPIPYWDSTVDDPTMSILWSEQFFVTGPYQSTILG--LVRNIGSPLFTKEGLQVLSRYSEIVE-IFSLEGHNGPHTWVGGLATNAAYDPIFFNHHAIDVYELFRQ

>Cgi_K1PAV4

NEYQAIASLH---AHDGAAFLPWHRIYLLLLETACRGVPVPYWDSTVDDPTRSIVWSEQFFVTGPFRNTT-PG-ITREIGNALFTKEGLAVLSRYSEIVE-VYSLEGHNGPHNWVGGLPPWAAFDPVFFMHHAVDVWEVFRA

>Cgi_K1PI66

NTYQTLAAFH---AHGGPGFAPWHRIYLLLLETACG-APIPYWESGLDDPTASILWSDDFFVTGPFRSTILG--IIRNYGGALFTKTGYNVLSRYDDISE-FFTLEGHNGPHTWTGGLAPNAPYDPVFYMHHSVDVYEAFRQ

>Cgi_K1QAP4

NTYQTMAVFH---AHGGPAFAPWHRIYLLLLETACR-APIPYWDSGLDDPTMSILWSNQFFVNGPFRDTILG--VIRNYGSALFTKEGLRVLSQYADIAE-MNSLEGHNGPHVWVGGISLNAPWDPVFYMHHAVDVWERFRE

>Cgi_K1QWH8

----------------------------------------------MDRPQESAMFTSQLVVNGPFAGEDGG--IVRQIANSSLMVKRMEFFEGALRHRDDRYTIEGHGNVHNWVGGMSPFTAFDPVFILHHTIDIWEKFRQ

>Cgi_K1Q1M0

NVYDWFCNLH---AHYGPAFFGFHRIYLYL-QQQLRTTFLPFWDSTYETPTSSVLFTEDFLVDGPFKNHDVG--LIRNTAGQLFQRETIDIMMKMSDISN-DVNLEAHGQVHAWIGGMDLDSPADPIFYMHHCVDIWERFRD

>Cgi_K1RE44

SIYDLIGSLH---MHNGPGFLGRHTLYVLALETACR-TPIPYWDFMMDDPTSSAVWSNTFFVTGFCGNTPQN--IIRNVGGVRLPRRALRLMSRTSEITE-VFSIEVHNAVHNHIDGFSLDSPFDPVFWFLHSFHMWYMFKN

>Cgi_K1RLR4

-------------GHGGPNFLGFHRVYLLFFEFALQRVSLCYWDSTMDEPQETAMFTSQLVVNGPFANEDDG--LTRNIARSSLMVKALNFFEGATTHRDNANTIEGHNNVHNWVGGMAAVTAYDPVFILHHTIDVWERFRQ

>Cgi_K1R932

NTYDLIGSLH---MHNGPGFLGRHSLYVLAMETACR-TPIPYWDFMMDDPTSSAIWSNTFFVTGFCGNTPQN--IIRNVGGLQLPRRVLRILSRTSEITE-VFSIE------------------------------------

>Cgi_K1RFL7

NTYDLIGSLH---MHNGPGFLGRHALYVLAMETACR-TPIPYWDFMMDNPTSSAIWSNTFFVTGFCGNTPQN--IIRNVGGVRLPRRALRILSRTREITE-MFSIEVHNAVHNYVDGFSLDSTFDPVFWFLHSFHMWYMFKN

>Cgi_K1Q6C9

NRYDVIAALH---AHEGPNFMGWHRIYLIVYENALRQVTIPYFAGDLDDSTQSVLFCERFFVSGPYANTPSG--LVRNYGGELWTREGLQILNKNAEIIA-EDNLEDHGAIHNWIGGIGLQSSQDPAFFSLHAVDIWEEFRK

>Cgi_K1QE57

NRFDALGLLH---VHHGAAFLSFHRVLLLIFENALRQVALLYFDSRLDDPTRSIIWSPQFLVDGPFRFTPAG--LVRAGGGEYFTYRHIRVMTRLEEISEPPFDFEIHGDVHQHIGGMAAEAGFDPVFYMHHCVDLWEVFRR

>Cgi_K1QMD1

NKYDSLAALH---AHGGPNFLGWHRVFLIMFENALREVTLPYWDSTIDNSVASAIWSDRFLVNGPFADTPAG--LIRNIAGQLFRRNVIQILTRLSEITEFQFNLEFHGEVHMWVDGMGLTAAMDPVFFLHHAIDVWEKFRA

>Cgi_K1Q6D2

NKFDAIASLH---AHGGPGFLGWHRIYLTLFENALREVTIPYWDNTLDDPRRSIMWSPLFFVTGPFRRTPYG--LRRDIGRRLMSKTDLEVFSRMWEISN-RYNLELHNHVHVWIGEMSIESSYDPAFFSHHAVDLWEEFRQ

>Cgi_K1QXR1

NRYDALGLVH---IHHGGAFLAWHRLFITIFENALRQVTLPYWDSTMDDPTQSVTWSPQFLVTGPFAFTPNG--LIRNVGGQLLSRQAIMVLSRMAEITEPQYNIENHGDAHTWIGGMEMESAFDPVFYLHHAVDVWEIFRQ

>Pmax_P86952

NEYDTLANLH---AHDGSNFLGWHRVYLMYYERALRRVTLCFWDTTMENWEYTAVFSSDFFVTGPFRDLPPG--LYRNMTGMPFDSRAASIFYNTSTITDTNITIEGHNNVHNWVGGMGLDAPQDPIFFFHHCIDVWERFRE

>Pmarg_H2A0L0

SRYDTIAGLH---AHMGANFLGWHRLYLDMFEMALQEVVLCYWDSTLDSQVNTVSFSAELFVNGPFRFLPGG--LQRFIAGSSLTRPVVDIATDINTNSQRGQGFPDHNNTHVWVGGMQVSSPQDPVFWFHHTVDVWELFRQ

>Pmarg_H2A0L1

NEYDTLANLH---AHDGSNFLGWHRVYLMYYERALRRVTLCFWDTTMDNWEYTAVFSSDFFVTGPFRDLPPG--LYRNMTGMPFDSRAASIFYNTSTVTDTNITIEGHNNVHNWVGGMELDAPQDPVFFFHHCIDVWERFRE

>Pmax_A0A024CJP7

NEYDTLANLH---AHDGSNFLGWHRVYLMYYERALRRVTLCFWDTTMENWEYTAVFSSDFFVTGPFRDLPPG--LYRNMTGMPFDSRAASIFYNTSTITDTNITIEGHNNVHNWVGGMGLDAPQDPIFFFHHCIDVWERFRE

>Pmax_A0A024CI03

NRYDALGLLH---VHHGAGFLGFHRVLLVVYENALRQVTLPYWDSRLDDPTRSIIWSPQFLVNGPFAFTPAG--LVRNGGGELFTYNHIRVMTRLEEISEPPFDFEIHGDVHQMVGGMAAEAGYDPVFFLHHCVDLWEVFRR

>Pmax_A0A024CHU4

NEYDTMAHTH---SHDGSNILGWHRLFLFLFEIALRRVVLCYWDSSLDGQVQSAAFSHELFVTGPFANTPWG--LRRNFGGGSLVRPIVDIERDIRSHGQDATGFTDHNNAHVAVGAMAPNAAYDPLFFFHHCIDVWELFRR

>Pmax_A0A024CHG7

NEYDTLANLH---AHQGSNFLGWHRVYLMYYERALRRVTLCFWDTTMENWEYTAVFSSDLFVTGPFRDLPPG--LYRNMTGIPFDSRAASIFYNTSTITDTNITIEGHNNVHNWVGGMEIKAPQDPIFFFHHCIDVWERFRE

>Pmax_A0A024CIJ0

NKYDALASLH---AHGGPGFLGWHRVYLVLFENALREVTIPYWDSTLDDPRRSIIWSPLFLVNGPFRRTPYG--LRRDIGRRLMNRQDIQVFSRLWEITN-EYNIELHNHVHVWVGEMSIESSYDPAFFAHHAIDLWEEFRQ

>Pfu_Q287T6

NRFDTMARIH---AHDGSSILGWHRVFLYLFENALRRVVLCYWDSTIDGQAQSSSFSH---VTGPFANTPWG--LRRNFGGGSLMRPVVDIASDIRSHGQQATGFIDHNNAHVAVGAMAPNAAWDPLFYFHHCIDVWQLFRR

>Pfu_A1IHF0

SRYDTIAGMH---AHLGPSFLGWHREYLIMYEEALQEVVLCYWDSTLDMPGSTQRFTVAFSVNGAFANLPGG--AGDRFPPPSLTRPIVDIATDITSHTQRGTGFTDHDNTHVWVGEMQVAAPGDPVFFFHHTIDGWELFRQ

>Pfu_A7BK18

SRYDTIAGMH---AHLGPSFLGWHRVYLIMYEEALQEVVLCYWDSTLDMPGSTQRFTVAFSVNGAFANLPGG--ARAQFPPPSLTRPIVDIATDITSHTQRGTGFTDHDNTHVWVGEMRVAAPGDPVFFFHHTIDGWELFRQ

>Pfu_A1IHF1

SRYNTLAAMH---AHNGPNFLGWHRVYLNMYEEALQEVALCYWDSTLDMPGDSQRRTVAFSVNSQFANLDNT--LRRMIGNSSLTRPIVDILTDINRHRWSRFGFIDHDNTHVWVGGMVERSPEDPVFWFHHLIDVWELFRR

>Pmax_A0A024CIJ4

NEYDTMAHTH---SHDGSNILGWHRLFLFLFEIALRRVVLCYWDSSLDGQVQSAAFSHELFVTGPFANTPWG--LRRNFGGGSLVRPIVDIERDIRSHGQDATGFTDHNNAHVAVGAMAPNAAYDPLFFFHHCIDVWELFRR

>Pmax_A0A024CI05

SAYDFFAGLH---AHIGSNFLGWHRVYLWYFERILIRVPLCYWDSTLDGQRNTTMFTSEVVVNGPFRNIPDR--LRREIAFASLMRPVVDIMTSIRNHSQASVGMIDHDNTHVWVGGMSATAPQDPVFWLHHTIDVWEKFRE

>Pmax_A0A024CHH0

SMYDTFARVH---AHGGSNFLGWHRLYVLFFENALRRLVLCYWDPTLDLQIHSVTFSDRLFVNGPFKNLPYN--LRRNIGEGSLTRPVIDITLNIIRSTQLAIGFKDHDNTHVYVGEFSLPTAQDPIFWFFHAVDVWELFR-

>Pmax_A0A024CJQ0

SAYDFFAGLH---AHIGSNFLGWHRVYLWYFERILIRVPLCYWDSTLDGQRNTTMFTSEVVVNGPFRNIPDR--LRREIAFASLMRPVVDIMTSIRNHSQASVGMIDHDNTHVWVGGMSATAPQDPVFWLHHTIDVWEKFRE

>Pmax_A0A024CHU9

SQYDTIAGLH---AHQGANFLGWHRIYLNMFETALQEVVLCYWDSTLDTQLNTVTFSAELFVNGPFRNLPGG--LQRFIAGSSLTRPVVDIATDINSHSQGGQGFPDHDNTHVWVGGMQVLSPQDPVFWFHHTIDVWELFRQ

>Pmax_A0A024CIJ8

NLFDSLAAVH---AHFGPNFLGWHRIYLYYYEIALRRVALCYWDSTLDSPERSVMFSSEYFVEGPFRDLPNR--LRRQINGSLMVYDGVRILTDLIRTANPQRTIEGHNNVHVWVGQMSVDAPQDPVFYFHHCIDFWERYRE

>Pmax_A0A286K027

SRYDTIAGMH---AHLGPSFLGWHRVYLIMYEEALQEVVLCYWDSTLDMPGSTQRFTVAFSVNGAFANLPGG--AADRFPPPSLTRPIVDIATDITSHTQRGTGFTDHDNTHVWVGEMQVAAPGDPVFFFHHTIDGWELFRQ

>Lan_g6403

SEYDIFTAYH---AHFGPAFLPWHREFIKRFETAMQRVTLPYWDPTMDRPQDTIMWTPEYMVSGPAANTPP---LYRNLTRVPLLPNDLEYAGGLRD----DPSLEFHGRVHDWIGGMGLEAPADPVFYMYHALDWWDDYRQ

>Lan_g6405

REYDIFVGYH---AHFGPAFLPFHRELLFRLERALQRVALPYWNSTMDNAEDSIMWTEEFAVTGPFANTTEG--LFRNLT-PGLMADD---DSAFRD----------HGKTHNWVGGMILESPGDPVFFLHHAIDAWEEFRE
